# Supplementary material for: JAK/STAT inhibition protects glucocorticoid receptor knockout mice from lethal malaria-induced hypoglycemia and hyperinflammation
Source: EMBO Mol Med. 2025 Jul 23;17(8):2040–70. doi: 10.1038/s44321-025-00264-w (PMC12340059; doi:10.1038/s44321-025-00264-w)
Supplement: Supplementary file 1 — Appendix [file 44321_2025_264_MOESM1_ESM.pdf]

JAK/STAT inhibition protects glucocorticoid receptor knockout mice from lethal malaria-induced hypoglycemia and hyperinflammation

Fran Prenen[1#], Leen Vandermosten[1#], Sofie Knoops[1], Emilie Pollenus[1], Hendrik Possemiers[1], Pauline Dagneau de Richecour[1], Giorgio Caratti[2,3], Christopher Cawthorne[4], Sabine Vettorazzi[2], Yevva Cranshoff[1], Dominique Schols[5], Sandra Claes[5], Christophe M. Deroose[4], Uwe Himmelreich[6], Jan Tuckermann[2], Philippe E. Van den Steen[1*]

APPENDIX

Appendix Table of Contents

1. Appendix figure S1 page 2
2. Appendix figure S2 page 3
3. Appendix figure S3 page 4
4. Appendix figure S4 page 5
5. Appendix figure S5 page 6
6. Appendix figure S6 page 7
7. Appendix figure S7 page 8
8. Appendix figure S8 page 9
9. Appendix figure S9 page 10
10. Appendix figure S10 page 11
11. Appendix figure S11 page 12
12. Appendix figure S12 page 13
13. Appendix figure S13 page 14
14. Appendix table S1 page 15
15. Appendix table S2 page 16
16. Appendix table S3 page 17
17. Appendix table S4 page 18
18. Appendix table S5 page 19

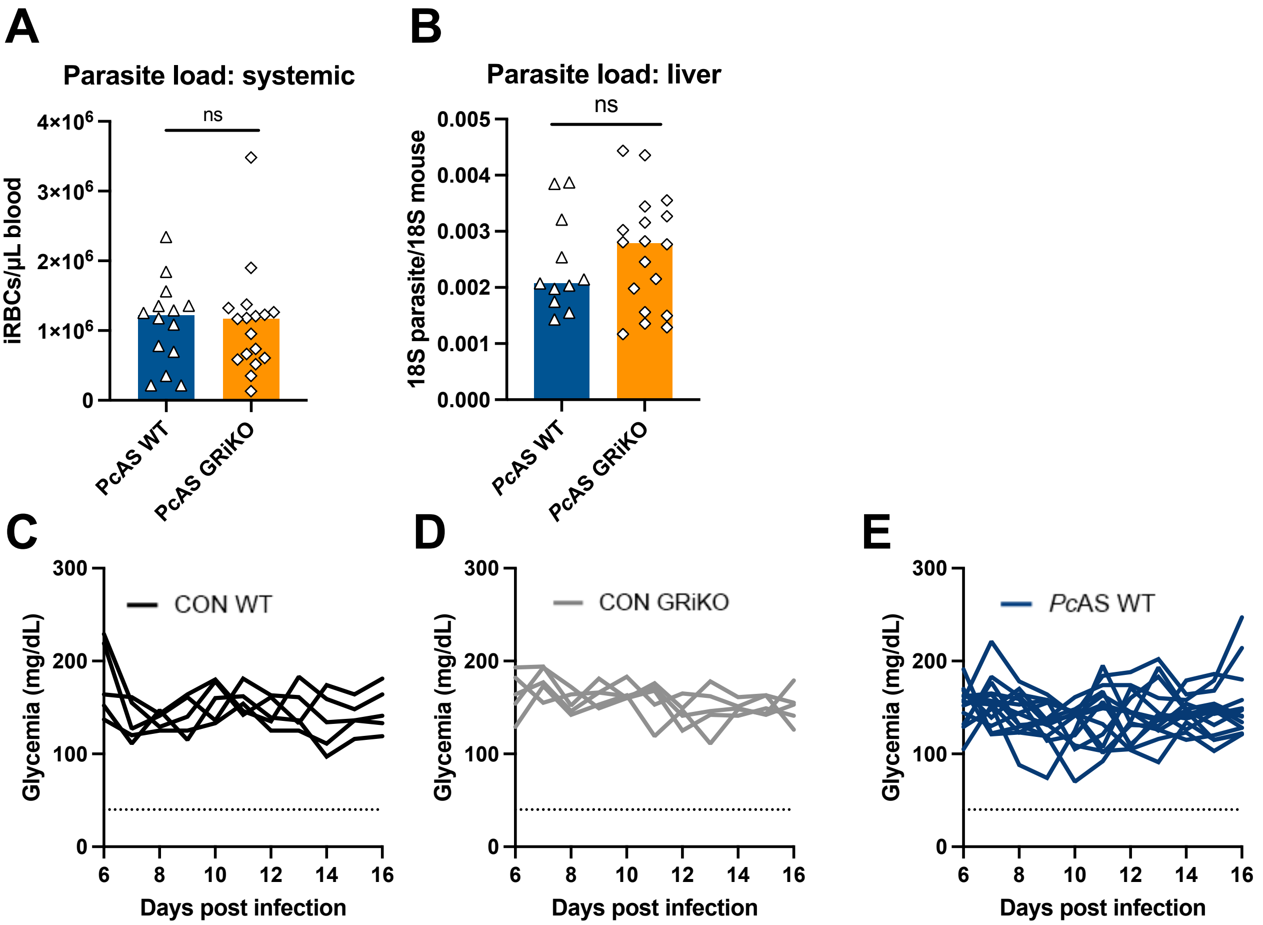

Fig. S1. Similar parasite load between infected WT and GRiKO mice and no change in glycemia levels in uninfected mice and *Pc*AS-infected WT mice. WT and GRiKO mice were infected with 10^4 *Pc*AS parasites and glycemia levels were measured from 6 dpi until 16 dpi. Parasite load was determined by calculating the **(A)** number of infected red blood cells (RBCs) per microliter blood (systemic parasite load) and **(B)** assessing expression levels of parasite-specific 18S in liver tissue at 10 dpi in infected WT and GRiKO mice. Glycemia levels of **(C)** uninfected WT mice, **(D)** uninfected GRiKO mice and **(E)** *Pc*AS infected WT mice are shown (dotted line indicates 40 mg/dL). For **A-B**: Each dot represents an individual mouse and statistical differences were calculated with the Mann-Whitney *U* test. For **C-D**: Each line represents an individual mouse. Data from two-three independent experiments.

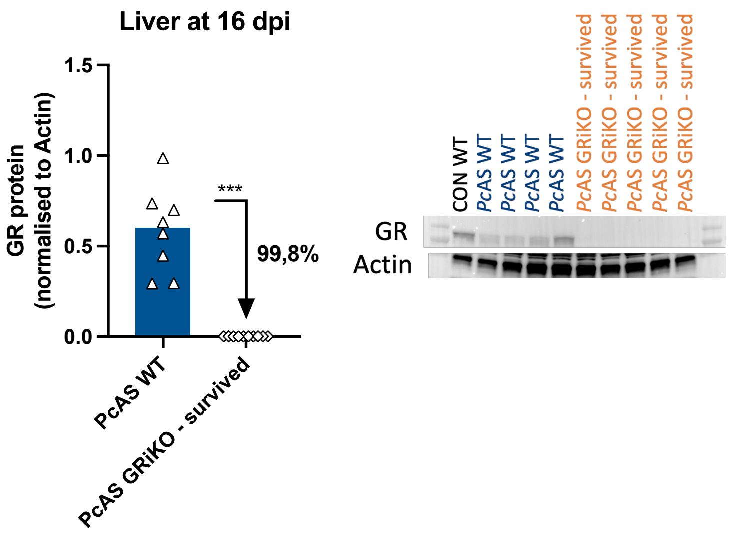

Fig. S2. Western blot analysis confirms the absence of GR in livers of surviving GRiKO. WT and GRiKO mice were infected with 10^4 *Pc*AS parasites. At 16 dpi, livers were collected from WT and GRiKO mice that survived the infection to assess GR levels with western blot. Representative blots are shown together with the semiquantitative determination of band intensities. Bar graph represent median per group and each dot represents an individual mouse. Statistical differences were calculated with the Mann-Whitney *U* test. ***: p<0.001. Data from two independent experiments.

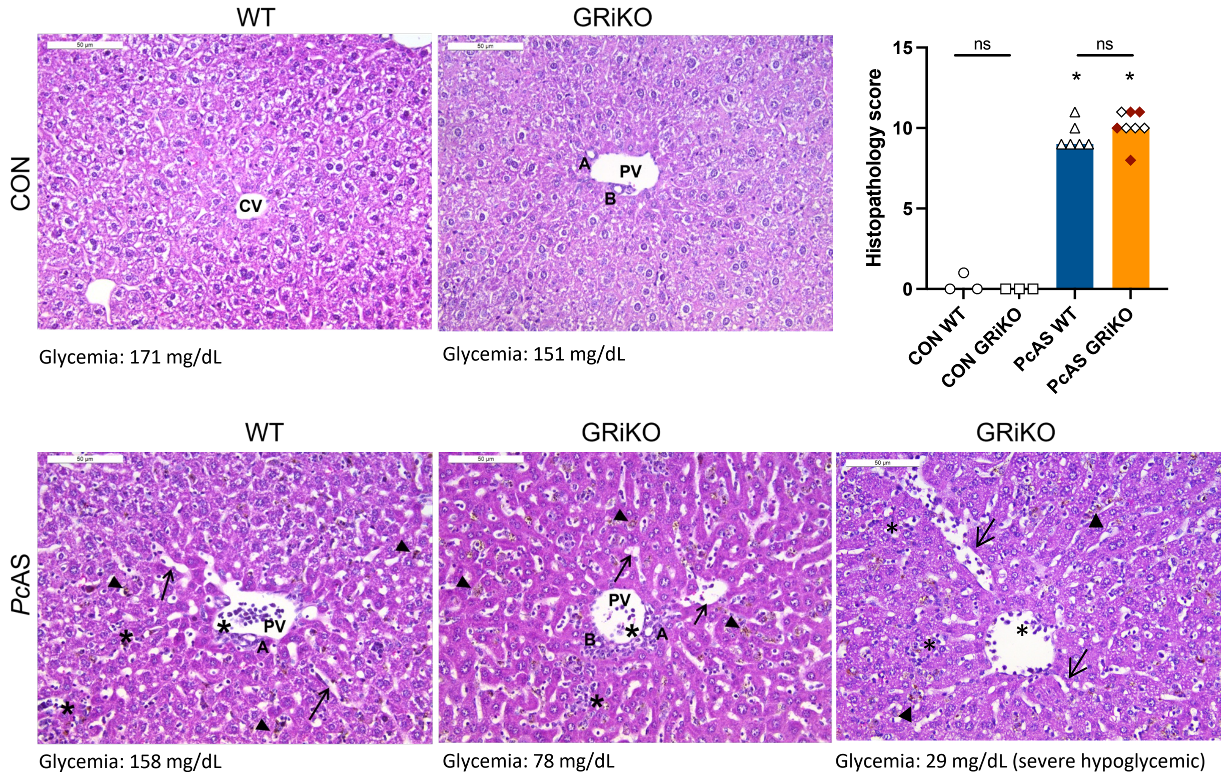

Fig. S3. Inflammatory infiltrates in livers of infected WT and GRiKO mice. WT and GRiKO mice were infected with *Pc*AS parasites. Infected mice were euthanized and dissected at 10 dpi. Paraffin-embedded sections were prepared from livers of uninfected (CON) and infected (*Pc*AS) mice and stained with H&E staining. Representative images are shown (scale bars: 50µm). CV, central vein; PV, portal vein; A, hepatic artery; B, biliary duct; *: inflammation, ↑: sinusoidal dilation, ▲: hemozoin. For the bar graph: Each datapoint represents an individual mouse and hypoglycemic cases are indicated with a red symbol. Bar graph represent median per group. Statistical differences were calculated with the Mann-Whitney *U* test with Holm-Bonferroni correction. Ns: p>0.05 and *: p<0.05. Data from two independent experiments. Asterisks above data points indicate significant differences between infected and non-infected mice, asterisks above a horizontal line show significant differences between genotype.

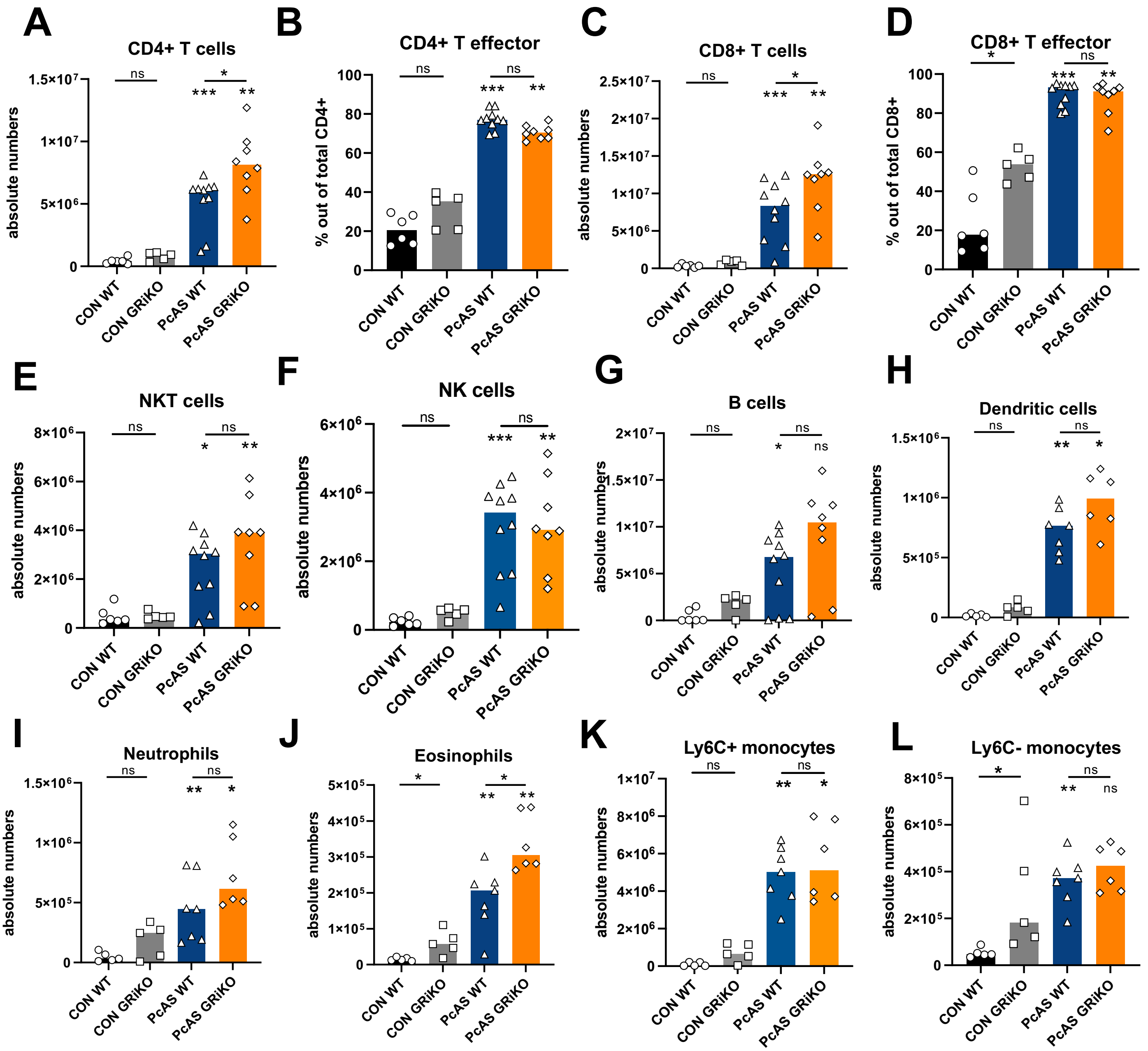

Fig. S4. Leukocyte populations in the liver are minimally affected by GR deletion. WT and GRiKO mice were infected with *Pc*AS parasites. At 10 dpi, liver was collected and flowcytometry was performed on isolated leukocytes. The absolute number of **(A)** $CD4^+$ T cells, **(C)** $CD8^+$ T cells, **(E)** NKT cells, **(F)** NK cells, **(G)** B cells, **(H)** dendritic cells, **(I)** neutrophils, **(J)** eosinophils, **(K)** $Ly6C^+$ monocytes and **(L)** $Ly6C^-$ monocytes were defined. The activation status of $CD4^+$ and $CD8^+$ were further explored based on CD44 and CD62L expression. The percentage of **(B)** effector (gated as $CD44^+$ $CD62L^-$) CD4+ T cells and **(D)** effector CD8+ T cells are shown. Gating strategies are shown in Appendix figure S12. Each symbol represents an individual mouse. Bar graph represent median per group. Data from two independent experiments. Statistical differences were calculated with the Mann-Whitney *U* test with Holm-Bonferroni correction. ns: p>0.05, *: p<0.05, **: p<0.01, ***: p<0.001. Asterisks above data points indicate significant differences between infected and non-infected mice, asterisks above a horizontal line show significant differences between genotype.

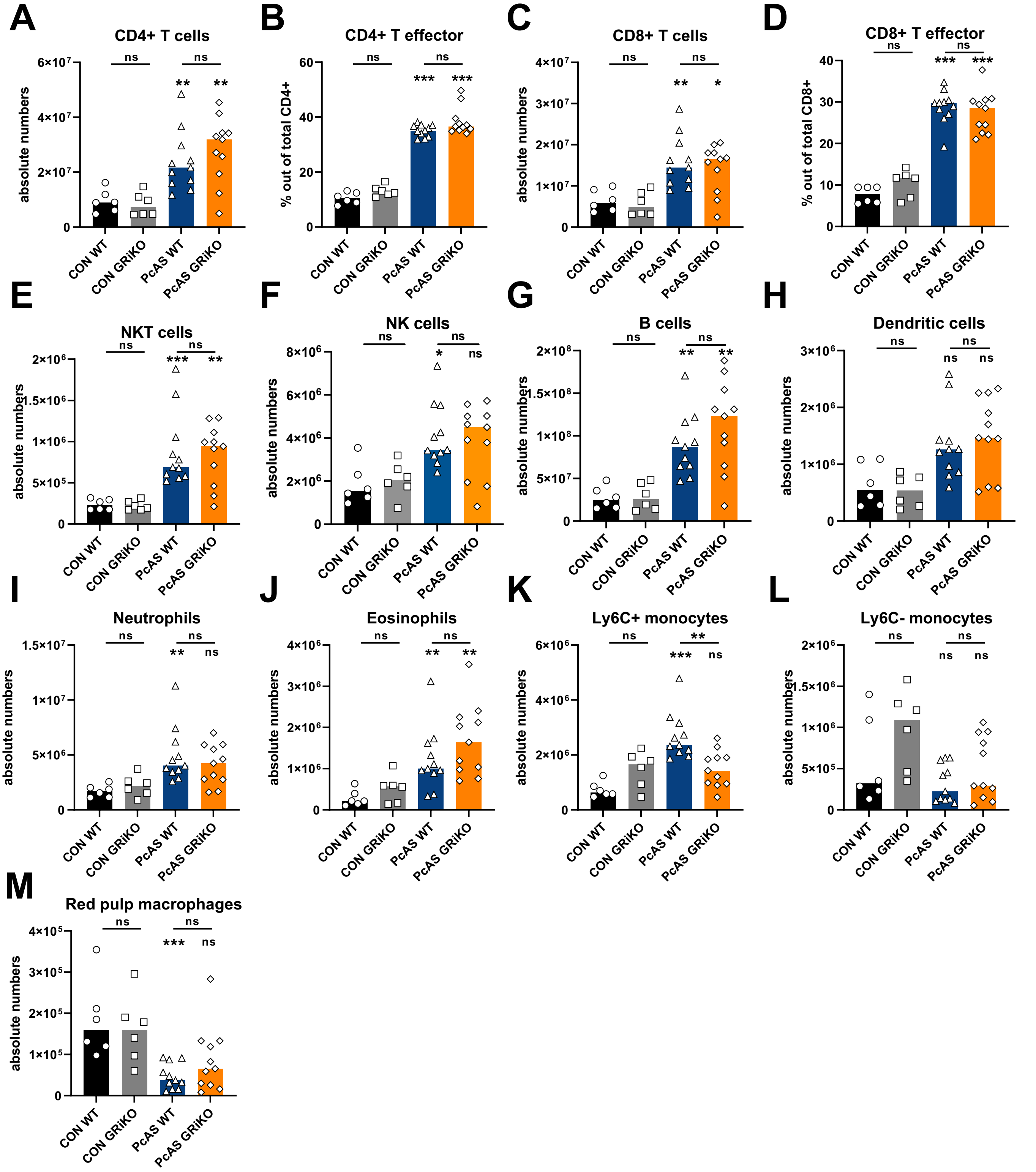

Fig. S5. Endogenous GCs minimally change the number of leukocytes in the spleen during infection. WT and GRiKO mice were infected with *Pc*AS parasites. At 10 dpi, spleen was collected and flowcytometry was performed on isolated leukocytes. The absolute number of **(A)** CD4$^+$ T cells, **(C)** CD8$^+$ T cells, **(E)** NKT cells, **(F)** NK cells, **(G)** B cells, **(H)** dendritic cells, **(I)** neutrophils, **(J)** eosinophils, **(K)** Ly6C$^+$ monocytes, **(L)** Ly6C$^-$ monocytes and **(M)** red pulp macrophages were defined. The activation status of CD4$^+$ and CD8$^+$ were further explored based on CD44 and CD62L expression. The percentage of **(B)** effector (gated as CD44$^+$ CD62L$^-$) CD4+ T cells and **(D)** effector CD8+ T cells are shown. Gating strategies are shown in Appendix figure S13. Each symbol represents an individual mouse. Bar graph represent median per group. Data from two independent experiments. Statistical differences were calculated with the Mann-Whitney *U* test with Holm-Bonferroni correction. ns: p>0.05, *: p<0.05, **: p<0.01, ***: p<0.001. Asterisks above data points indicate significant differences between infected and non-infected mice, asterisks above a horizontal line show significant differences between genotype.

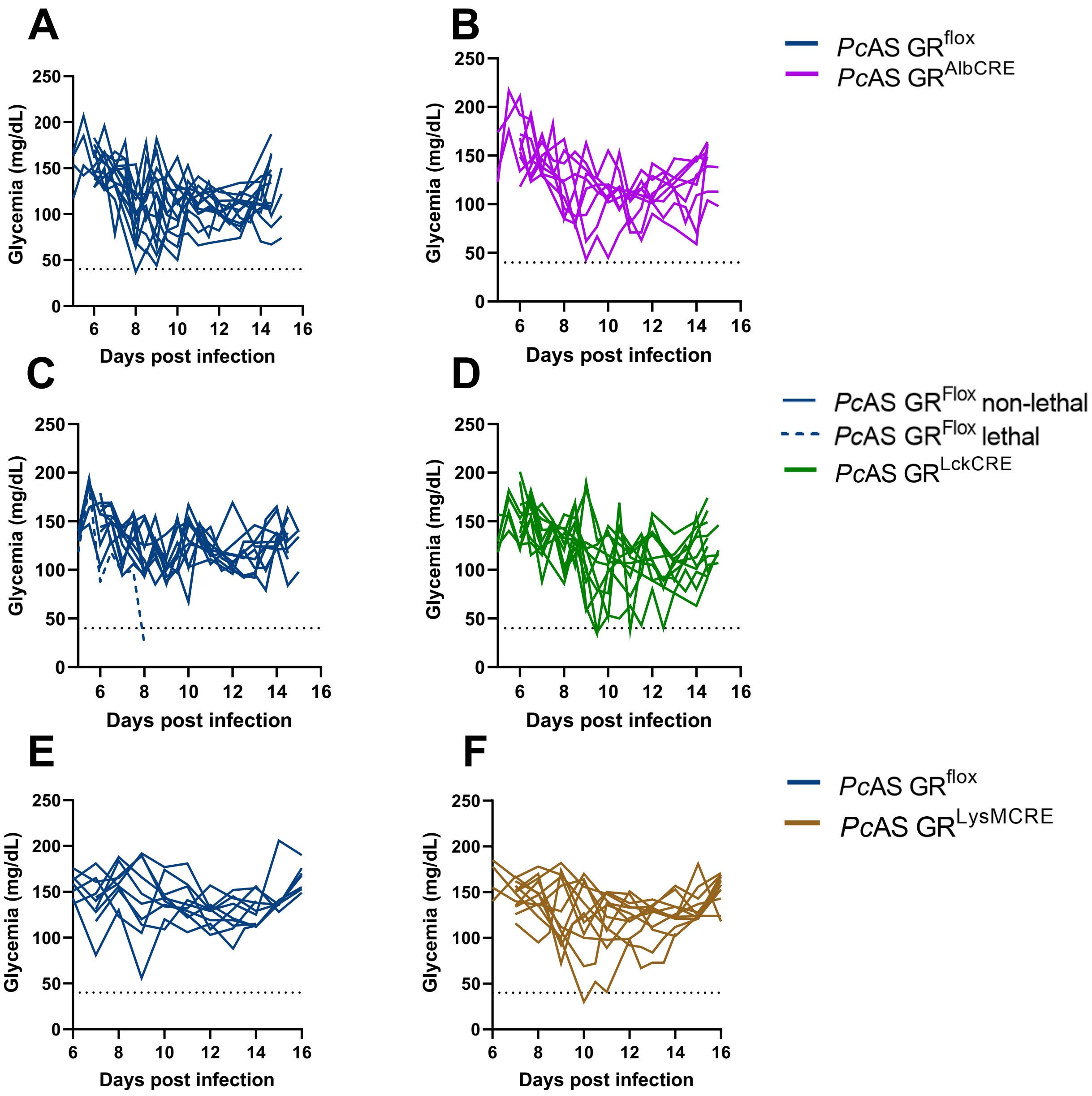

Fig. S6. Hepatocyte, T cell or myeloid cell specific GR deletion does not impact glycemia levels upon *Pc*AS infection. Hepatocyte (AlbCRE), T cell (LckCRE or myeloid (LysMCRE) specific GR KO mice were infected with *Pc*AS parasites. Infected mice were monitored for from 6 or 7 dpi onwards. Glycemia levels are shown for experiments performed with **(A-B)** AlbCRE mice, **(C-D)** LckCRE mice and **(E-F)** LysMCRE mice. Each line represents an individual mouse and dotted line at 40 mg/dL indicates the upper limit of severe hypoglycemia. Data from two or three individual experiments. The number of mice for graphs A-B: *Pc*AS GR^{Flox} : 9 mice and *Pc*AS GR^{AlbCRE}: 14 mice, for graphs C-D: *Pc*AS GR^{Flox} : 11 mice and *Pc*AS GR^{LckCRE}: 11 mice, and for graphs E-F: *Pc*AS GR^{Flox} : 10 mice and *Pc*AS GR^{LysMCRE}: 12 mice.

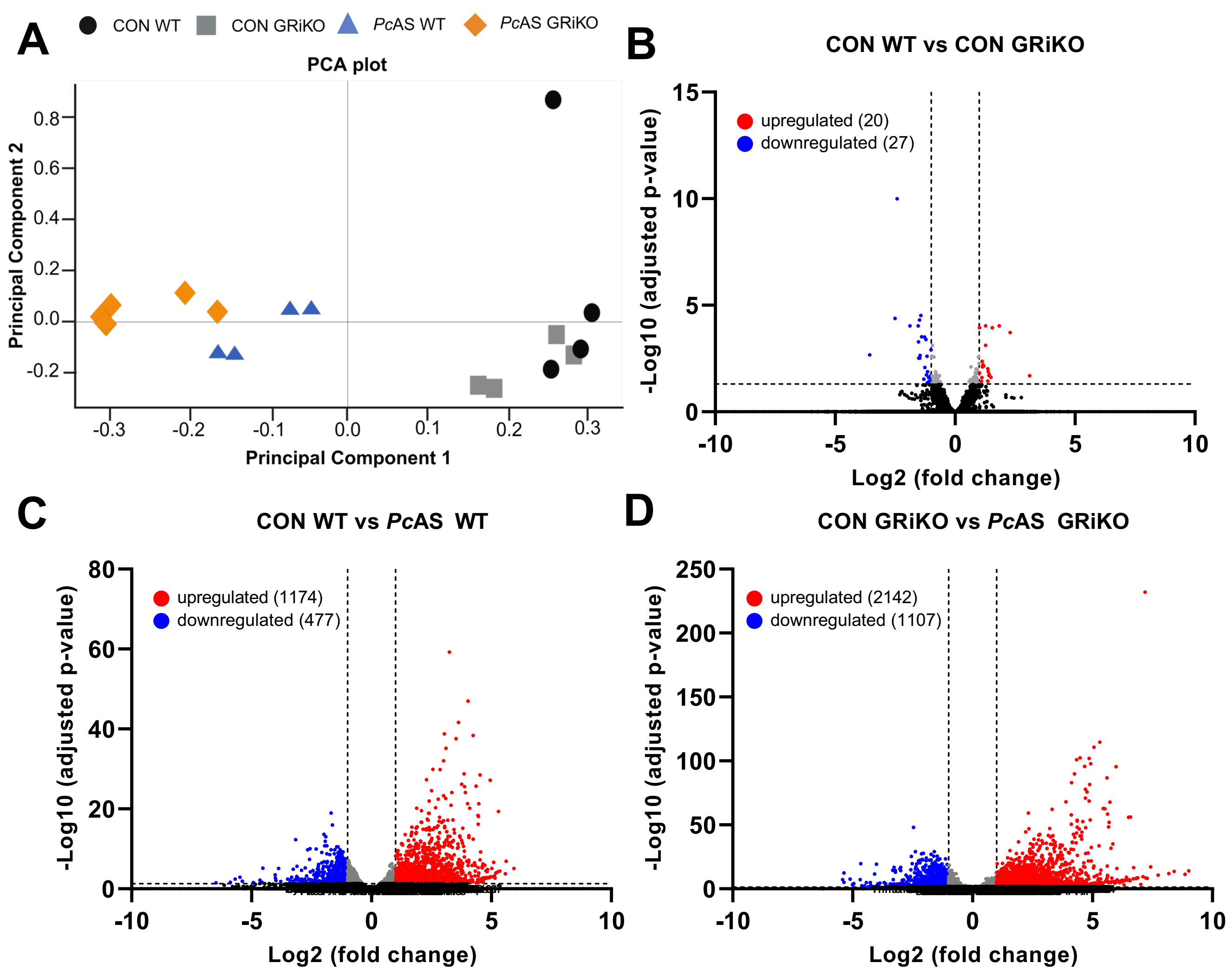

Fig. S7. RNAseq reveals transcriptional changes in liver upon *Pc*AS infection. WT and GRiKO mice were infected with 10^4 *Pc*AS parasites and RNAseq was performed on liver tissue collected at 10 dpi. **(A)** Principal component analysis was performed. Differential gene analysis was performed with DESeq2 for 4 different comparisons i.e. **(B)** CON WT vs CON GRiKO, **(C)** CON WT vs *Pc*AS WT and **(D)** CON GRiKO vs *Pc*AS GRiKO. A volcano plot was made to examine the differentially expressed genes (DEGs). Data from one individual experiment.

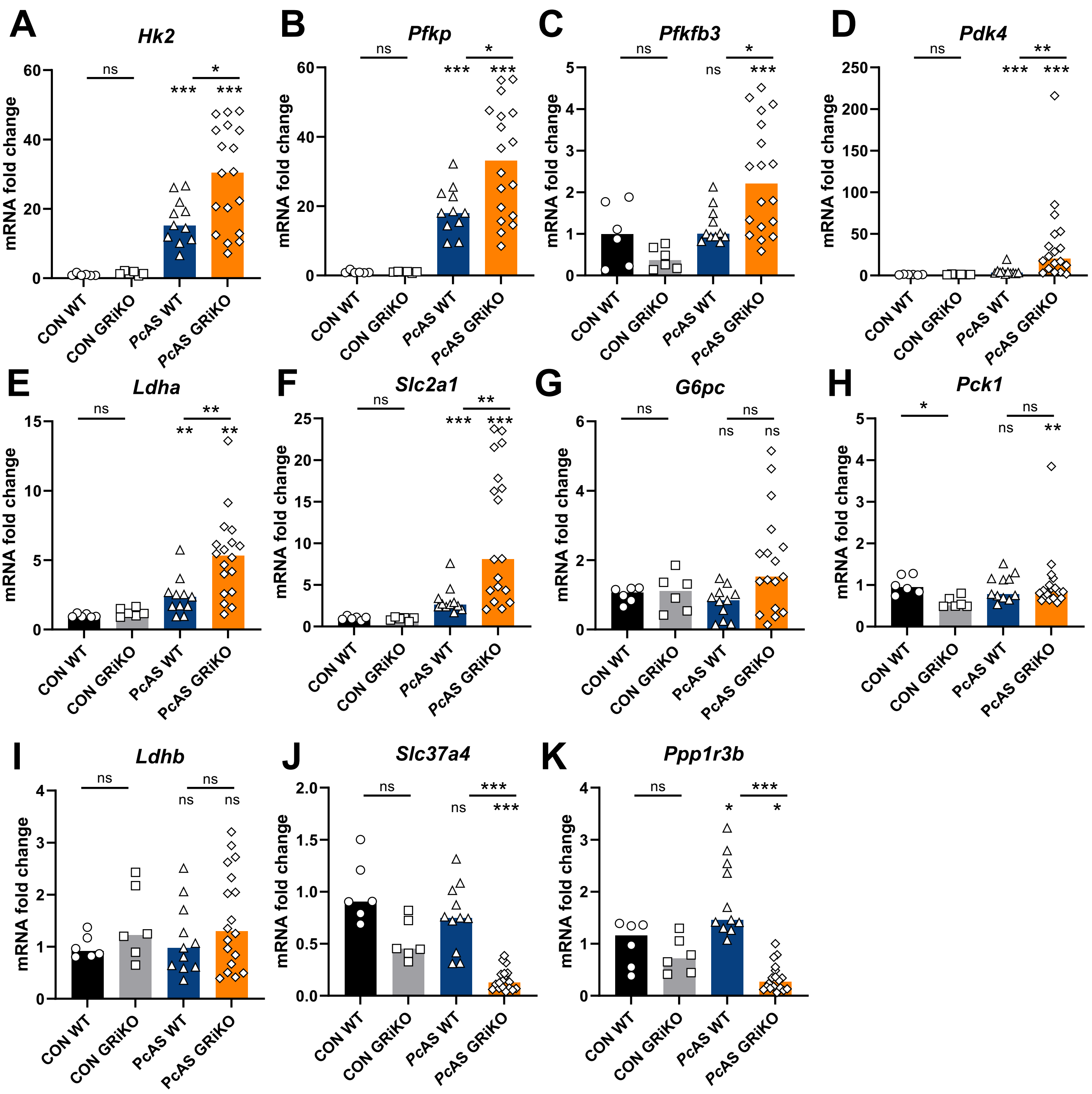

Fig. S8. GR signalling inhibits glycolytic gene expression and supports minimal gluconeogenic gene expression in liver during infection. WT and GRiKO mice were infected with *Pc*AS parasites and livers were collected at 10 dpi. qPCR was performed to determine mRNA expression of **(A)** *Hk2*, **(B)** *Pfkp*, **(C)** *Pfkfb3*, **(D)** *Pdk4*, **(E)** *Ldha,* **(F)** *Slc2a1***,** **(G)** *G6pc*, **(H)** *Pck1*, **(I)** *Ldhb*, **(J)** *Slc37a4* and **(K)** *Ppp1r3b*. Statistical differences were calculated with the Mann-Whitney *U* test with Holm-Bonferroni correction. ns: $p>0.05$, *: $p<0.05$, **: $p<0.01$, ***: $p<0.001$. Asterisks above data points indicate significant differences between infected and non-infected mice, asterisks above a horizontal line show significant differences between genotype. Each symbol represents an individual mouse and bar graph represent median per group. Data from two independent experiments.

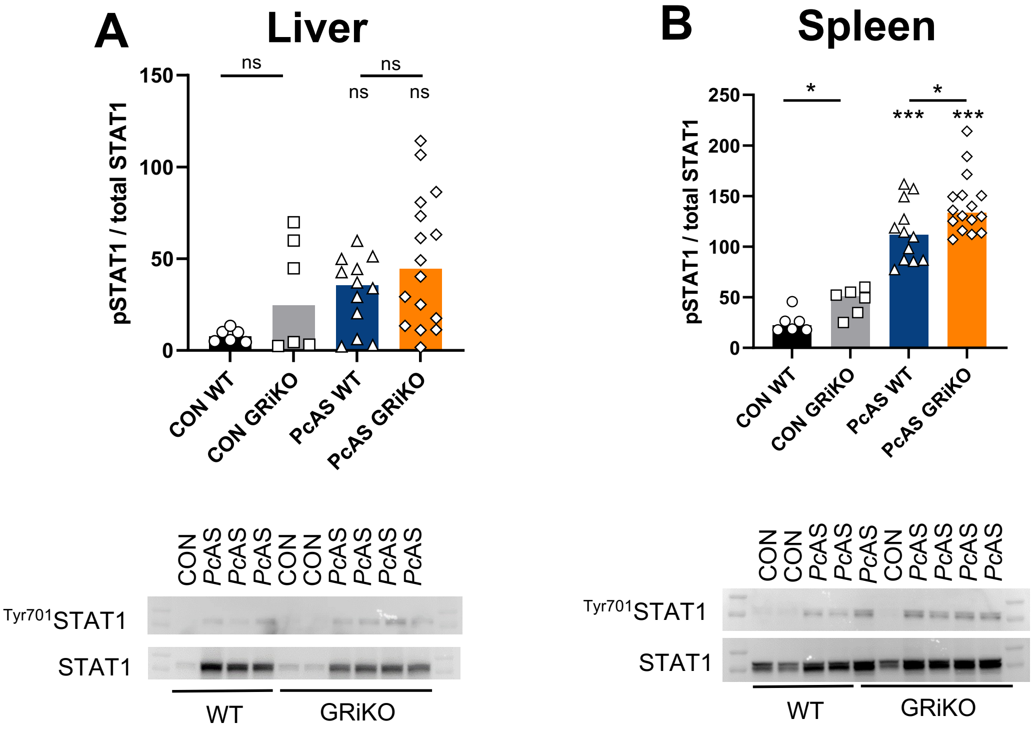

Fig. S9. Endogenous GCs interfere minimally with STAT1 in spleen, but not in liver upon infection. WT and GRiKO mice were infected with *Pc*AS parasites. At 10 dpi, **(A)** liver and **(B)** spleen tissue was collected and phosphorylation (on tyrosine residue 701) status of STAT1 was assessed in liver and spleen protein extracts. Representative blots are shown together with the semiquantitative determination of band intensities. Statistical differences were calculated with the Mann-Whitney *U* test with Holm-Bonferroni correction. ns: $p>0.05$, *: $p<0.05$, ***: $p<0.001$. Asterisks above data points indicate significant differences between infected and non-infected mice, asterisks above a horizontal line show significant differences between genotype. Each symbol represents an individual mouse and bar graph represent median per group. Data from two independent experiments.

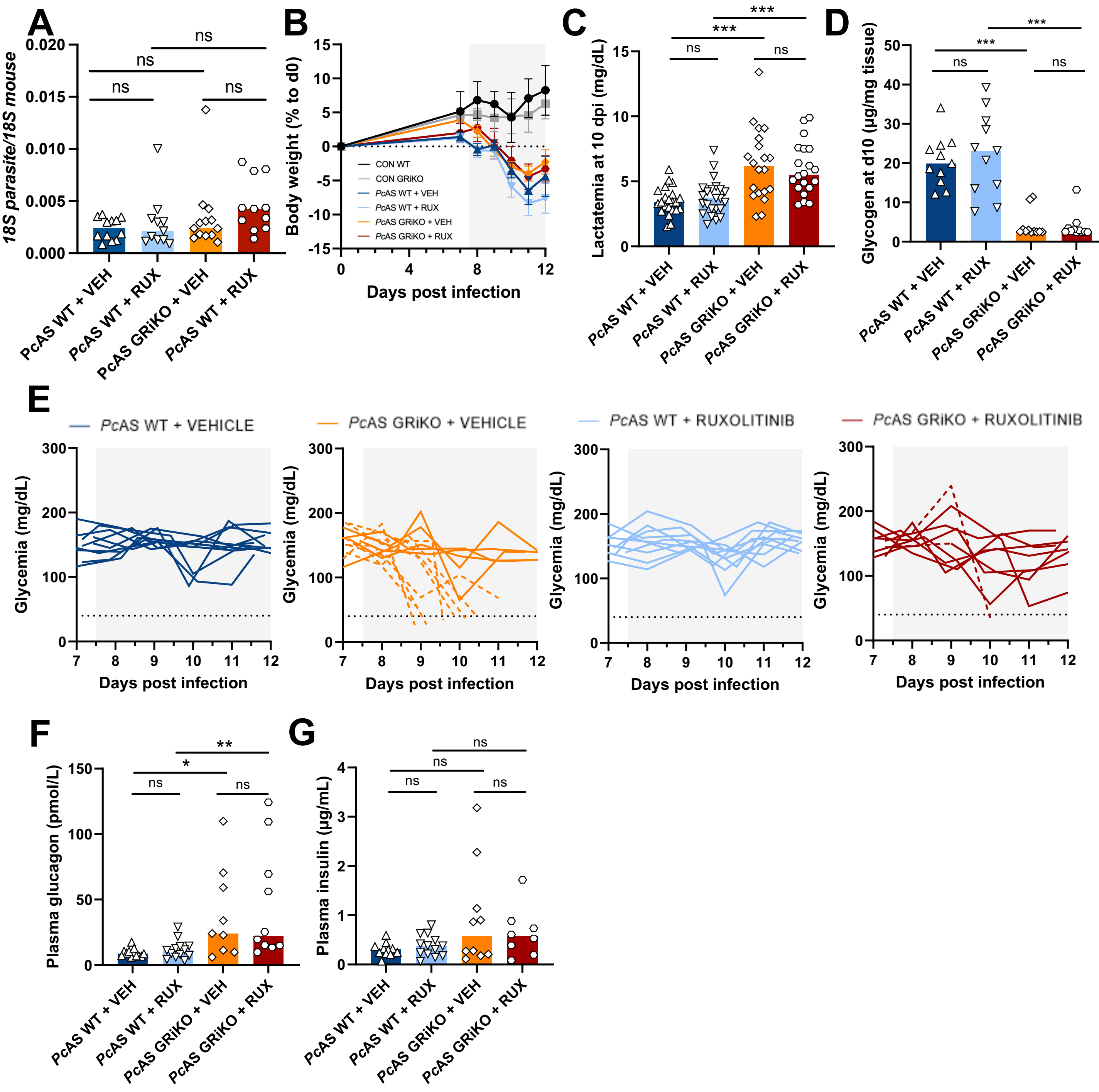

Fig. S10. Ruxolitinib treatment of infected GRiKO mice prevents lethal hypoglycemia after *Pc*AS infection, but does not affect body weight loss, hyperlactatemia, hepatic glycogen levels, glucagon or insulin levels. WT and GRiKO mice were infected with *Pc*AS parasites. From day 7 p.i. mice were treated with Ruxolitinib (RUX, 90 mg/kg, 2x/day, p.o.) or vehicle (VEH). **(A)** Parasite load in the liver was determined at 10 dpi by measuring parasite and mouse-specific 18S by qPCR. **(B)** Body weight loss was measured throughout the infection. At 10 dpi, **(C)** lactatemia was measured and **(D)** hepatic glycogen stores were determined. **(E)** Glycemia levels were determined daily from 7 dpi (dotted lines: lethal cases, black horizontal lines: threshold for severe hypoglycemia at 40 mg/dL). At 10 dpi, **(F)** plasma glucagon levels and **(G)** plasma insulin levels were assessed. For B: mean and SEM of each group is shown. For E: each line presents an individual mouse. For A, C-D and F-G: Bar graph represent median per group and each datapoint represents an individual mouse. Data from two to four separate experiments. Statistical differences were calculated with the Mann-Whitney *U* test with Holm-Bonferroni correction. ns: p>0.05, * p<0.01, ** p<0.001 and *** p<0.0001. The number of mice for graphs B: CON WT: 3 mice, CON GRiKO: 3 mice, *Pc*AS WT + VEHICLE: 10 mice, *Pc*AS WT + RUXOLITINIB: 10 mice, *Pc*AS GRiKO + VEHICLE: 13 mice, and *Pc*AS GRiKO + RUXOLITINIB: 9 mice.

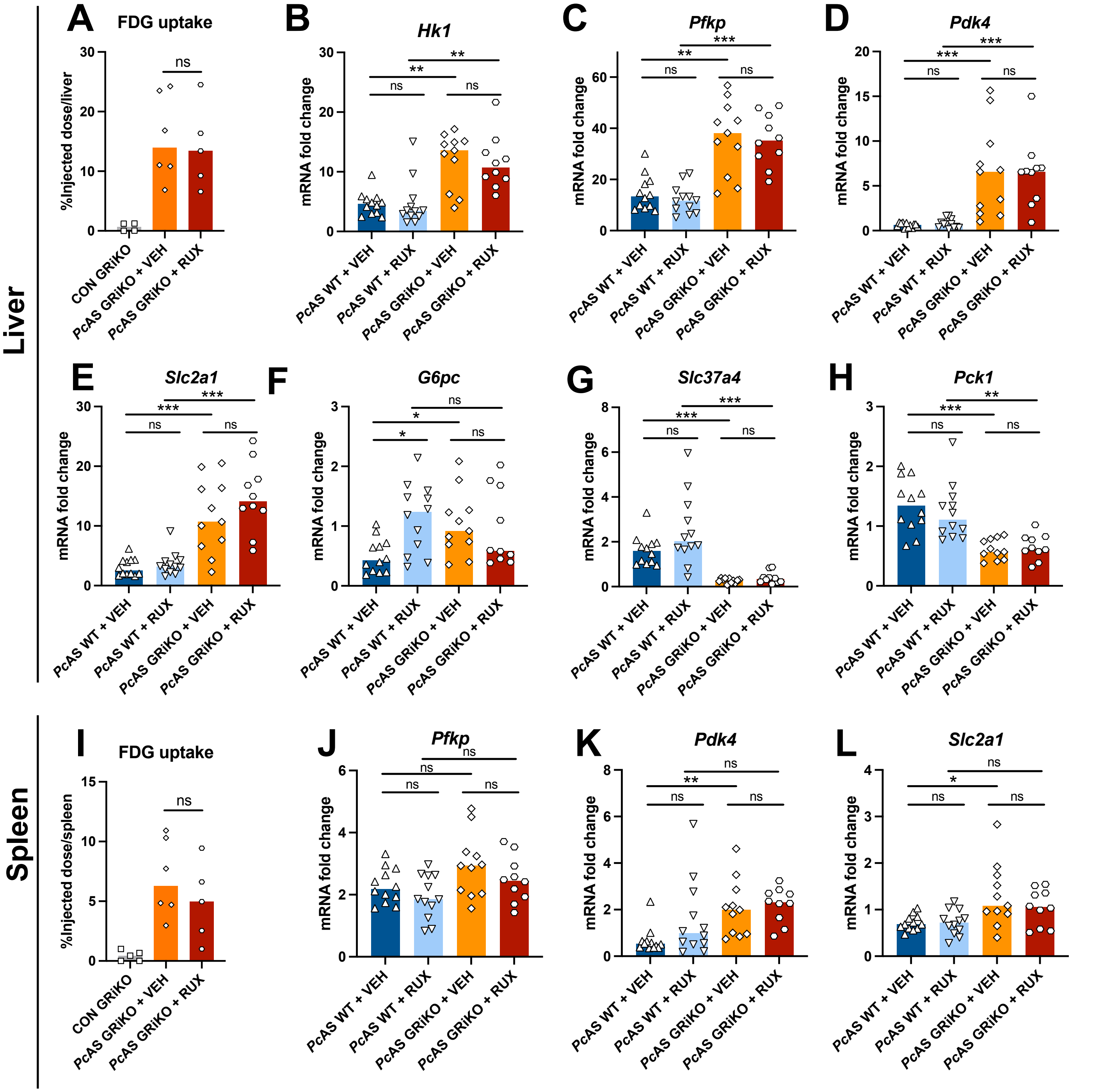

Fig. S11. Ruxolitinib treatment has no effect on hepatic and splenic FDG uptake and only minorly changes glycolytic and gluconeogenic gene expression levels. WT and GRiKO mice were infected with 10^4 *Pc*AS parasites. Mice were treated with 90 mg/kg ruxolitinib (RUX) or vehicle (VEH) twice/day via oral gavage, from 7 dpi onwards. Mice were monitored from 6 onwards. At 10 dpi, [^{18}F]-FDG was administered and uptake in the **(A)** liver and **(I)** the spleen was measured with a gamma counter. Gene expression levels of **(B)** *Hk1*, **(C)** *Pfkp*, **(D)** *Pdk4*, **(E)** *Slc2a1*, **(F)** *G6pc*, **(G)** *Slc37a4* and **(H)** *Pck1* were detected in the liver with qPCR. In the spleen, **(J)** *Pfkp*, **(K)** *Pdk4* and **(L)** *Slc2a1* mRNA expression levels were measured. All gene expression levels were normalized towards expression levels of uninfected control mice and mouse-specific *18S* expression. Bar graph represent median per group and each datapoint represents an individual mouse. Statistical differences were calculated with the Mann-Whitney *U* test with Holm-Bonferroni correction. ns: p>0.05, *: p<0.05, **: p<0.01, ***: p<0.001. Data from two independent experiments.

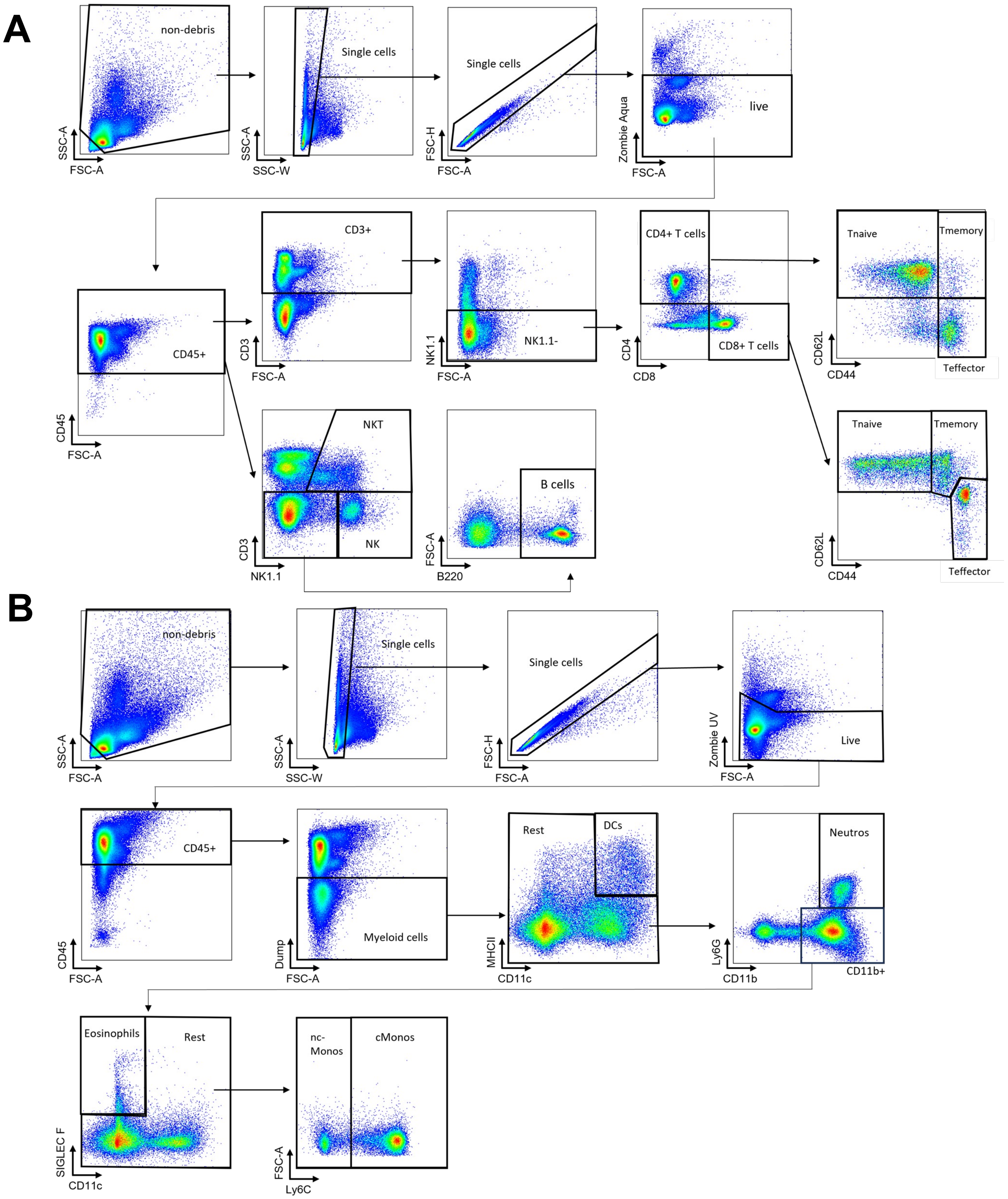

Fig. S12. Gating strategies to identify leukocytes in the liver. Leukocytes were isolated from individual livers and analysed via flow cytometry. Debris, doublets and dead cells were first excluded. Leukocytes were identified by positive staining for CD45. (A) Lymphoid cell populations were identified as followed: CD4+ T cells (CD4$^+$ CD3$^+$ NK1.1$^-$), CD8+ T cells (CD8$^+$ CD3$^+$ NK1.1$^-$), NKT cells (CD3$^+$ NK1.1$^+$), NK cells (CD3$^-$ NK1.1$^+$) and B cells (CD3$^-$ NK1.1$^-$ B220$^+$). The activation status of the T cells were analysed with the markers CD44 and CD62L as followed: T naïve (CD44$^-$CD62L$^+$), T central memory (CD44$^+$CD62L$^+$) and T effector (CD44$^+$CD62L$^-$) cells. (B) To identify myeloid cell, cells were first gated for negative staining for CD3, CD19 and NK1.1 (dump). Thereafter, dendritic cells (DCs) were identified as CD11c$^+$MHCII$^+$, neutrophils (neutros) as CD11b$^+$Ly6G$^+$, eosinophils (eosinos) as CD11c$^-$ siglecF$^+$, non-classical monocytes (ncMonos) as CD11b$^+$Ly6c$^-$ and classical monocytes (cMonos) as CD11b$^+$Ly6c$^+$.

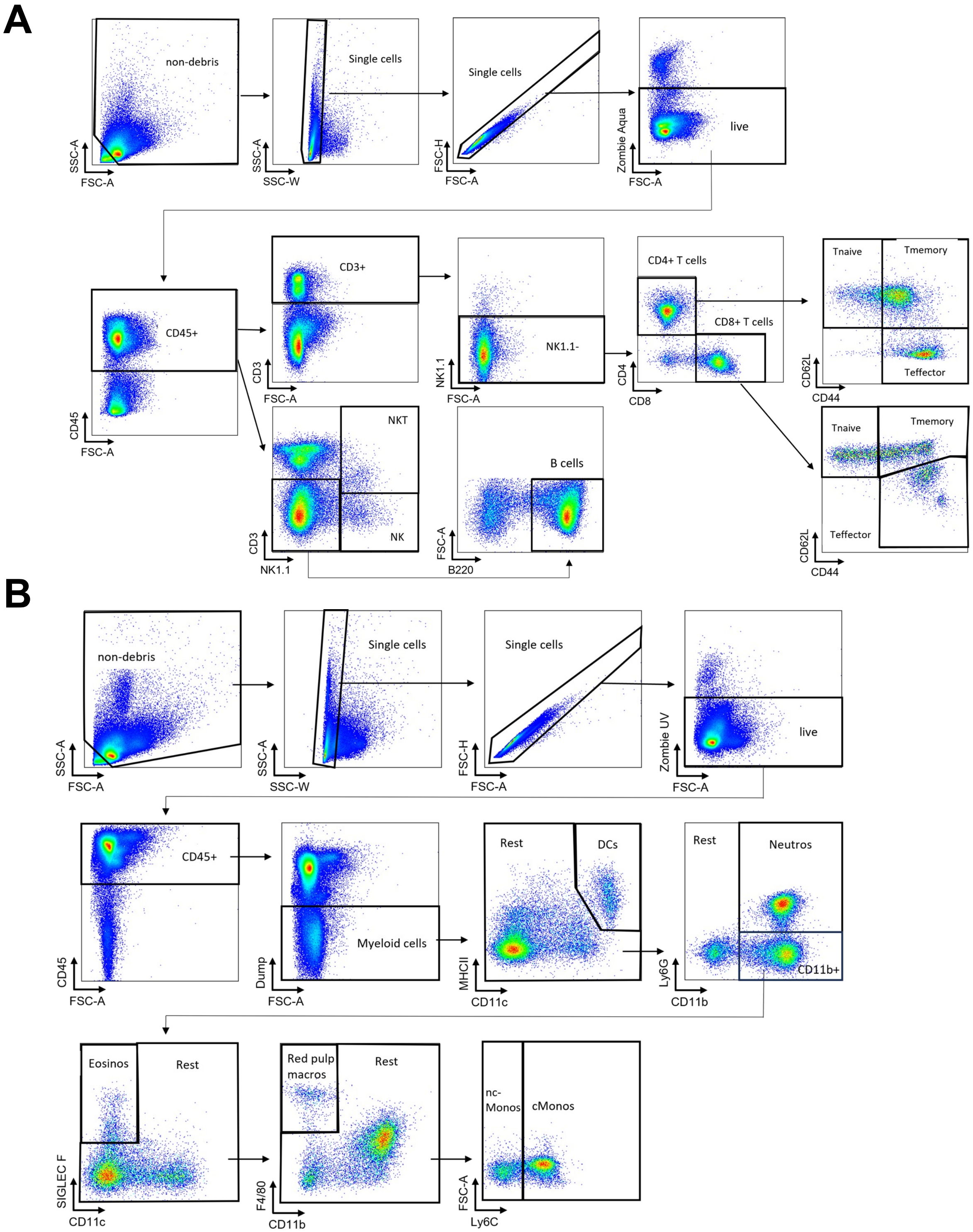

Fig. S13. Gating strategies to identify leukocytes in the spleen. Splenocytes were isolated from individual spleens and analysed via flow cytometry. Debris, doublets and dead cells were first excluded. Leukocytes were identified by positive staining for CD45. (A) Lymphoid cell populations were identified as followed: CD4^{+} T cells (CD4^{+} CD3^{+} NK1.1^{-}), CD8+ T cells (CD8^{+} CD3^{+} NK1.1^{-}), NKT cells (CD3^{+} NK1.1^{+}), NK cells (CD3^{-} NK1.1^{+}) and B cells (CD3^{-} NK1.1^{-} B220^{+}). The activation status of the T cells were analysed with the markers CD44 and CD62L as followed: T naïve (CD44-CD62L^{+}), T central memory (CD44^{+}CD62L^{+}) and T effector (CD44^{+}CD62L^{-}) cells. (B) To identify myeloid cell, cells were first gated for negative staining for CD3, CD19 and NK1.1 (dump). Thereafter, dendritic cells (DCs) were identified as CD11c^{+}MHCII^{+}, neutrophils (neutros) as CD11b^{+}Ly6G^{+}, eosinophils (eosinos) as CD11c^{-} siglecF^{+}, red pulp macrophages (macros) as CD11b^{-}F4/80^{+}, non-classical monocytes (ncMonos) as CD11b^{+}Ly6c^{-} and classical monocytes (cMonos) as CD11b^{+}Ly6c^{+}.

Table S1. Primers for GR genotyping.

Primer	Sequence
GR1 (GR)	5’-GGC ATG CAC ATT ACT GGC CTT CT-3’
GR4 (Grflox)	5’-GTG TAG CAG CCA GCT TAC AGG A-3’
GR8 (GR wildtype)	5’-CCT TCT CAT TCC ATG TCA GCA TGT-3’
CRE1	5’-CGG TCG ATG CAA CGA GTG ATG AGG-3’
CRE2	5’-CCA GAG ACG GAA ATC CAT CGC TCG-3’

Table S2. Fluorescently labelled extracellular antibodies for flowcytometry.

Antigen	Clone	Fluorophore	Catalogue number	Company
Lymphoid panel				
CD3	145-2C11	FITC	11-0031-81	eBioscience
CD8	53-6.7	PerCP-Cy5.5	45-0081-82	eBioscience
CD44	IM7	PE	103007	Biolegend
NK1.1	PK136	PE-Cy7	25-5941-81	eBioscience
CD4	RM4-5	APC e780	47-0042-80	eBioscience
B220	RA3-6B2	BV786	563894	BD
CD62L	MEL14	BV711	104445	Biolegend
CD45	30-F11	BUV395	564279	BD
Myeloid panel				
CD45	30-F11	FITC	103107	Biolegend
CD11c	N418	PE-CY7	117318	Biolegend
F4/80	BM8	PE	12-4801-82	eBioscience
Siglec F	E50-2440	PE-CF594	562757	BD
Ly6G	1A8	AF700	561236	BD
Ly6C	AL-21	APC-Cy7	560596	BD
CD11b	TM-25	eFluor 450	48-0112-80	eBioscience
MHCII	M5/114	Horizon v500	562366	BD
CD3	17A2	BV650	100229	Biolegend
CD19	6D5	BV650	115541	Biolegend
NK1.1	PK136	BV650	108736	Biolegend

Table S3. histopathological scoring system for H&E-stained liver slides

Histopathological changes	Histopathological grading			
	**0**	**1**	**2**	**3**
**Inflammation**	No	Mild	Moderate	Severe
**Sinusoid congestion/dilatation**	No	Mild	Moderate	Severe
**Hemozoin deposition**	No	Mild	Moderate	Severe
**Necrosis/apoptosis**	No	Mild	Moderate	Severe

Table S4. Primers for RT-qPCR

Gene	Full protein name	Exon	Dye	Catalogue number
18S	*18S ribosomal RNA - mouse*	1-1	FAM™	Hs.PT.39a.22214856.g
18S	*18S ribosomal RNA – Pc*AS parasite	/	FAM™	Custom design
G6pc	*Glucose-6-phosphatase alpha*	4-5	FAM™	Mm.PT.58.11964858
Hk2	*Hexokinase 2*	3-4	FAM™	Mm.PT.58.32698746
Ldha	*Lactate dehydrogenase A*	3-4	FAM™	Mm.PT.58.29860774
Ldhb	*Lactate dehydrogenase B*	2-3	FAM™	Mm.PT.58.9685691
Pck1	*Phosphoenolpyruvate Carboxykinase*	3-4	FAM™	Mm.PT.58.11992693
Pdk4	*Pyruvate dehydrogenase kinase 4*	6-8	FAM™	Mm.PT.58.9453460
Pfkfb3	*Phosphofructokinase/ fructosebiphosphatase 3*	3-4	FAM™	Mm.PT.58.29645117
Pfkp	*Phosphofructokinase, platelet*	10-12	FAM™	Mm.PT.58.23785960
Ppp1r3b	*Protein phosphatase 1 regulatory subunit 3B*	1-2	FAM™	Mm.PT.58.6871404
Slc2a1	*Solute carrier family 2 member 1*	2-3	FAM™	Mm.PT.58.7590689
Slc37a4	*Solute carrier family 37 member 4*	1-2	FAM™	Mm.PT.58.31385792
Slc16a4	*Solute carrier family 16 member 4*	6-7	FAM™	Mm.PT.58.43799834
Il1β	*Interleukin-1 beta*	3-4	FAM™	Mm.PT.58.41616450
Il6	*Interleukin-6*	4-5	FAM™	Mm.PT.58.10005566
Il10	*Interleukin-10*	3-5	FAM™	Mm.PT.58.13531087
Tnf	*Tumor necrosis factor*	2-4	FAM™	Mm.PT.58.12575861
Ifnα	*Interferon alpha*	1-1	FAM™	Mm.PT.58.43426930
Ifnγ	*Interferon gamma*	1-2	FAM™	Mm.PT.58.41769240

Table S5. Primary anti-mouse antibodies for Western blot analysis

Antigen	Dilution	Blocking buffer	Catalogue number	Company
GR	1:1000	5% NFDM	D8H2	Cell Signaling Technologies
Beta-actin	1:2000	5% BSA	4967	Cell Signaling Technologies
HRP-tubulin	1:5000	5% BSA	BT7R	ThermoFisher Scientific
Vinculin	1:2000	5% NFDM	E1E9V	Cell Signaling Technologies
STAT3	1:2000	5% BSA	12640	Cell Signaling Technologies
STAT1	1:2000	5% BSA	9172	Cell Signaling Technologies
pSTAT3	1:1000	5% BSA	9145	Cell Signaling Technologies
pSTAT1	1:1000	5% BSA	9167	Cell Signaling Technologies
