# Supplementary figures and images for: JAK/STAT inhibition protects glucocorticoid receptor knockout mice from lethal malaria-induced hypoglycemia and hyperinflammation
Source: EMBO Mol Med. 2025 Jul 23;17(8):2040–70. doi: 10.1038/s44321-025-00264-w (PMC12340059; doi:10.1038/s44321-025-00264-w)

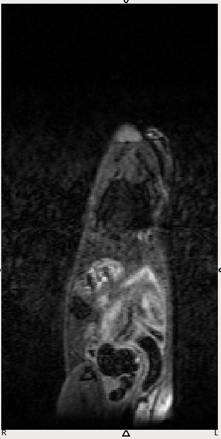

Supplement: Supplementary file 6 — Source data Fig. 2 [file 44321_2025_264_MOESM6_ESM.zip › Figure 2/2A/2A_CON WT_MRI.tif]

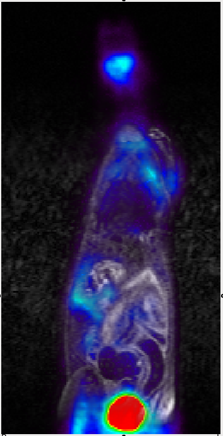

Supplement: Supplementary file 6 — Source data Fig. 2 [file 44321_2025_264_MOESM6_ESM.zip › Figure 2/2A/2A_CON WT_PET-MRI.tif]

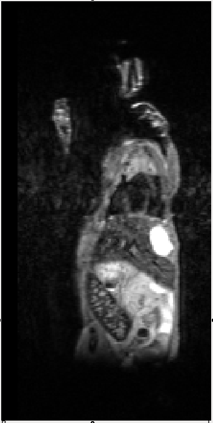

Supplement: Supplementary file 6 — Source data Fig. 2 [file 44321_2025_264_MOESM6_ESM.zip › Figure 2/2A/2A_PcAS GRiKO_MRI.tif]

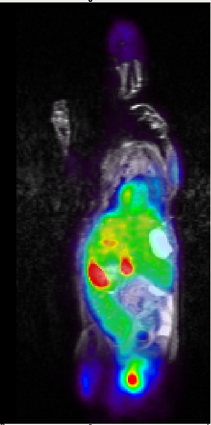

Supplement: Supplementary file 6 — Source data Fig. 2 [file 44321_2025_264_MOESM6_ESM.zip › Figure 2/2A/2A_PcAS GRiKO_PET-MRI.tif]

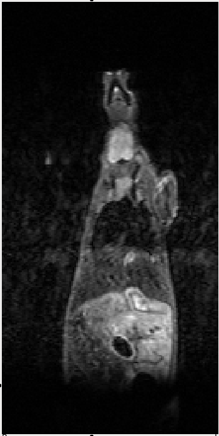

Supplement: Supplementary file 6 — Source data Fig. 2 [file 44321_2025_264_MOESM6_ESM.zip › Figure 2/2A/2A_PcAS WT_MRI.tif]

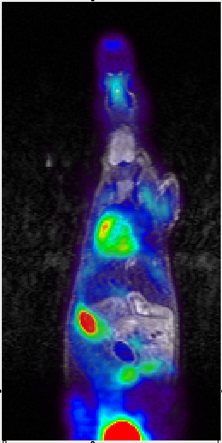

Supplement: Supplementary file 6 — Source data Fig. 2 [file 44321_2025_264_MOESM6_ESM.zip › Figure 2/2A/2A_PcAS WT_PET-MRI.tif]

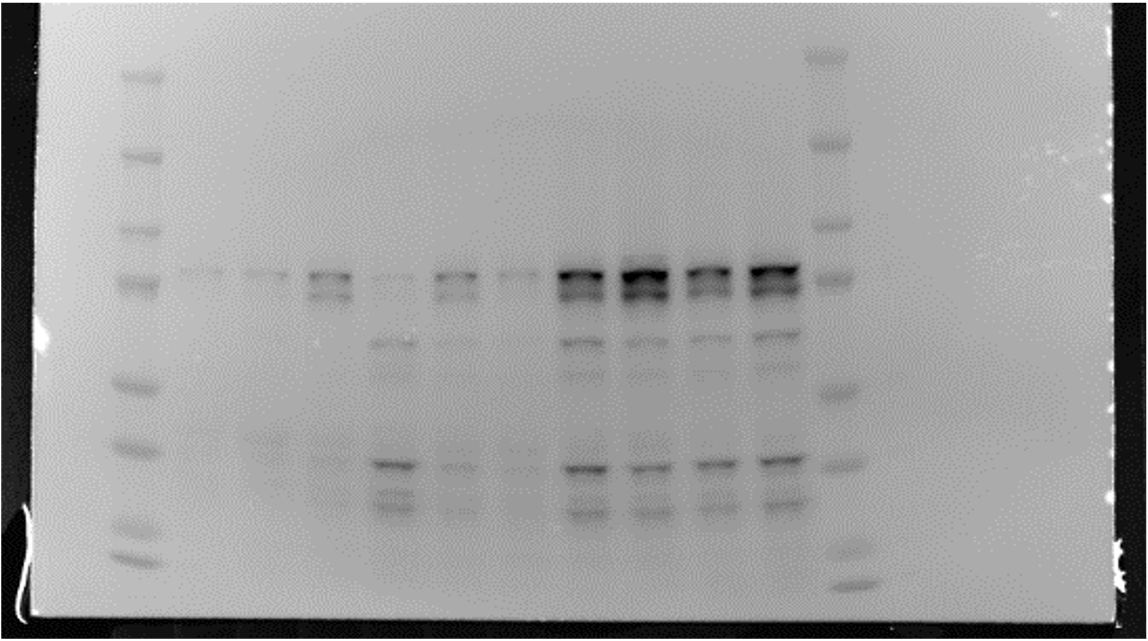

Supplement: Supplementary file 9 — Source data Fig. 5 [file 44321_2025_264_MOESM9_ESM.zip › Figure 5/5E/5E_pSTAT3_blot1.png]

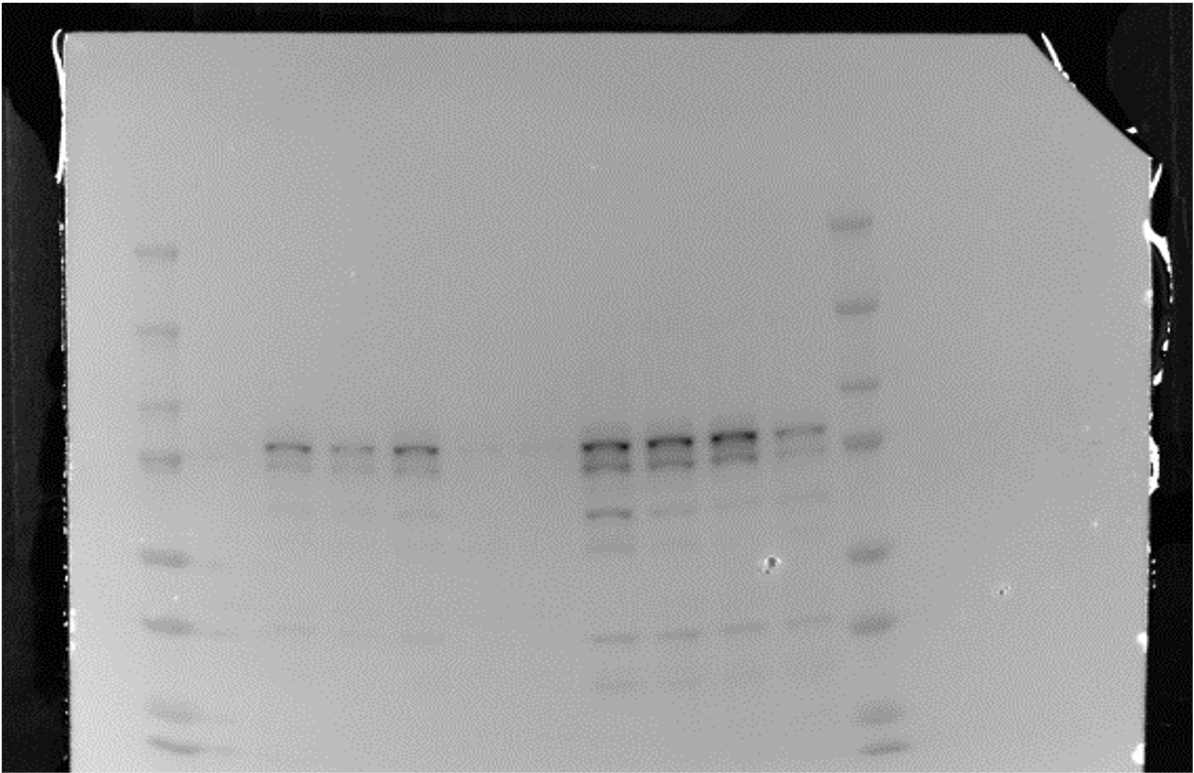

Supplement: Supplementary file 9 — Source data Fig. 5 [file 44321_2025_264_MOESM9_ESM.zip › Figure 5/5E/5E_pSTAT3_blot2.png]

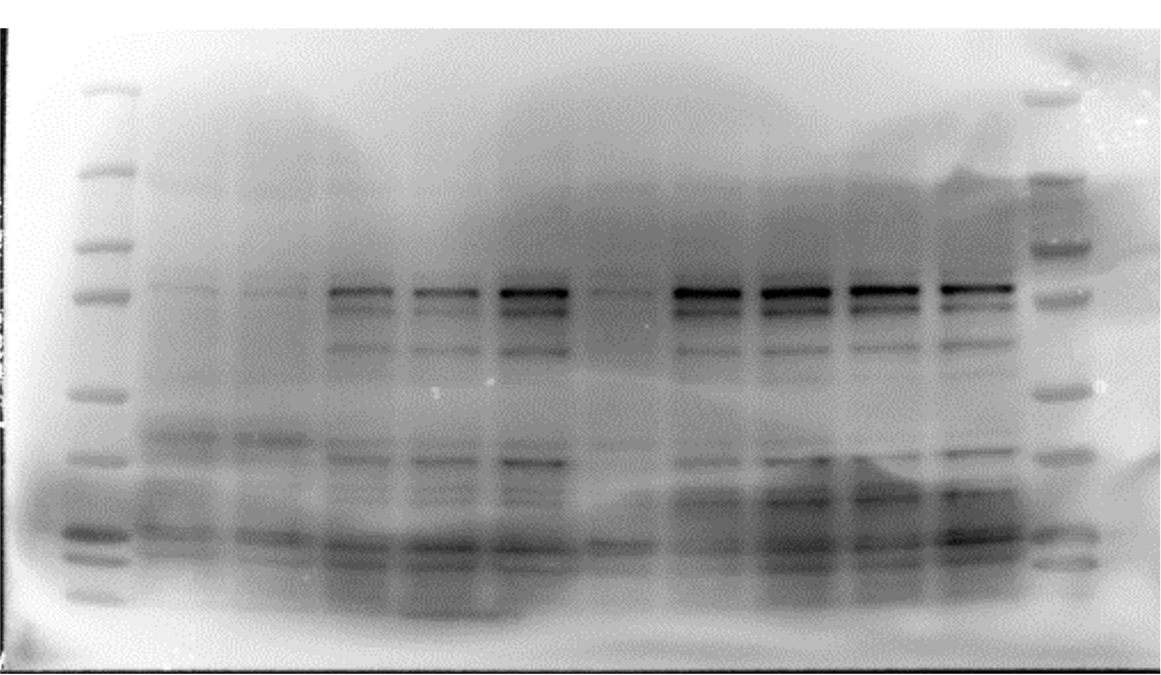

Supplement: Supplementary file 9 — Source data Fig. 5 [file 44321_2025_264_MOESM9_ESM.zip › Figure 5/5E/5E_pSTAT3_blot3.png]

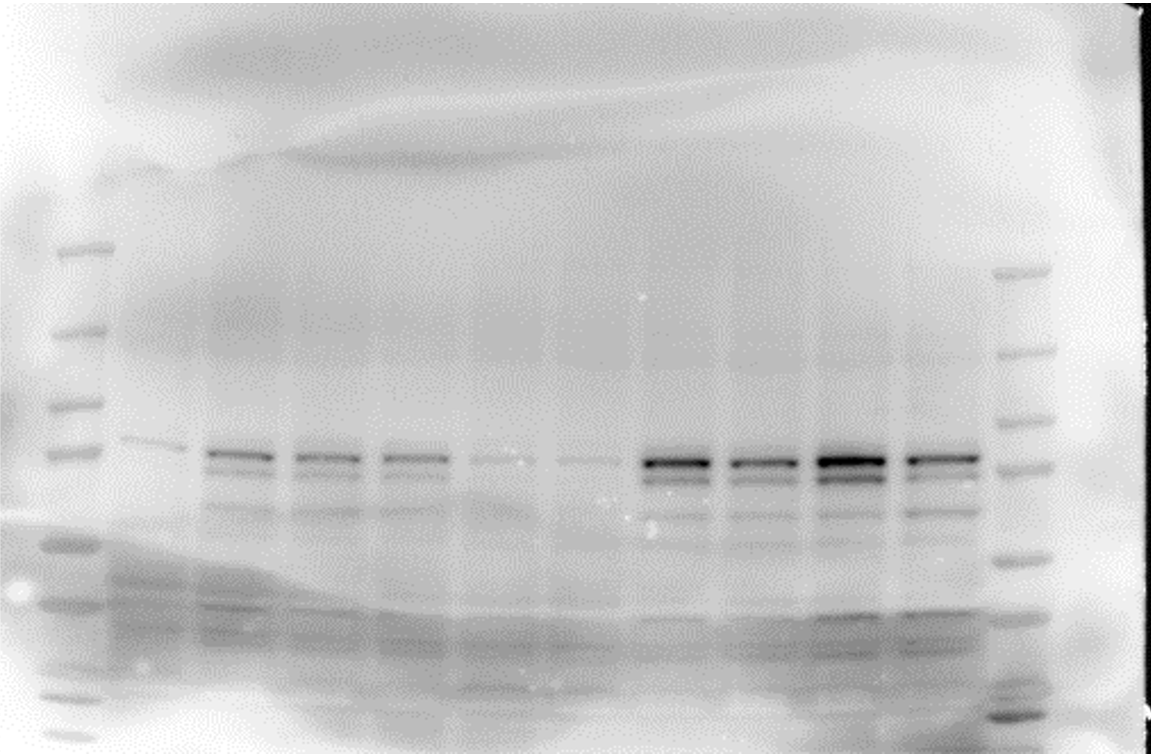

Supplement: Supplementary file 9 — Source data Fig. 5 [file 44321_2025_264_MOESM9_ESM.zip › Figure 5/5E/5E_pSTAT3_blot4.png]

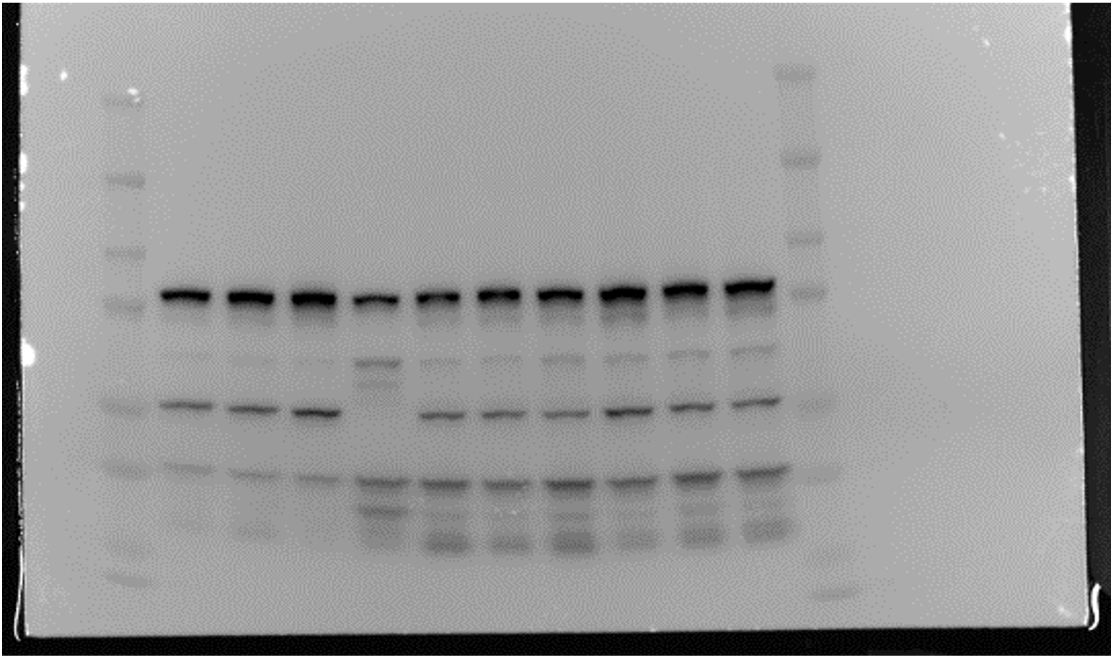

Supplement: Supplementary file 9 — Source data Fig. 5 [file 44321_2025_264_MOESM9_ESM.zip › Figure 5/5E/5E_STAT3_blot1.png]

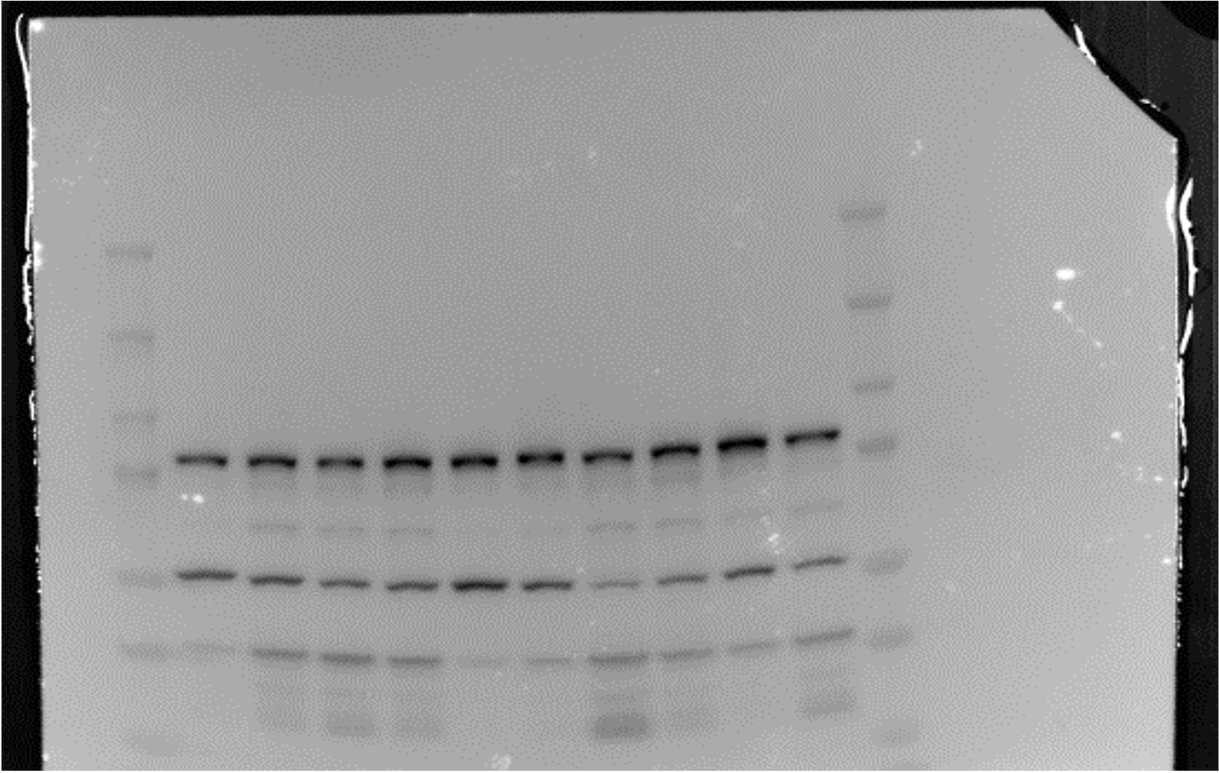

Supplement: Supplementary file 9 — Source data Fig. 5 [file 44321_2025_264_MOESM9_ESM.zip › Figure 5/5E/5E_STAT3_blot2.png]

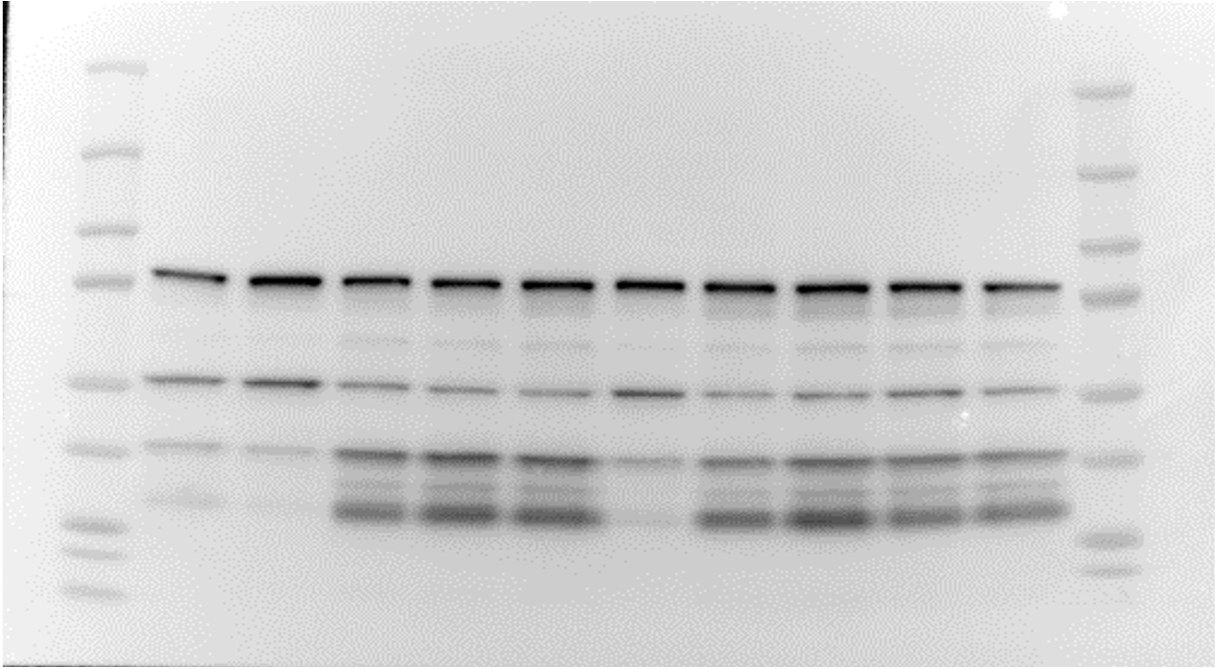

Supplement: Supplementary file 9 — Source data Fig. 5 [file 44321_2025_264_MOESM9_ESM.zip › Figure 5/5E/5E_STAT3_blot3.png]

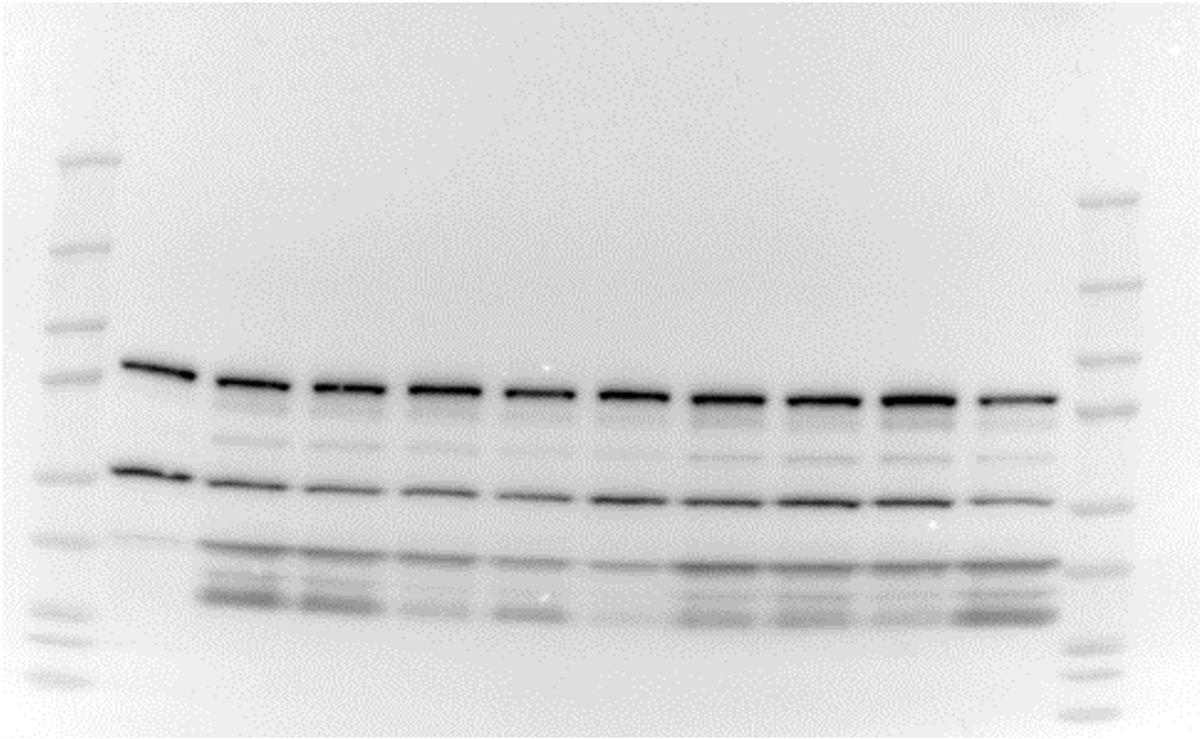

Supplement: Supplementary file 9 — Source data Fig. 5 [file 44321_2025_264_MOESM9_ESM.zip › Figure 5/5E/5E_STAT3_blot4.png]

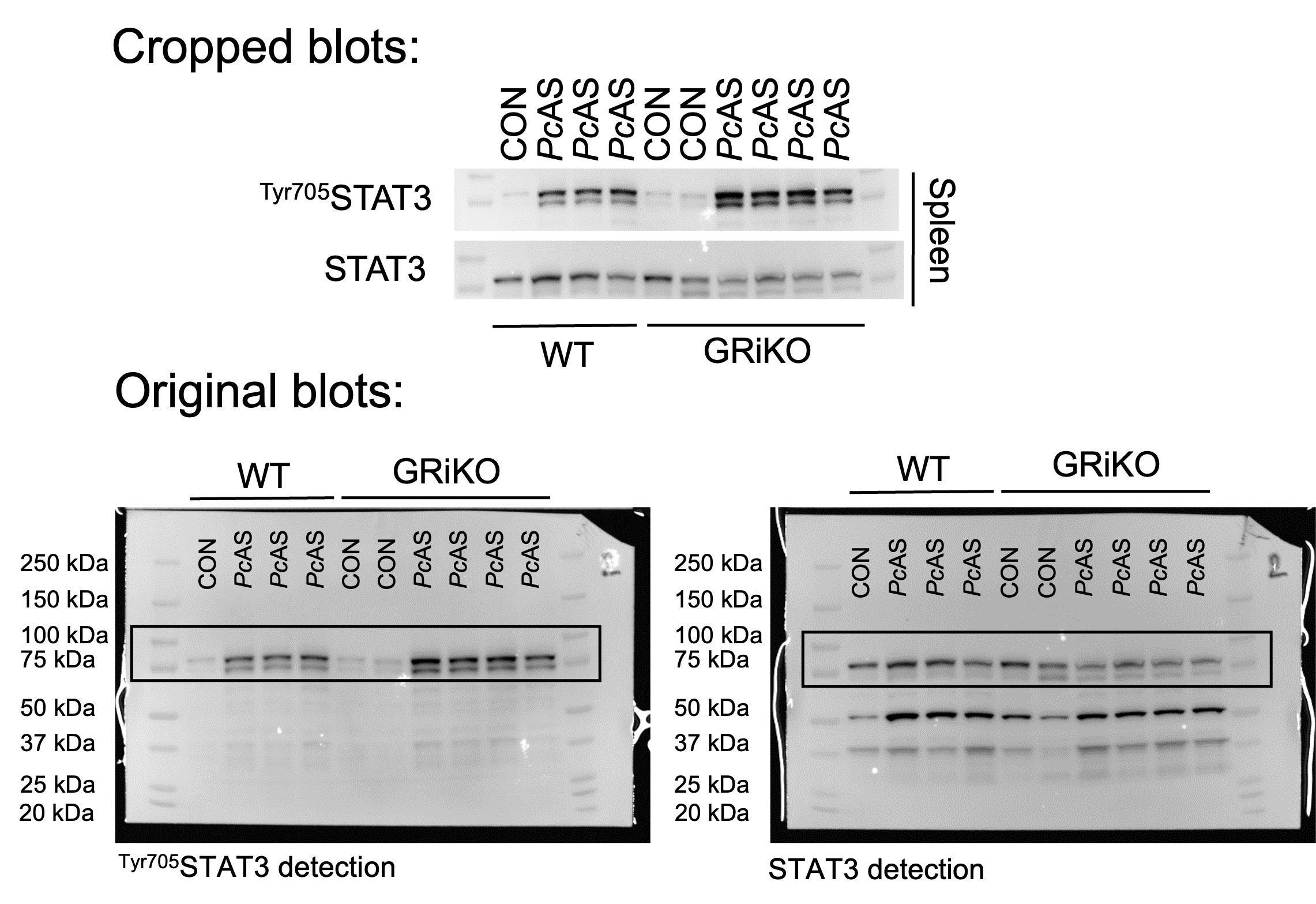

Supplement: Supplementary file 10 — Source data Fig. 6 [file 44321_2025_264_MOESM10_ESM.zip › Figure 6/6A/6A_Cropped western blot image.jpg]

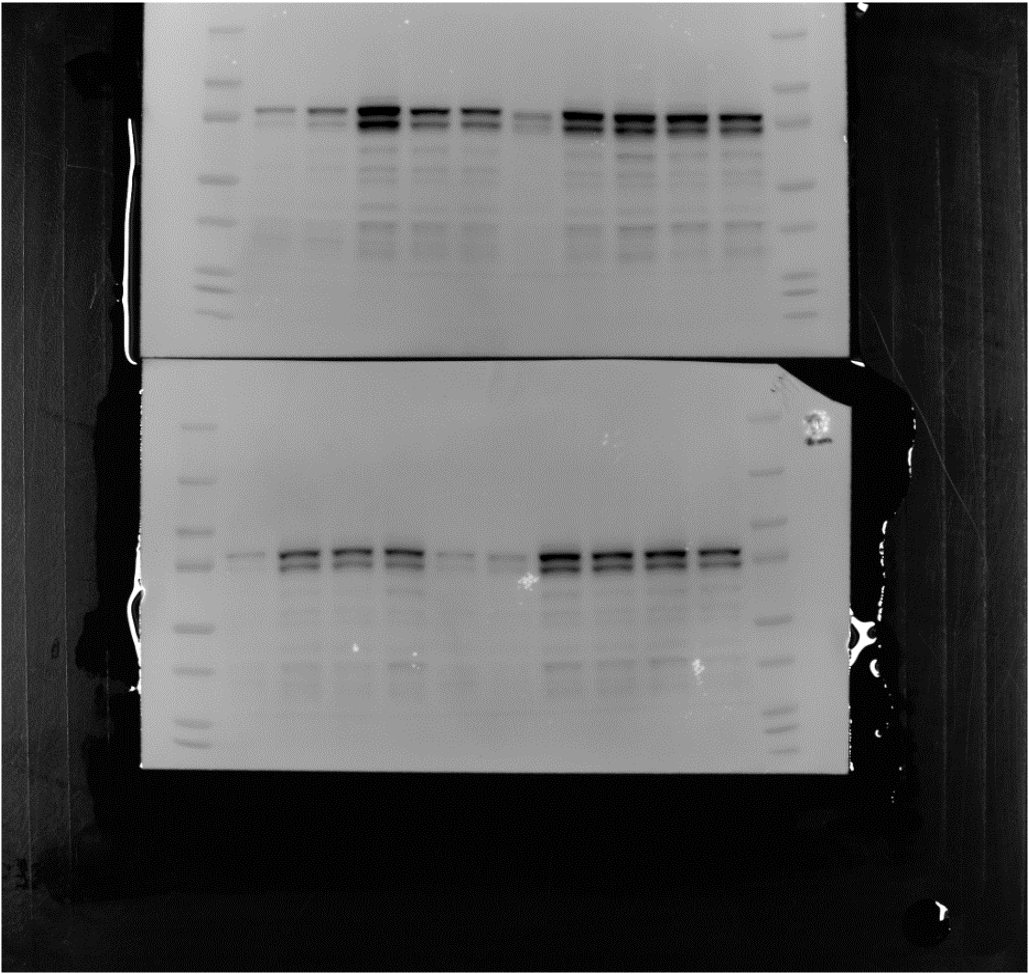

Supplement: Supplementary file 10 — Source data Fig. 6 [file 44321_2025_264_MOESM10_ESM.zip › Figure 6/6A/6A_pSTAT3_blot1+2.png]

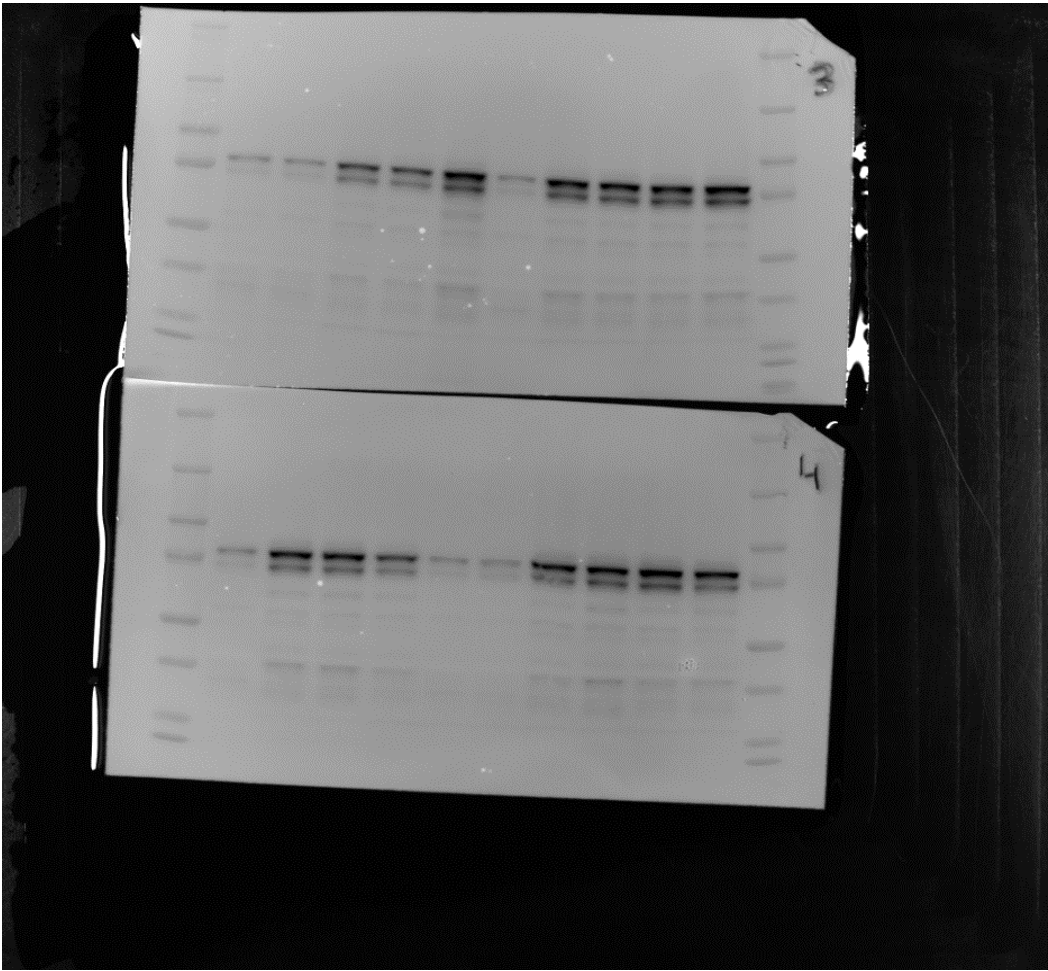

Supplement: Supplementary file 10 — Source data Fig. 6 [file 44321_2025_264_MOESM10_ESM.zip › Figure 6/6A/6A_pSTAT3_blot3+4.png]

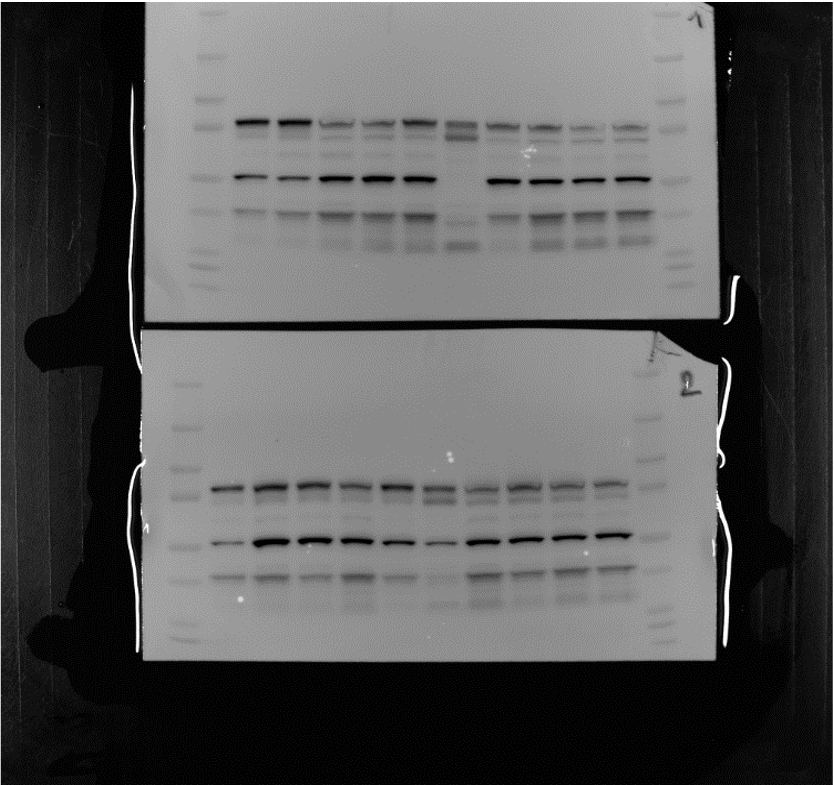

Supplement: Supplementary file 10 — Source data Fig. 6 [file 44321_2025_264_MOESM10_ESM.zip › Figure 6/6A/6A_STAT3_blot1+2.png]

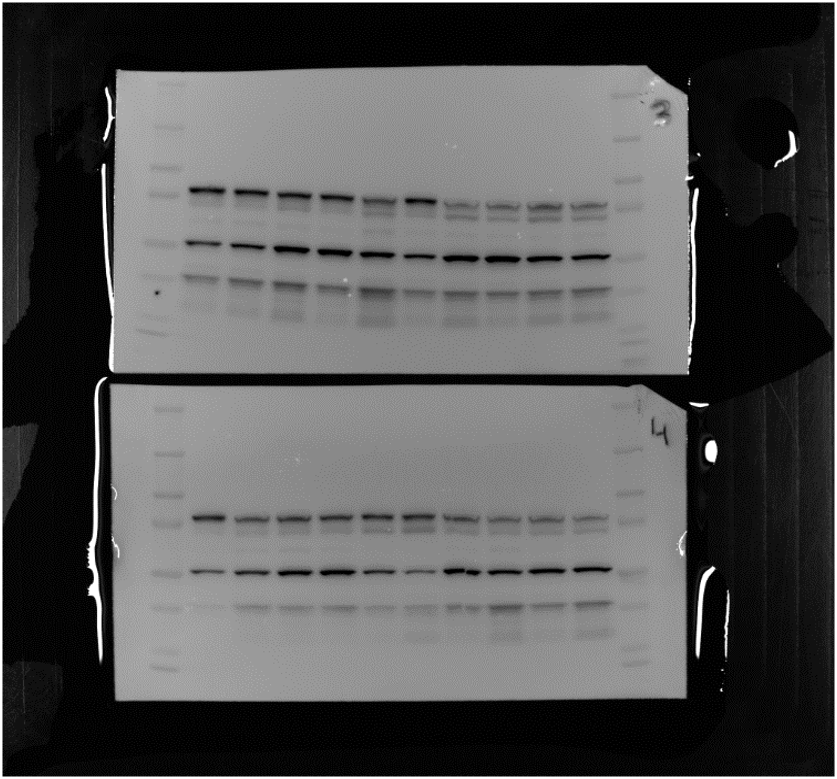

Supplement: Supplementary file 10 — Source data Fig. 6 [file 44321_2025_264_MOESM10_ESM.zip › Figure 6/6A/6A_STAT3_blot3+4.png]

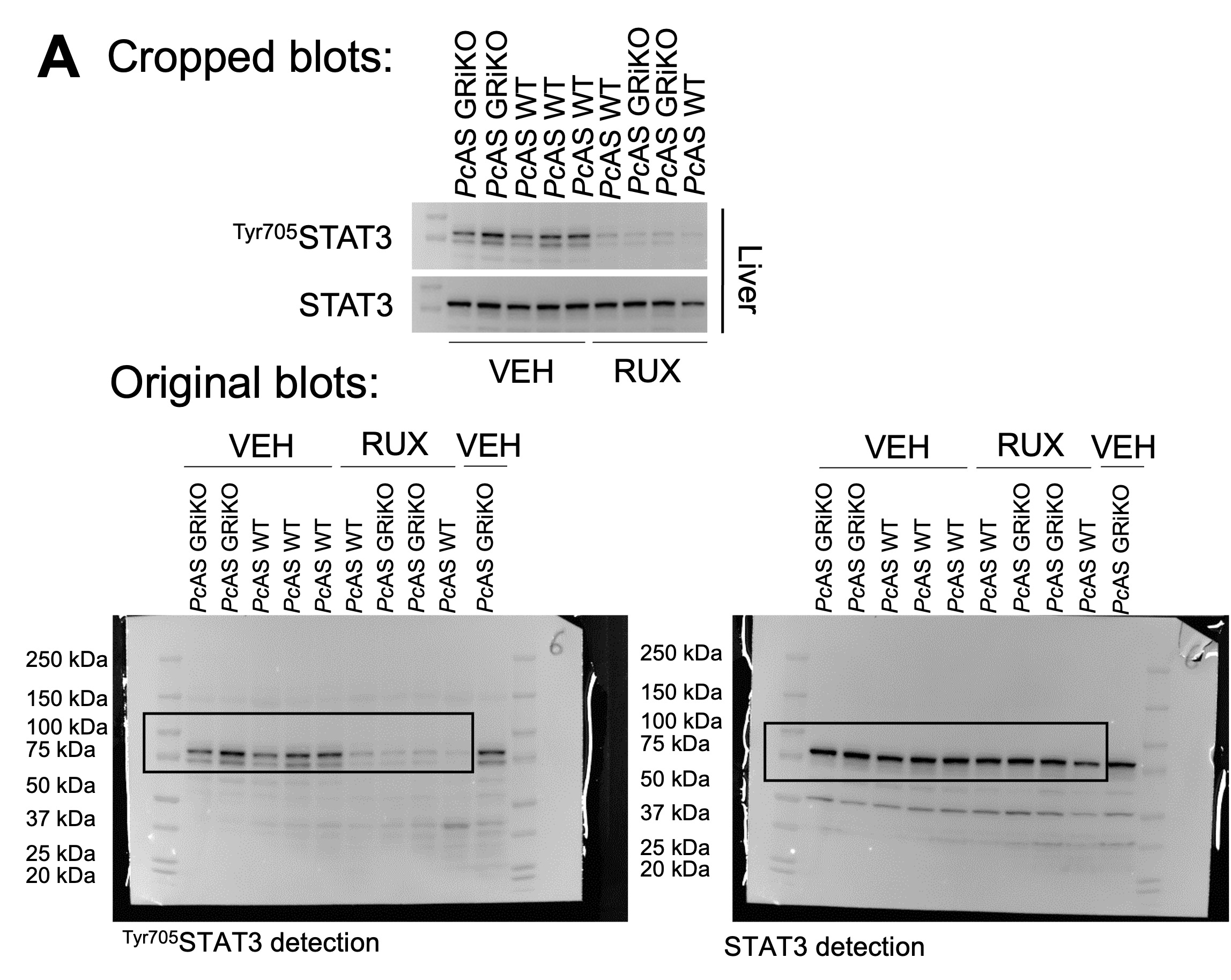

Supplement: Supplementary file 12 — Source data Fig. 8 [file 44321_2025_264_MOESM12_ESM.zip › Figure 8/8H/8H_Cropped western blot image.jpg]

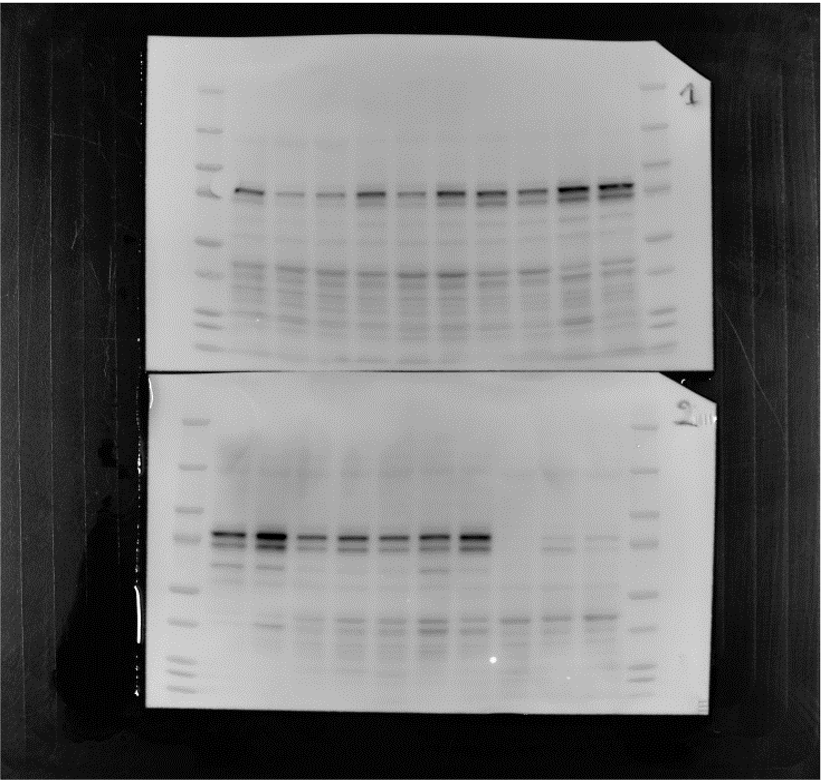

Supplement: Supplementary file 12 — Source data Fig. 8 [file 44321_2025_264_MOESM12_ESM.zip › Figure 8/8H/8H_Western blot_pSTAT3_gel1+2.png]

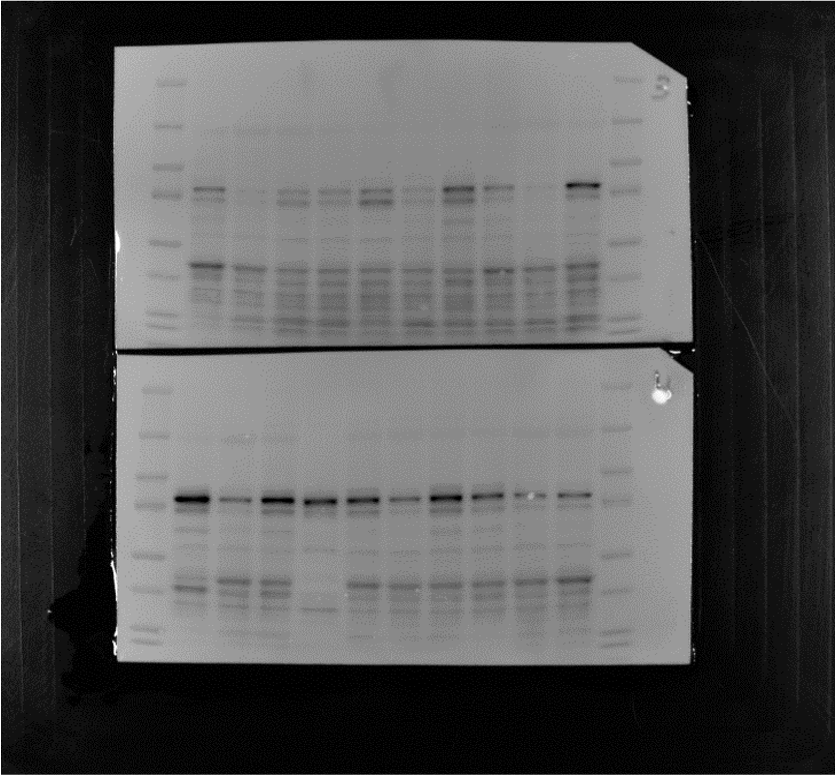

Supplement: Supplementary file 12 — Source data Fig. 8 [file 44321_2025_264_MOESM12_ESM.zip › Figure 8/8H/8H_Western blot_pSTAT3_gel3+4.png]

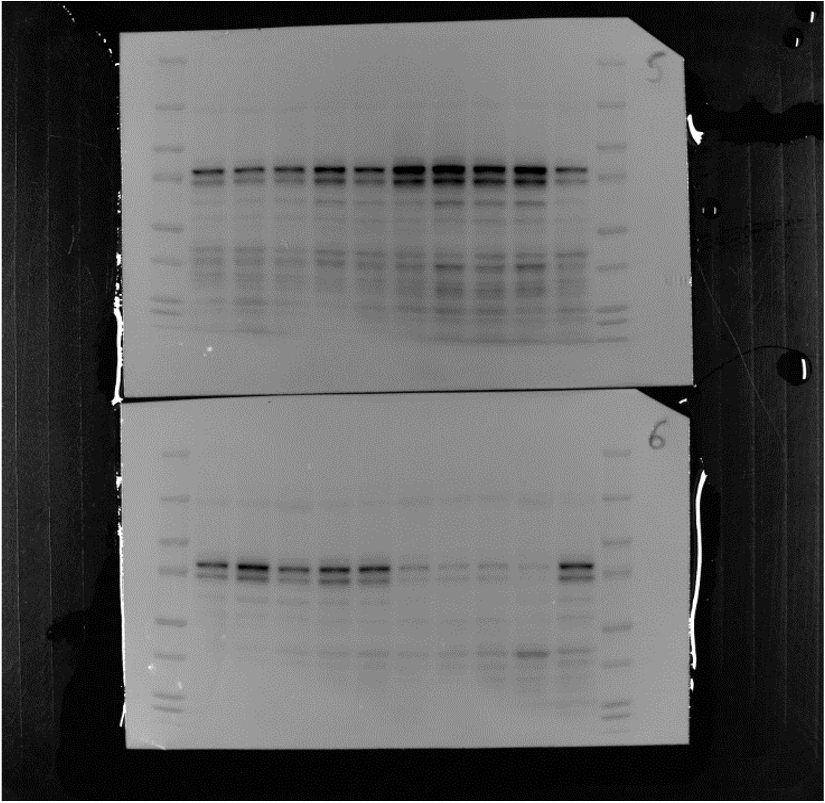

Supplement: Supplementary file 12 — Source data Fig. 8 [file 44321_2025_264_MOESM12_ESM.zip › Figure 8/8H/8H_Western blot_pSTAT3_gel5+6.png]

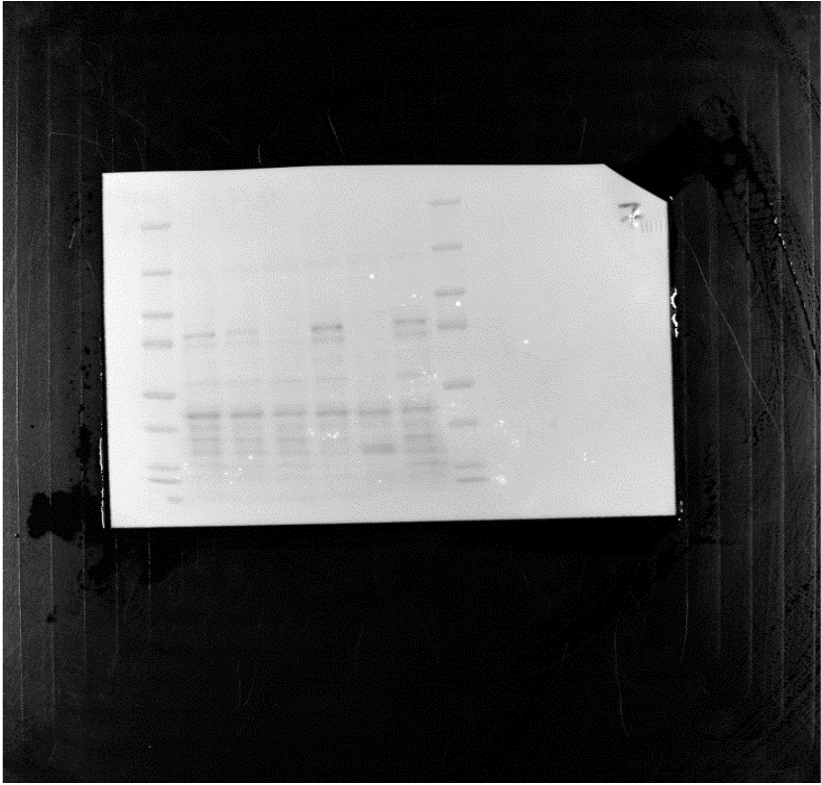

Supplement: Supplementary file 12 — Source data Fig. 8 [file 44321_2025_264_MOESM12_ESM.zip › Figure 8/8H/8H_Western blot_pSTAT3_gel7.png]

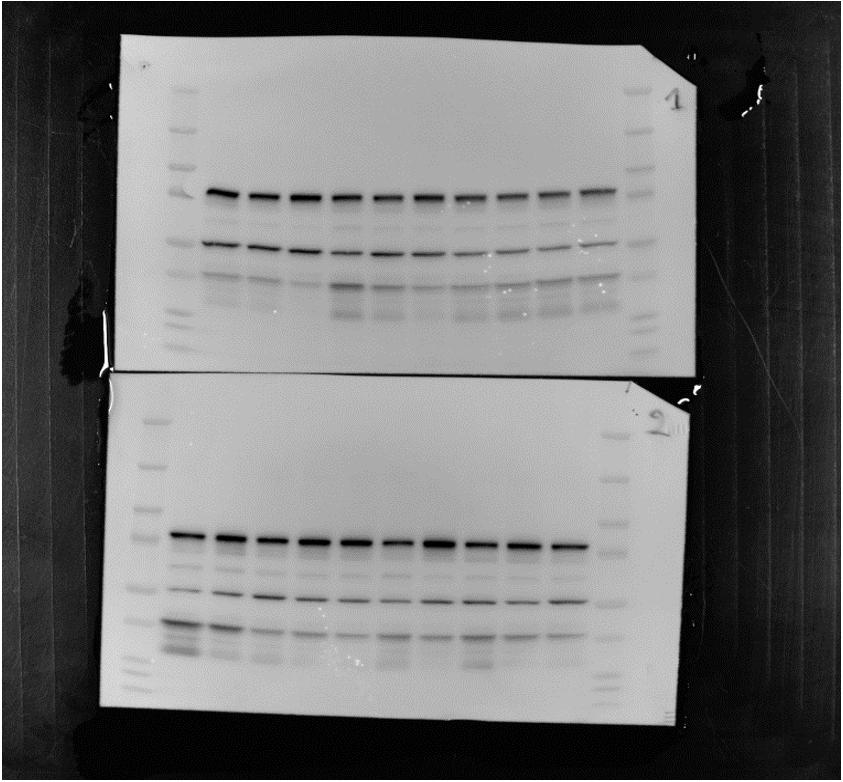

Supplement: Supplementary file 12 — Source data Fig. 8 [file 44321_2025_264_MOESM12_ESM.zip › Figure 8/8H/8H_Western blot_STAT3_gel1+2.png]

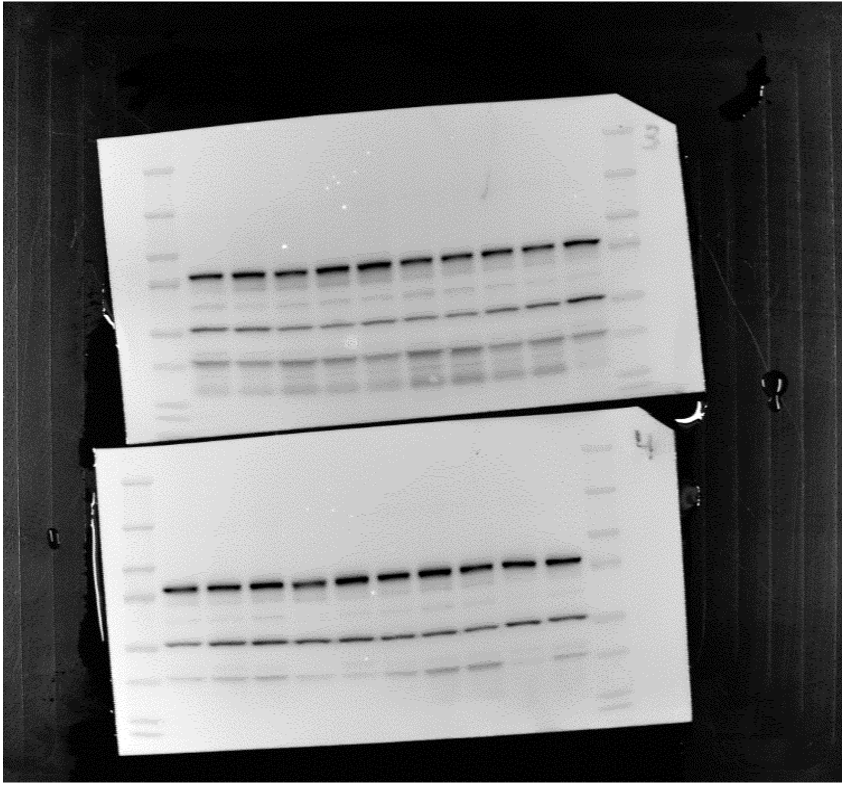

Supplement: Supplementary file 12 — Source data Fig. 8 [file 44321_2025_264_MOESM12_ESM.zip › Figure 8/8H/8H_Western blot_STAT3_gel3+4.png]

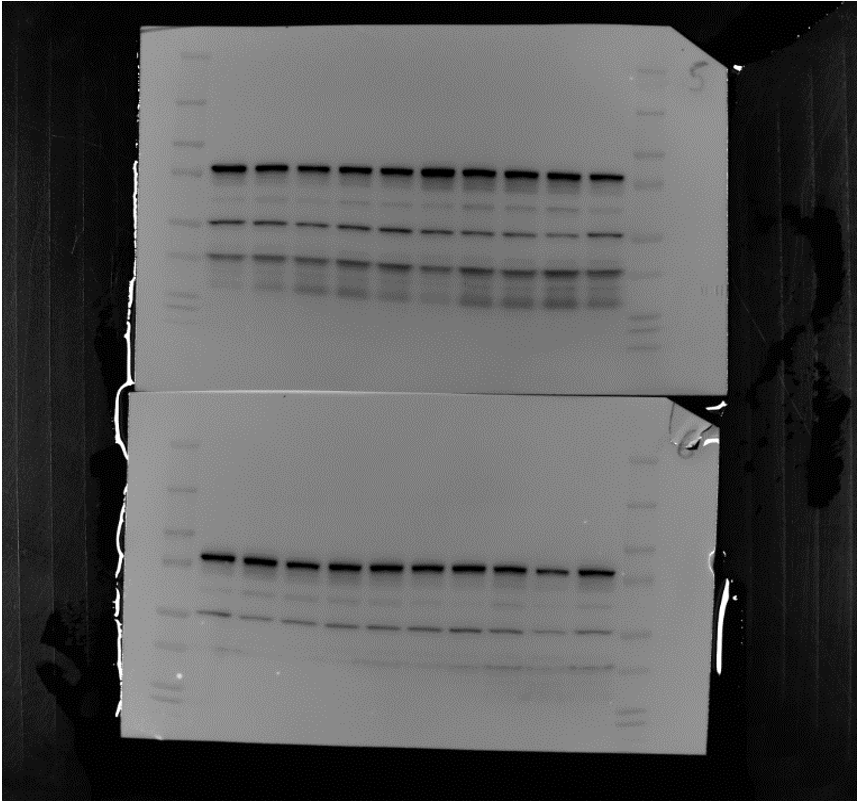

Supplement: Supplementary file 12 — Source data Fig. 8 [file 44321_2025_264_MOESM12_ESM.zip › Figure 8/8H/8H_Western blot_STAT3_gel5+6.png]

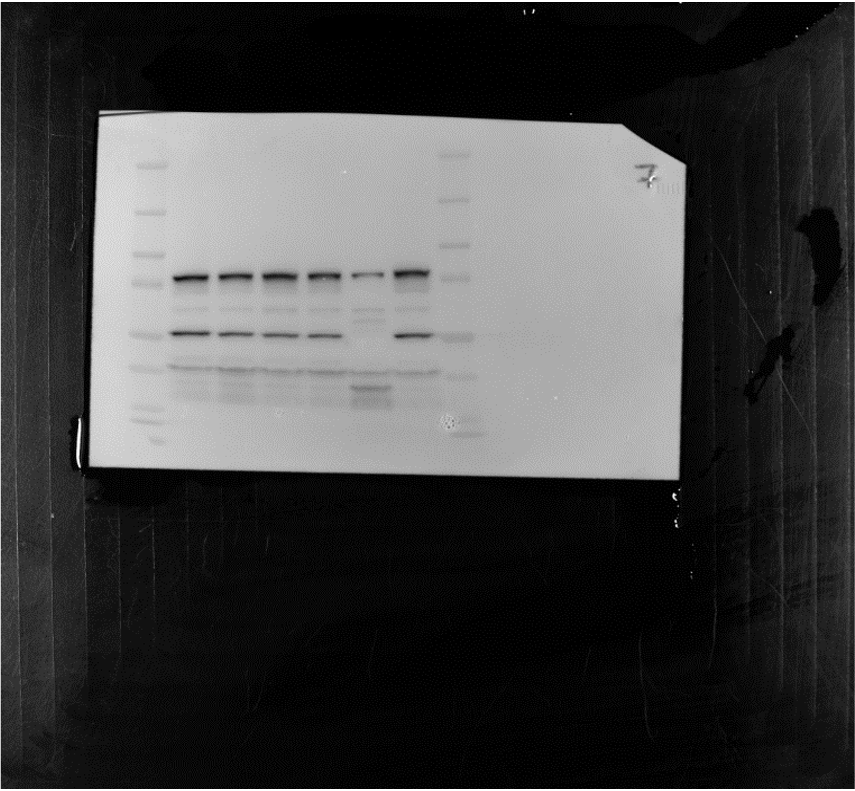

Supplement: Supplementary file 12 — Source data Fig. 8 [file 44321_2025_264_MOESM12_ESM.zip › Figure 8/8H/8H_Western blot_STAT3_gel7.png]

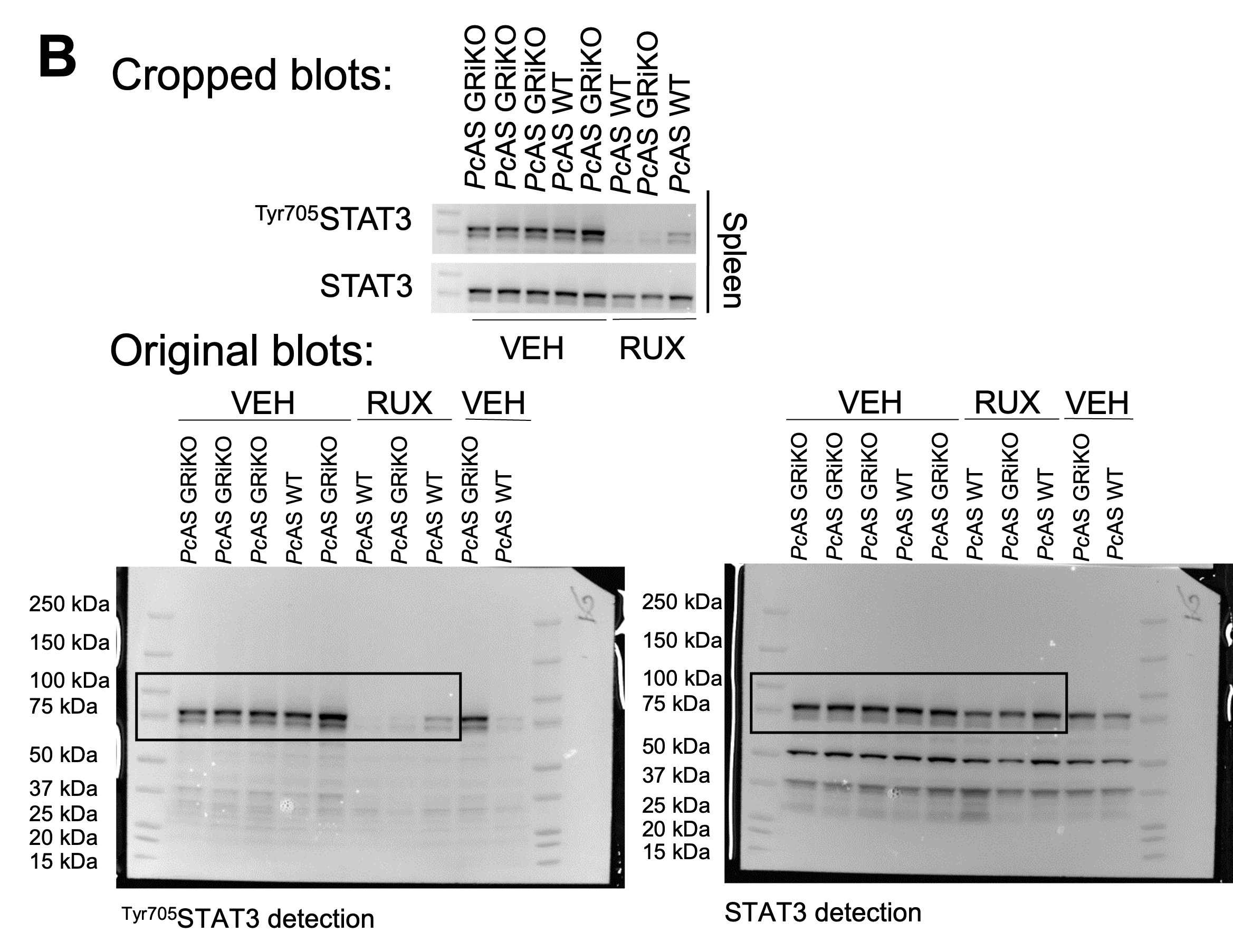

Supplement: Supplementary file 12 — Source data Fig. 8 [file 44321_2025_264_MOESM12_ESM.zip › Figure 8/8I/8I_Cropped western blot image.jpg]

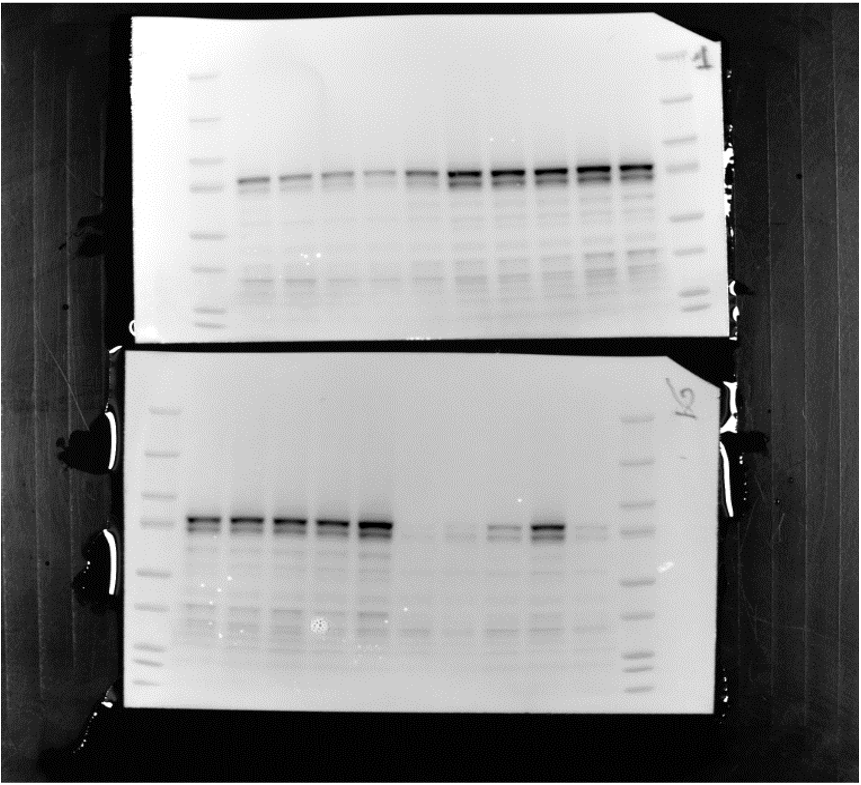

Supplement: Supplementary file 12 — Source data Fig. 8 [file 44321_2025_264_MOESM12_ESM.zip › Figure 8/8I/8I_Western blot_pSTAT3_gel1+2.png]

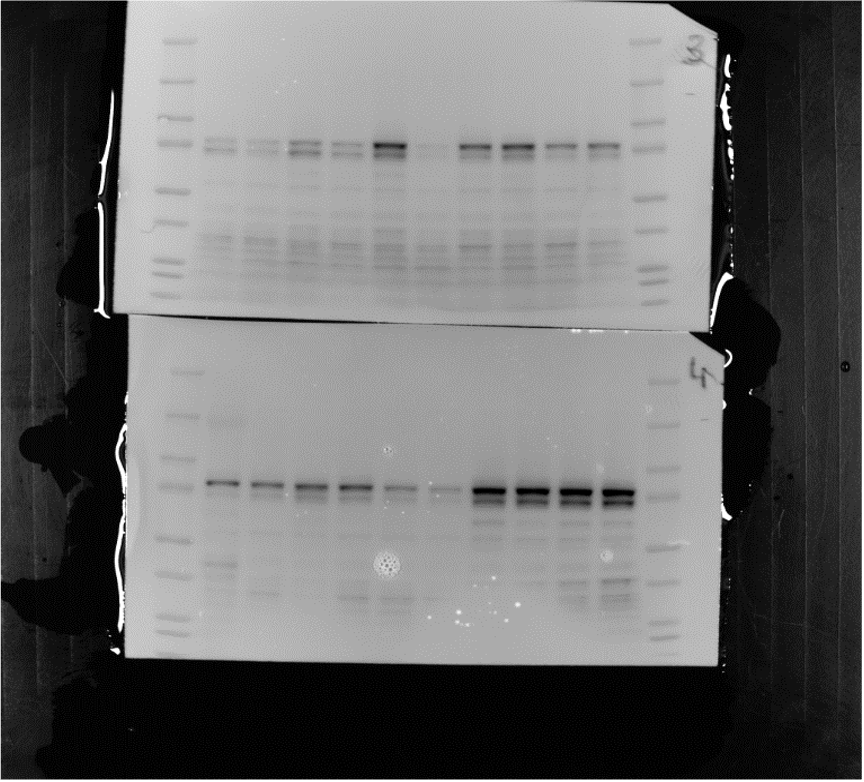

Supplement: Supplementary file 12 — Source data Fig. 8 [file 44321_2025_264_MOESM12_ESM.zip › Figure 8/8I/8I_Western blot_pSTAT3_gel3+4.png]

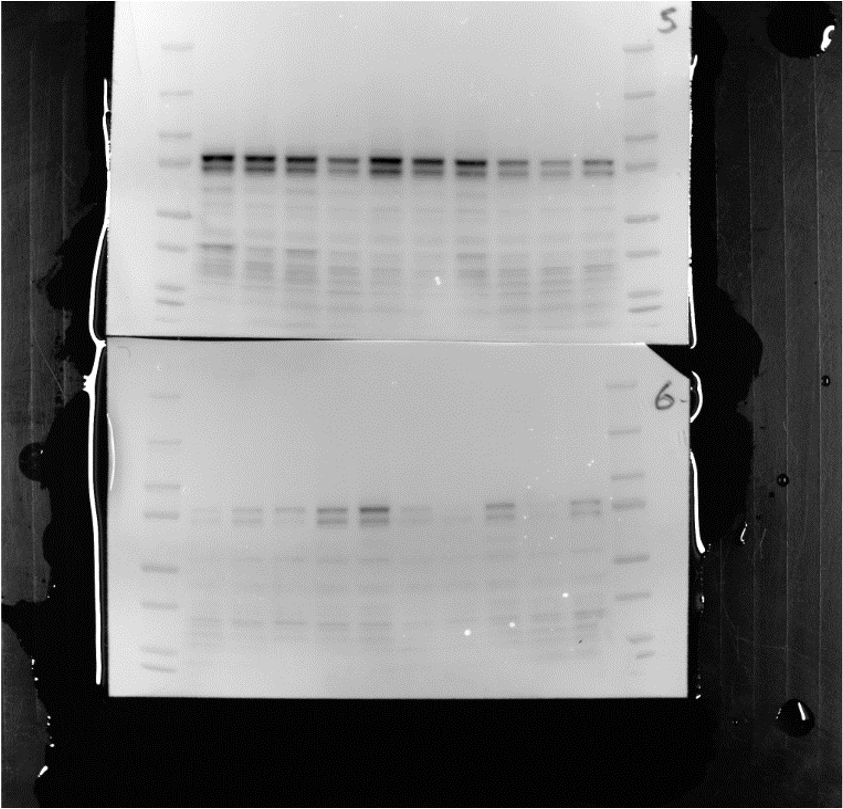

Supplement: Supplementary file 12 — Source data Fig. 8 [file 44321_2025_264_MOESM12_ESM.zip › Figure 8/8I/8I_Western blot_pSTAT3_gel5+6.png]

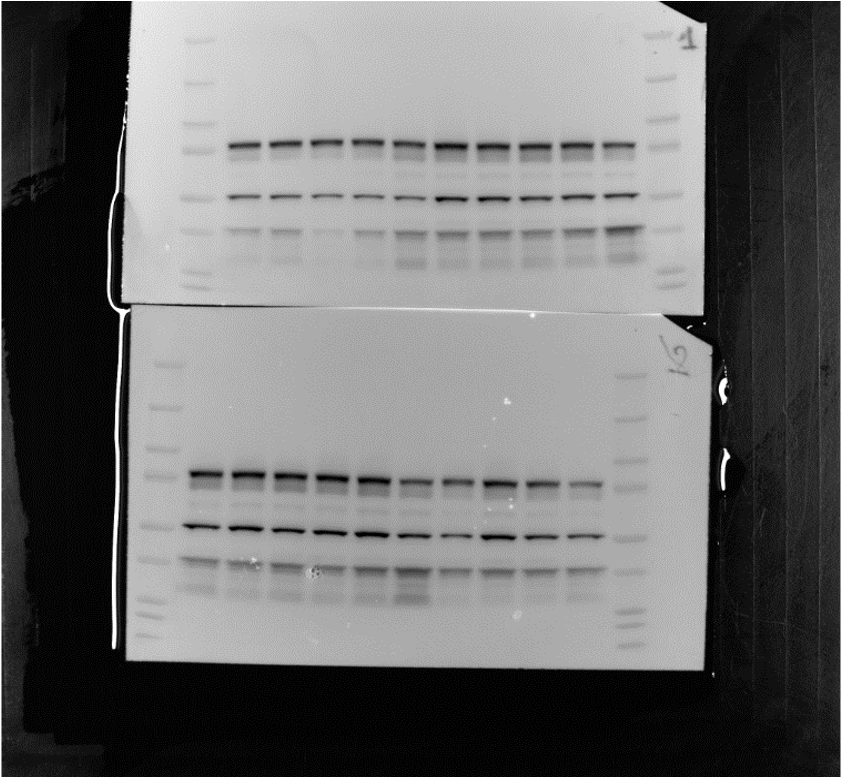

Supplement: Supplementary file 12 — Source data Fig. 8 [file 44321_2025_264_MOESM12_ESM.zip › Figure 8/8I/8I_Western blot_STAT3_gel1+2.png]

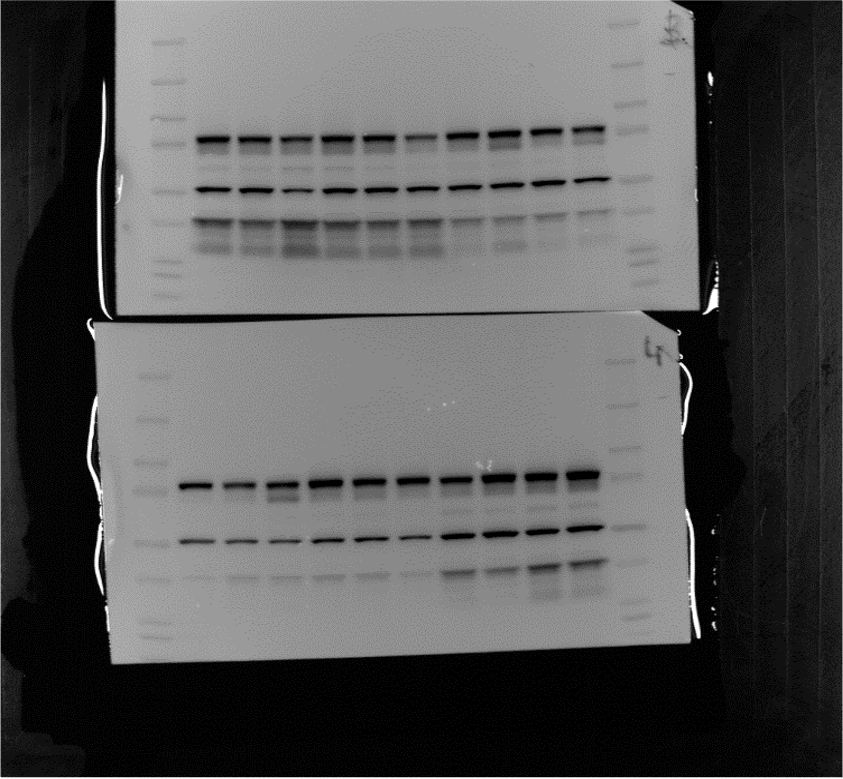

Supplement: Supplementary file 12 — Source data Fig. 8 [file 44321_2025_264_MOESM12_ESM.zip › Figure 8/8I/8I_Western blot_STAT3_gel3+4.png]

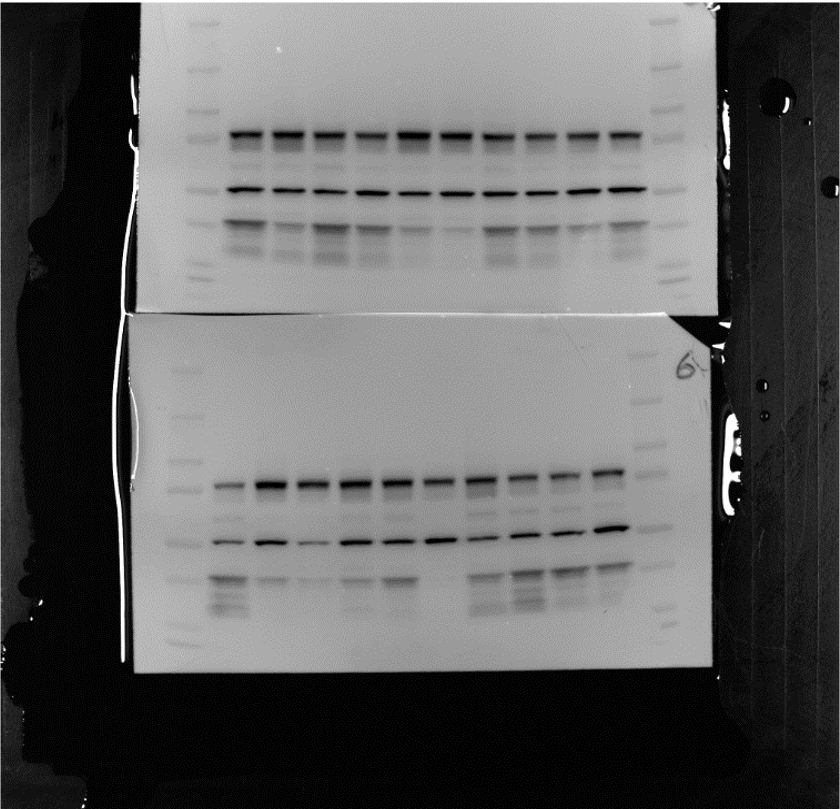

Supplement: Supplementary file 12 — Source data Fig. 8 [file 44321_2025_264_MOESM12_ESM.zip › Figure 8/8I/8I_Western blot_STAT3_gel5+6.png]

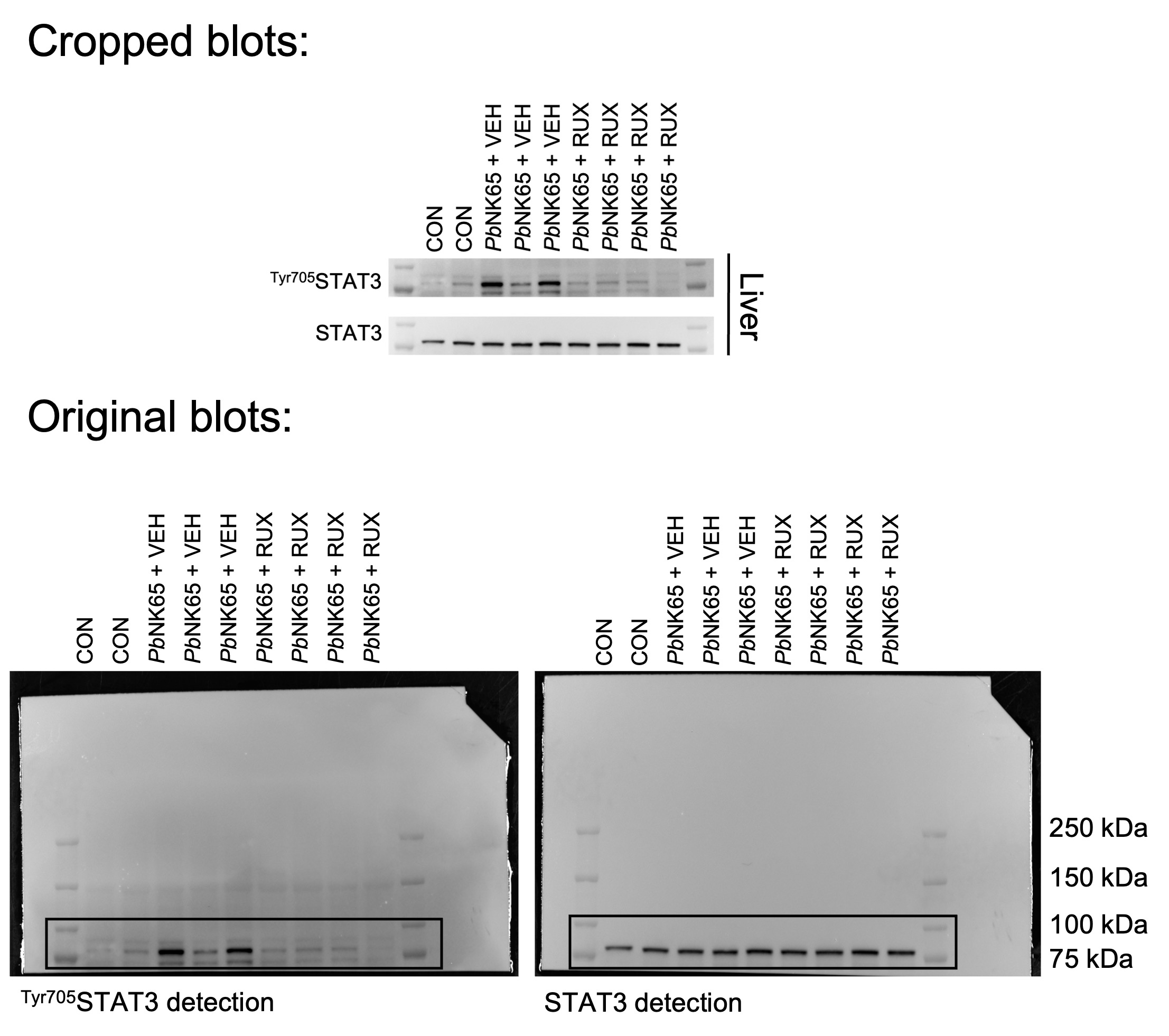

Supplement: Supplementary file 14 — Source data Fig. 10 [file 44321_2025_264_MOESM14_ESM.zip › Figure 10/10B/10B_Western blot cropped image.jpg]

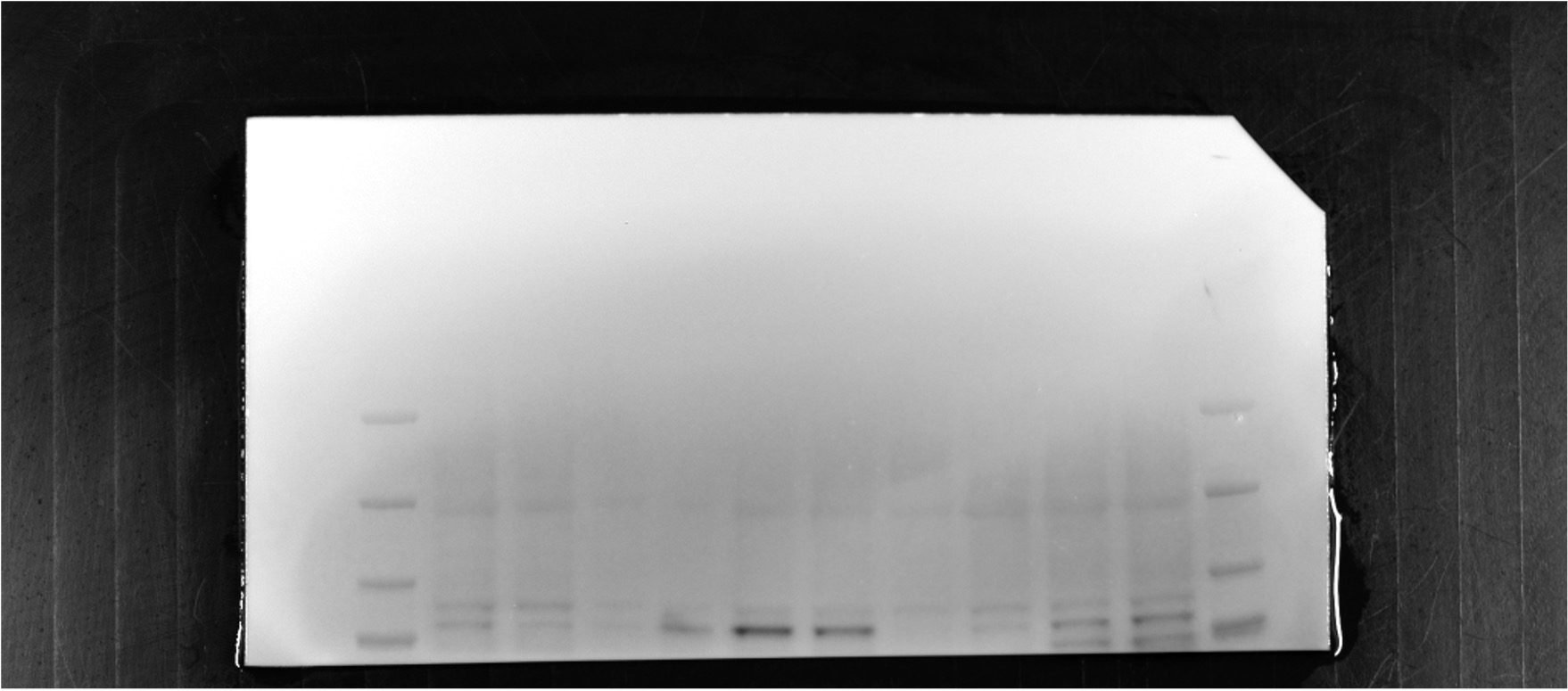

Supplement: Supplementary file 14 — Source data Fig. 10 [file 44321_2025_264_MOESM14_ESM.zip › Figure 10/10B/10B_Western blot image pSTAT3_Gel 1.tiff]

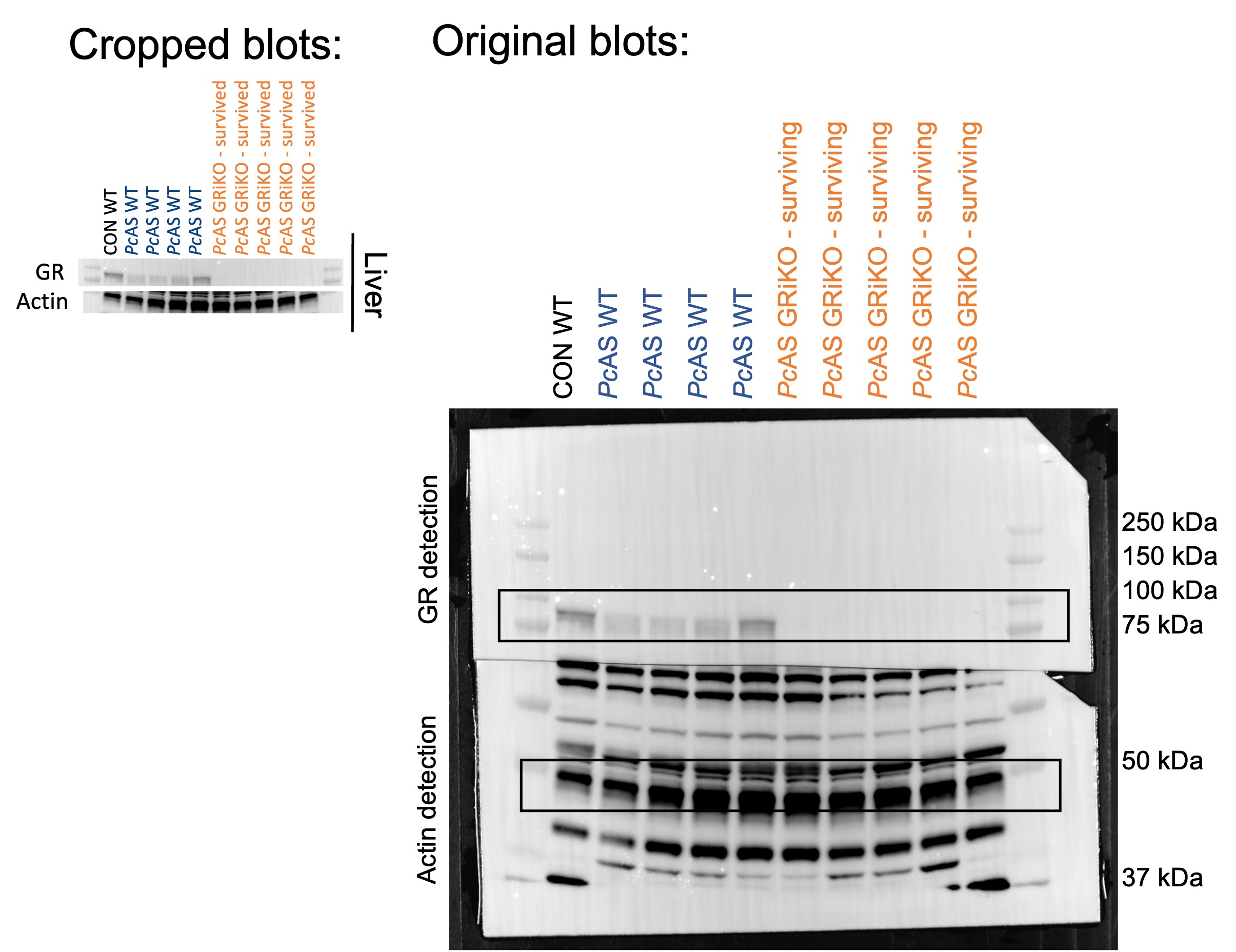

Supplement: Supplementary file 15 — EV and Appendix Figures Source Data [file 44321_2025_264_MOESM15_ESM.zip › EMM-2024-20682_SourceDataForExpandedViewAndAppendix/Sourcedata Appendix fig S2/Appendix S2_Cropped WB.jpg]

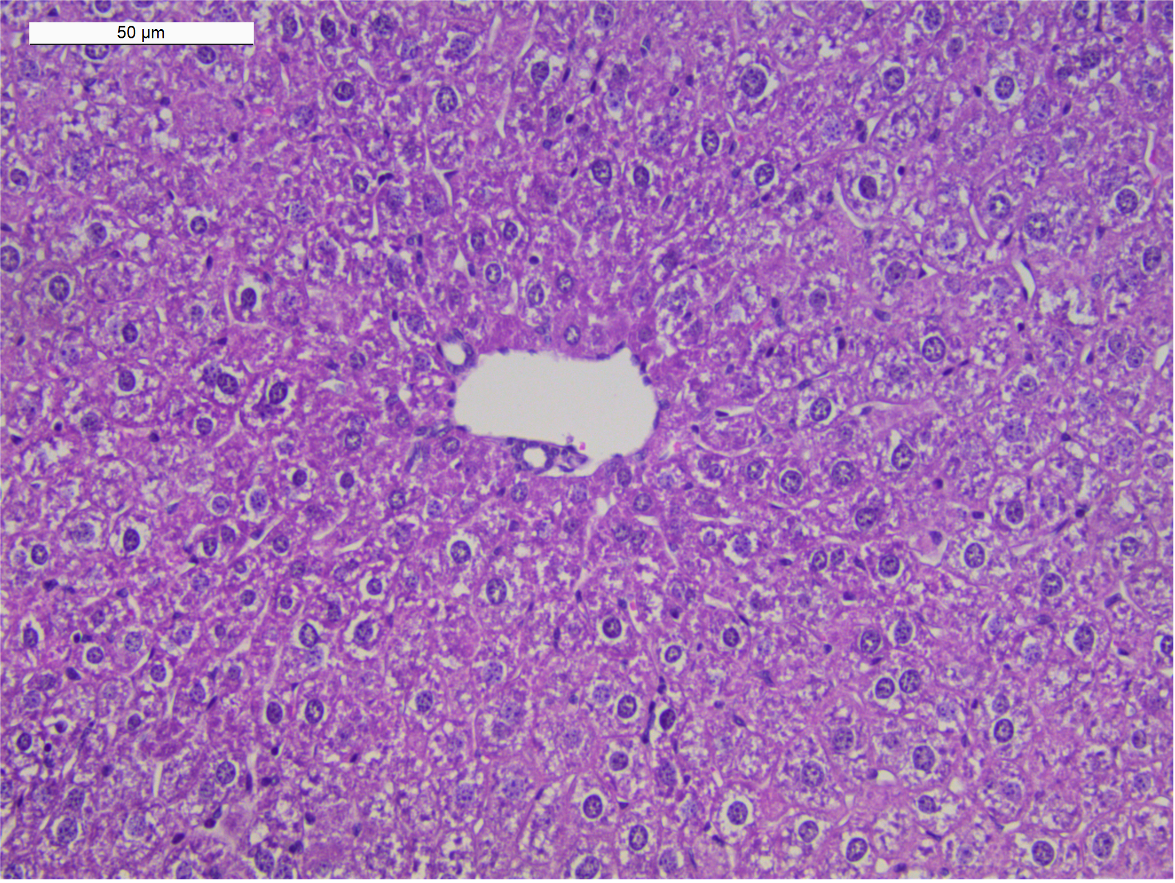

Supplement: Supplementary file 15 — EV and Appendix Figures Source Data [file 44321_2025_264_MOESM15_ESM.zip › EMM-2024-20682_SourceDataForExpandedViewAndAppendix/Sourcedata Appendix fig S3/Appendix S3_H&E_CON GRiKO1.png]

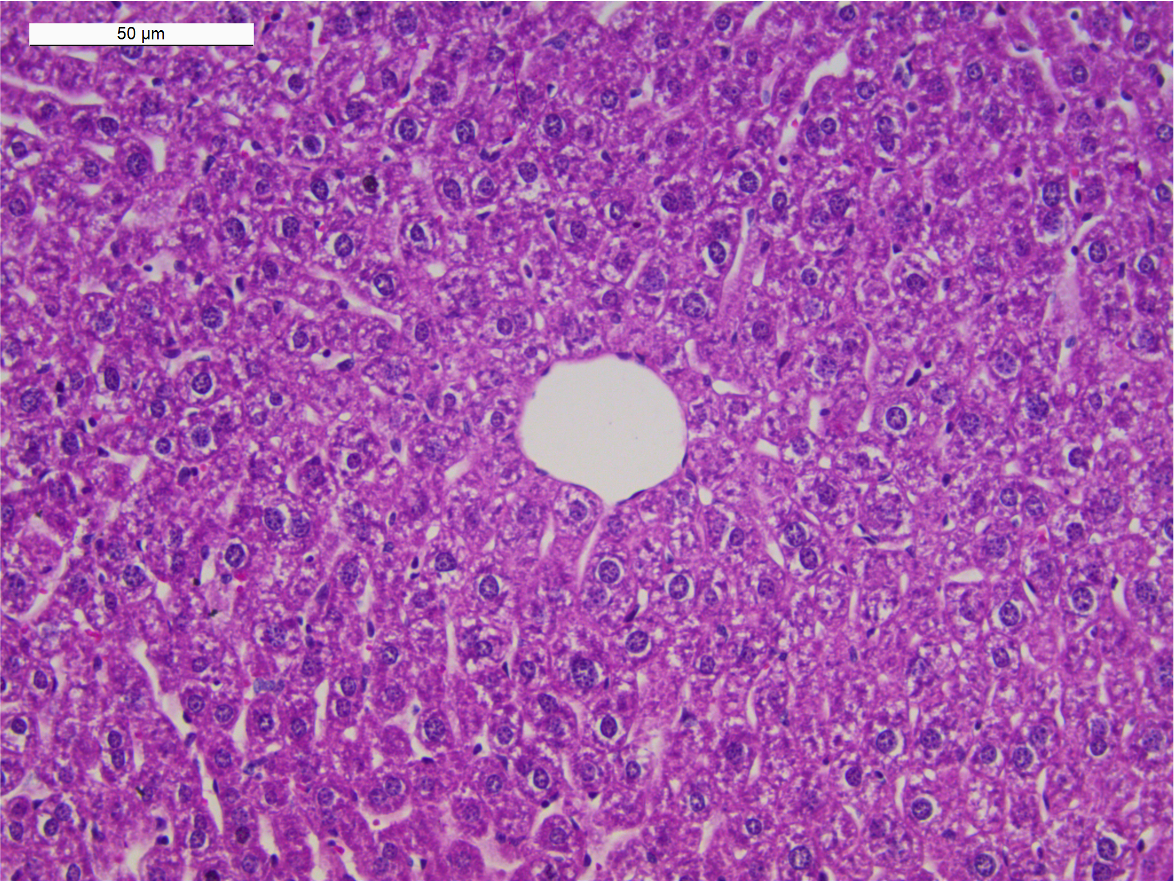

Supplement: Supplementary file 15 — EV and Appendix Figures Source Data [file 44321_2025_264_MOESM15_ESM.zip › EMM-2024-20682_SourceDataForExpandedViewAndAppendix/Sourcedata Appendix fig S3/Appendix S3_H&E_CON GRiKO2.png]

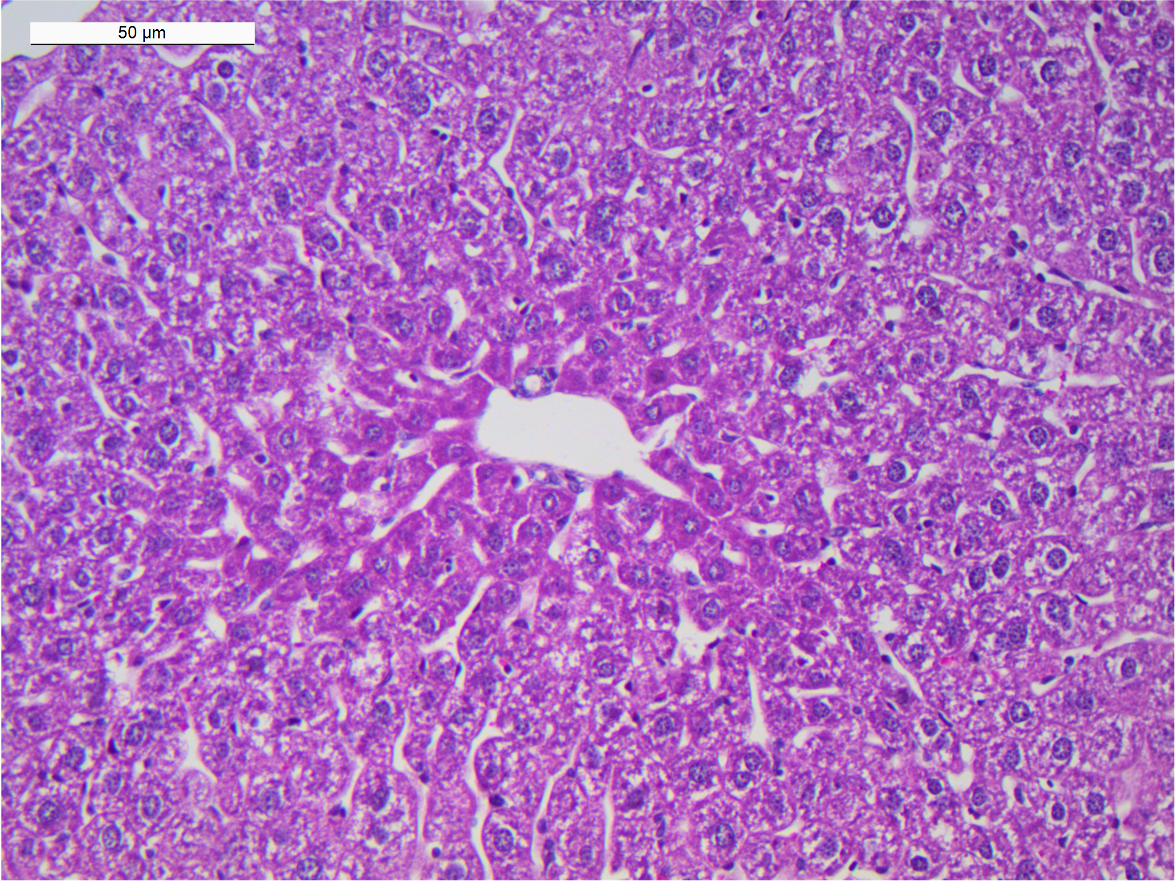

Supplement: Supplementary file 15 — EV and Appendix Figures Source Data [file 44321_2025_264_MOESM15_ESM.zip › EMM-2024-20682_SourceDataForExpandedViewAndAppendix/Sourcedata Appendix fig S3/Appendix S3_H&E_CON GRiKO3.png]

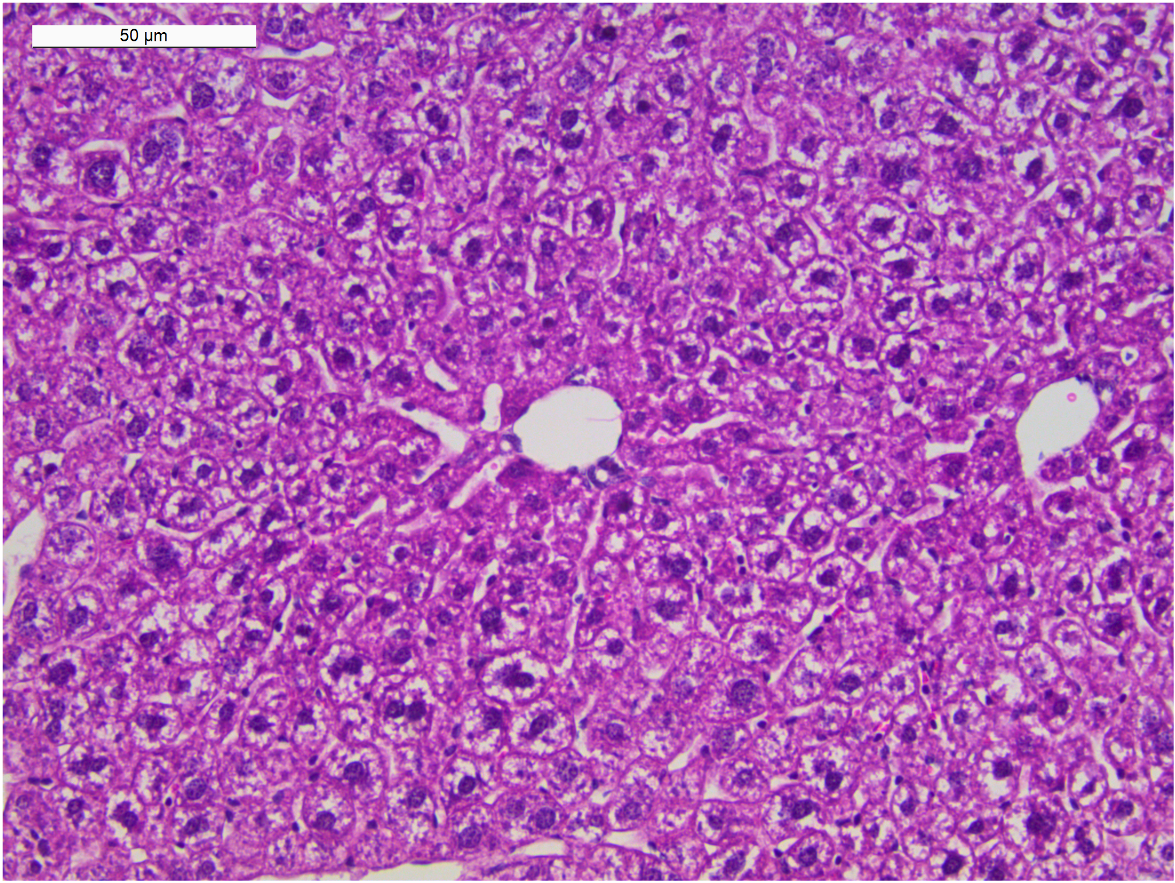

Supplement: Supplementary file 15 — EV and Appendix Figures Source Data [file 44321_2025_264_MOESM15_ESM.zip › EMM-2024-20682_SourceDataForExpandedViewAndAppendix/Sourcedata Appendix fig S3/Appendix S3_H&E_CON WT1.png]

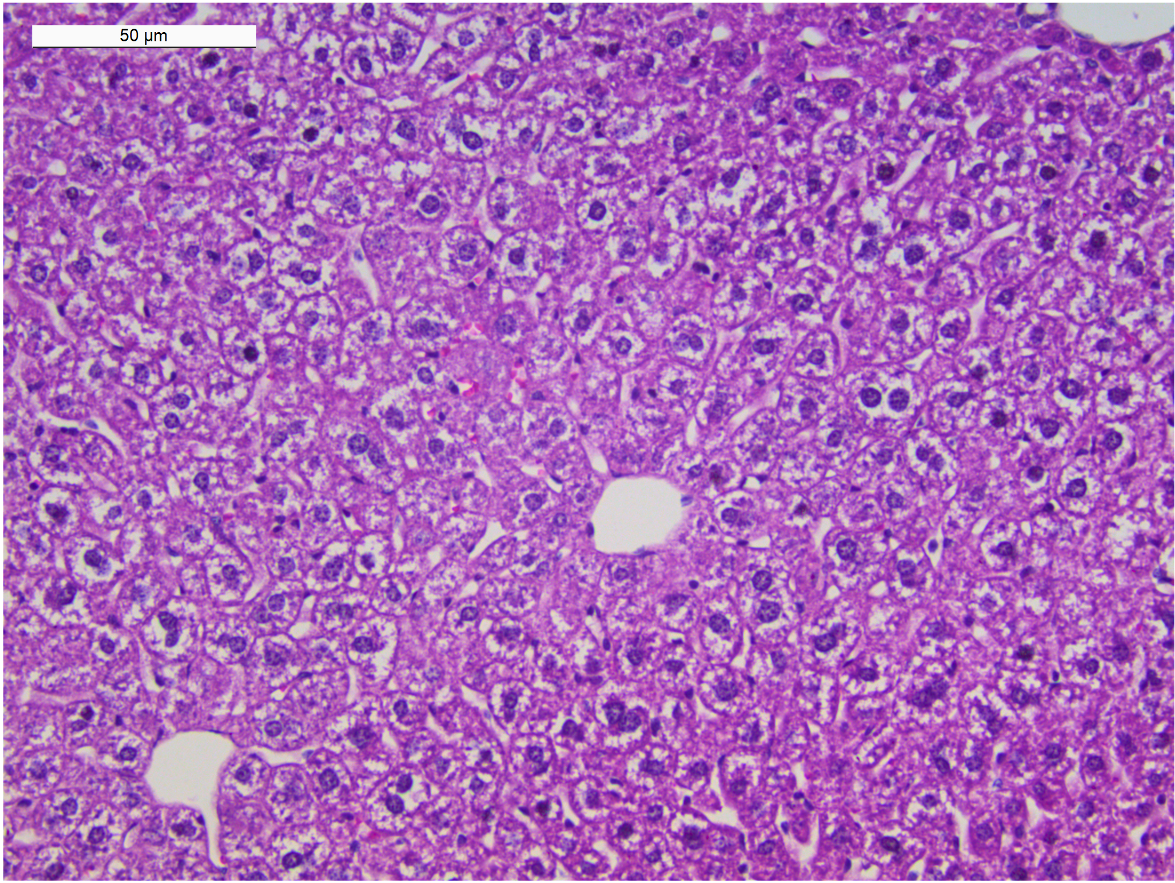

Supplement: Supplementary file 15 — EV and Appendix Figures Source Data [file 44321_2025_264_MOESM15_ESM.zip › EMM-2024-20682_SourceDataForExpandedViewAndAppendix/Sourcedata Appendix fig S3/Appendix S3_H&E_CON WT2.png]

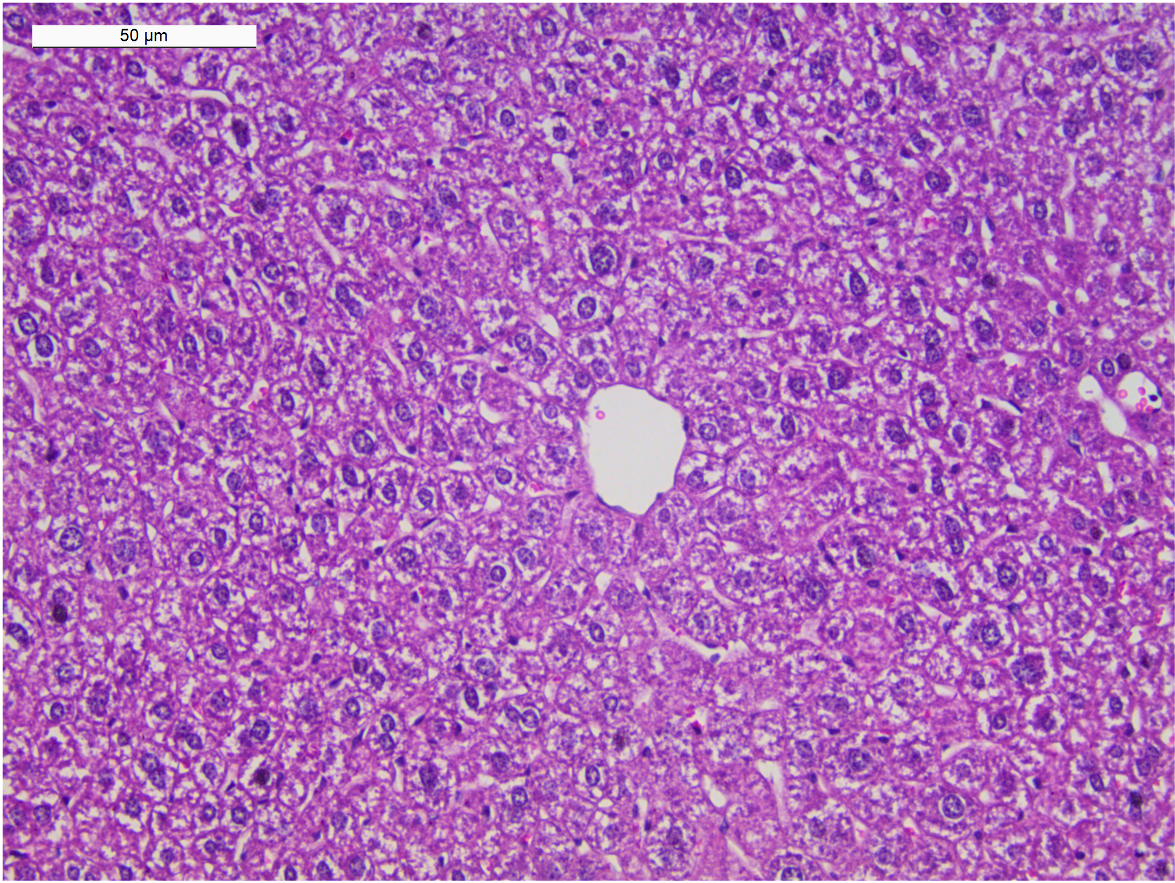

Supplement: Supplementary file 15 — EV and Appendix Figures Source Data [file 44321_2025_264_MOESM15_ESM.zip › EMM-2024-20682_SourceDataForExpandedViewAndAppendix/Sourcedata Appendix fig S3/Appendix S3_H&E_CON WT3.png]

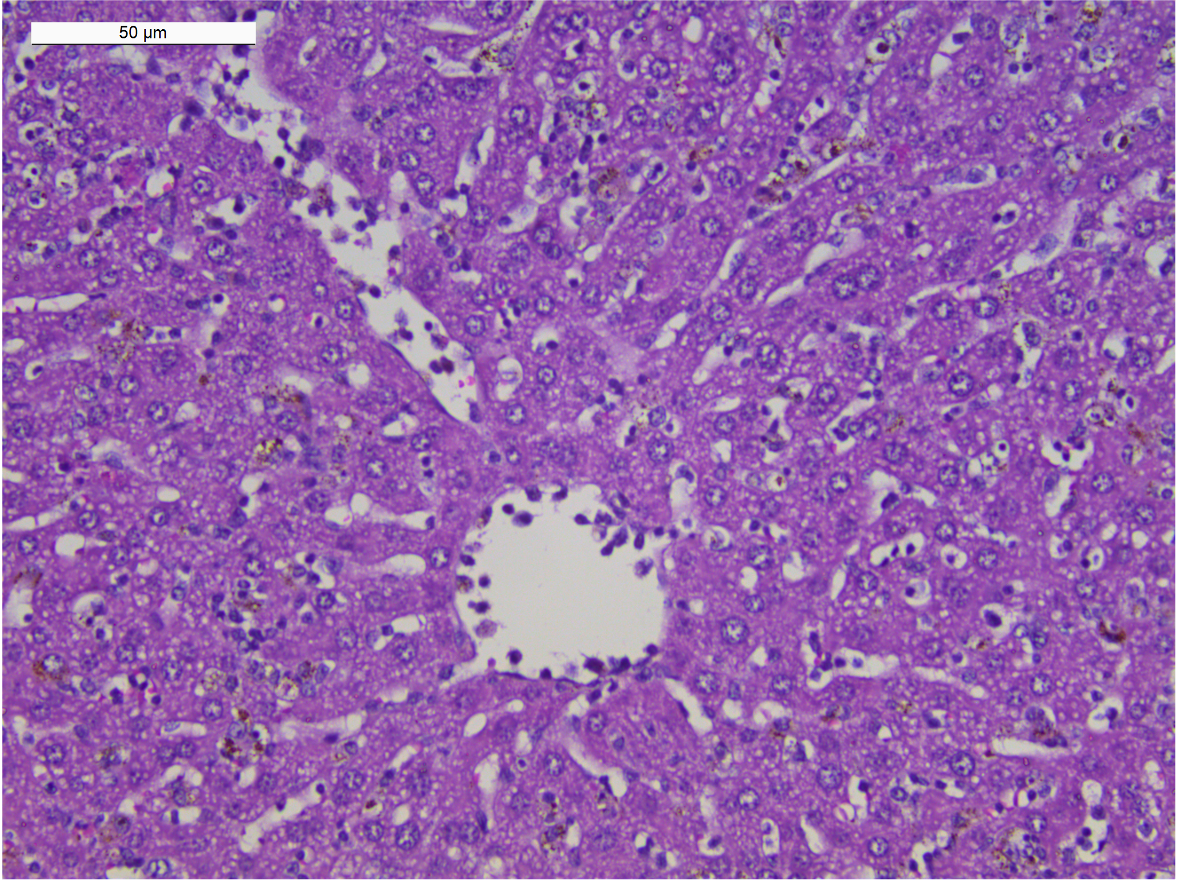

Supplement: Supplementary file 15 — EV and Appendix Figures Source Data [file 44321_2025_264_MOESM15_ESM.zip › EMM-2024-20682_SourceDataForExpandedViewAndAppendix/Sourcedata Appendix fig S3/Appendix S3_H&E_PCAS GRiKO1.png]

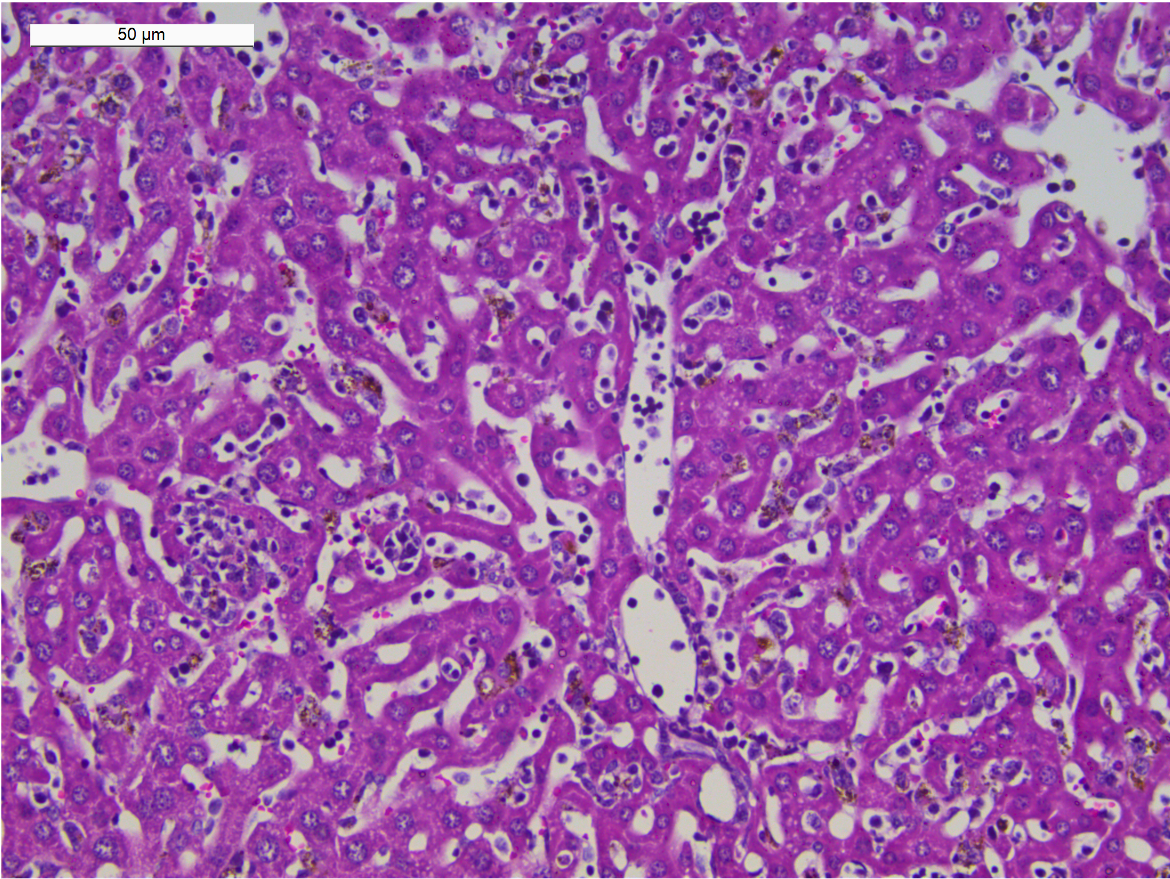

Supplement: Supplementary file 15 — EV and Appendix Figures Source Data [file 44321_2025_264_MOESM15_ESM.zip › EMM-2024-20682_SourceDataForExpandedViewAndAppendix/Sourcedata Appendix fig S3/Appendix S3_H&E_PCAS GRiKO2.png]

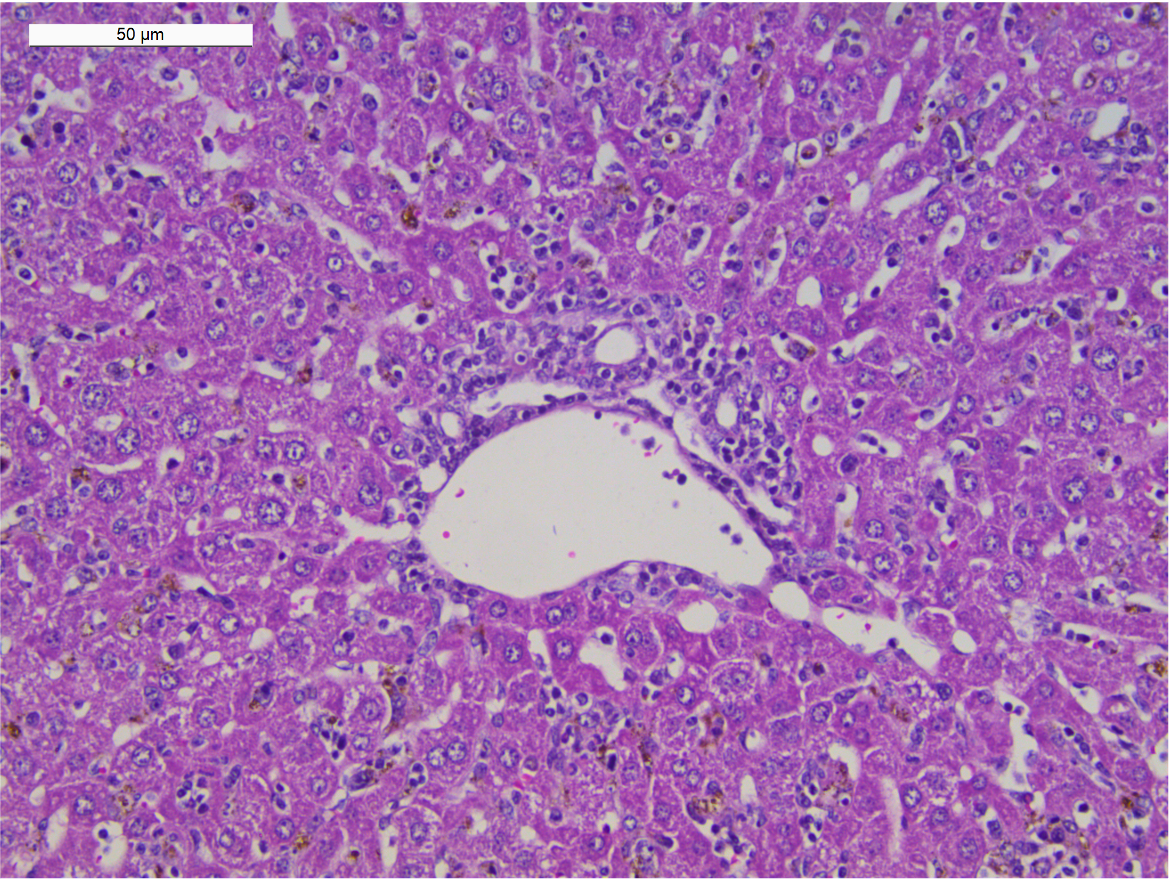

Supplement: Supplementary file 15 — EV and Appendix Figures Source Data [file 44321_2025_264_MOESM15_ESM.zip › EMM-2024-20682_SourceDataForExpandedViewAndAppendix/Sourcedata Appendix fig S3/Appendix S3_H&E_PCAS GRiKO3.png]

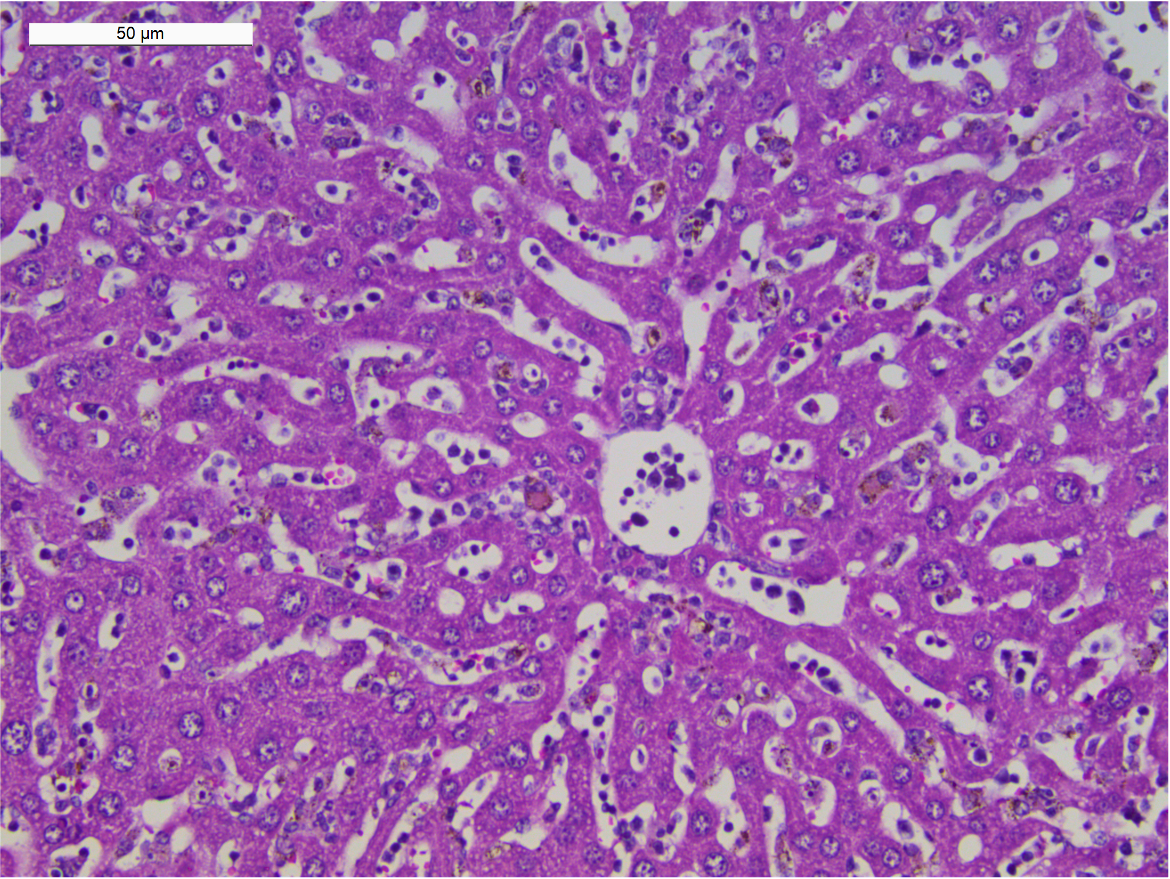

Supplement: Supplementary file 15 — EV and Appendix Figures Source Data [file 44321_2025_264_MOESM15_ESM.zip › EMM-2024-20682_SourceDataForExpandedViewAndAppendix/Sourcedata Appendix fig S3/Appendix S3_H&E_PCAS GRiKO4.png]

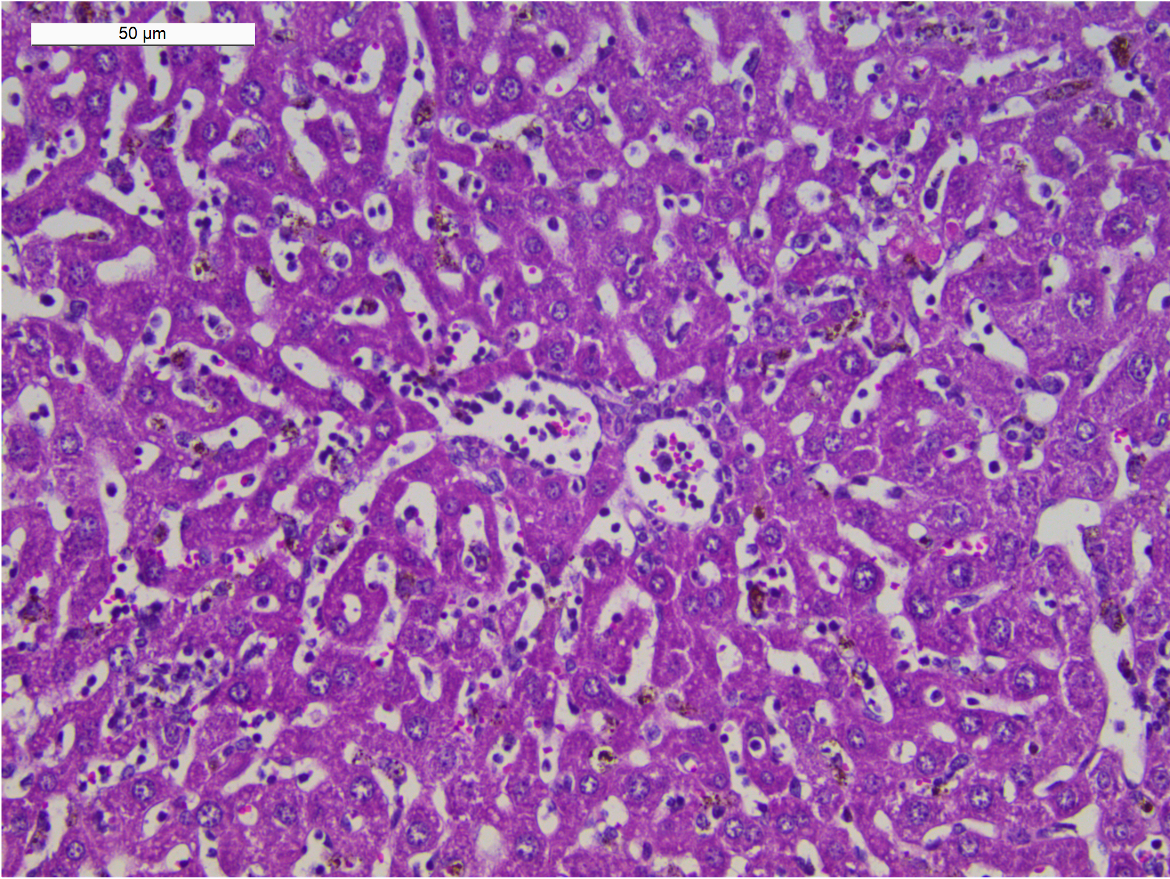

Supplement: Supplementary file 15 — EV and Appendix Figures Source Data [file 44321_2025_264_MOESM15_ESM.zip › EMM-2024-20682_SourceDataForExpandedViewAndAppendix/Sourcedata Appendix fig S3/Appendix S3_H&E_PCAS GRiKO5.png]

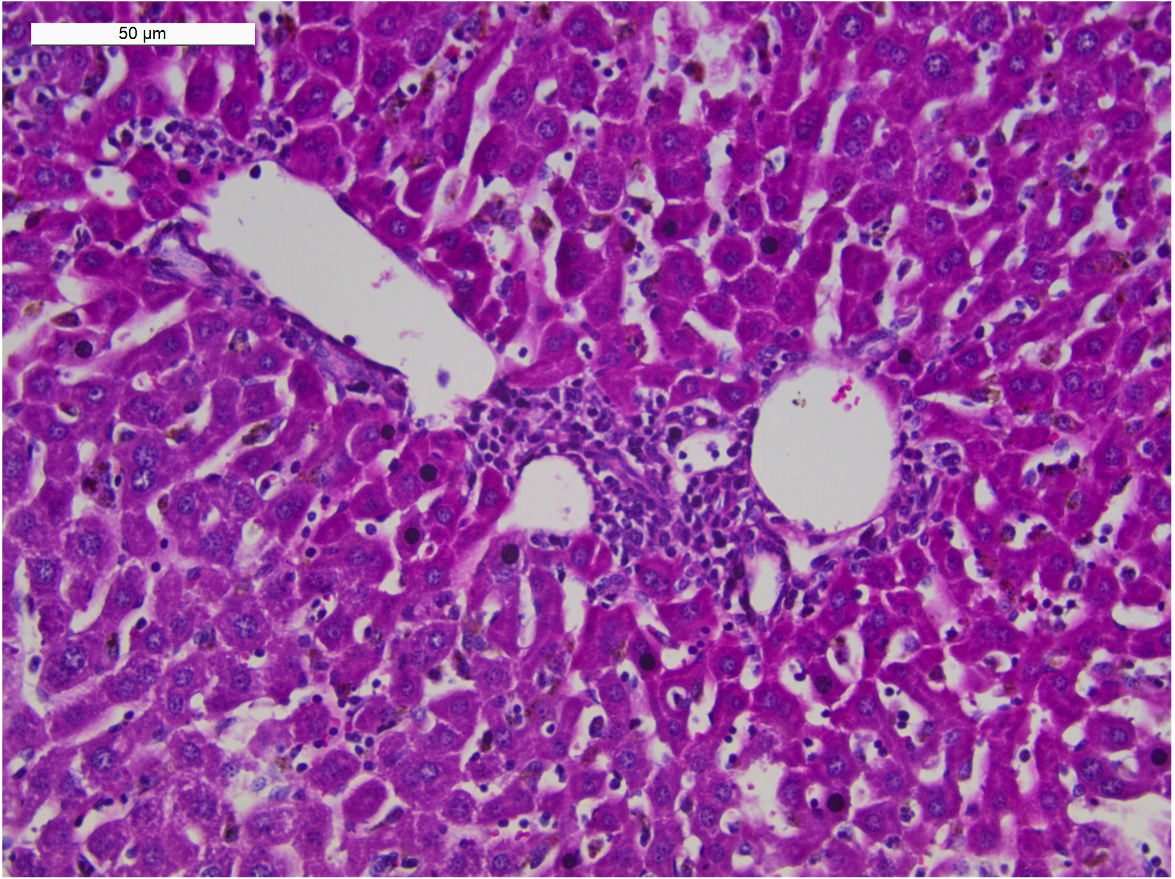

Supplement: Supplementary file 15 — EV and Appendix Figures Source Data [file 44321_2025_264_MOESM15_ESM.zip › EMM-2024-20682_SourceDataForExpandedViewAndAppendix/Sourcedata Appendix fig S3/Appendix S3_H&E_PCAS GRiKO6.png]

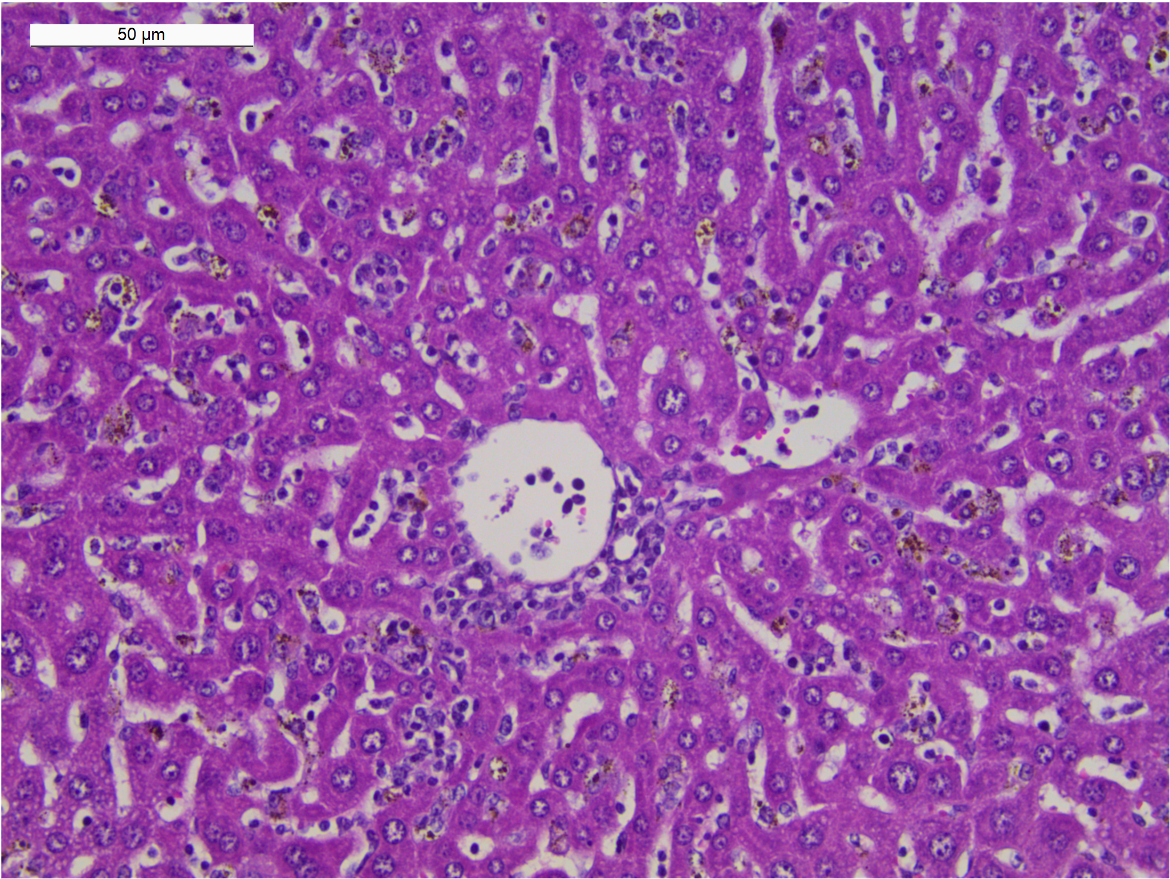

Supplement: Supplementary file 15 — EV and Appendix Figures Source Data [file 44321_2025_264_MOESM15_ESM.zip › EMM-2024-20682_SourceDataForExpandedViewAndAppendix/Sourcedata Appendix fig S3/Appendix S3_H&E_PCAS GRiKO7.png]

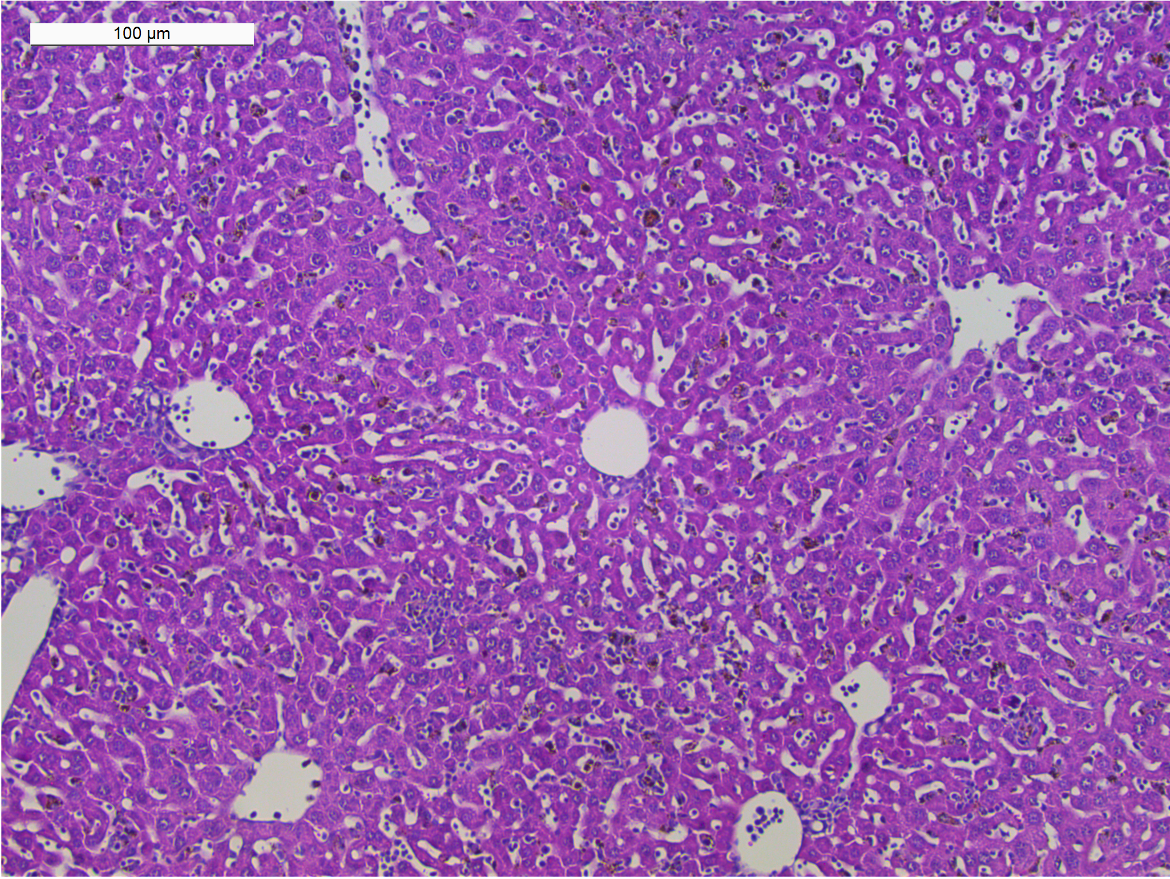

Supplement: Supplementary file 15 — EV and Appendix Figures Source Data [file 44321_2025_264_MOESM15_ESM.zip › EMM-2024-20682_SourceDataForExpandedViewAndAppendix/Sourcedata Appendix fig S3/Appendix S3_H&E_PCAS GRiKO8.png]

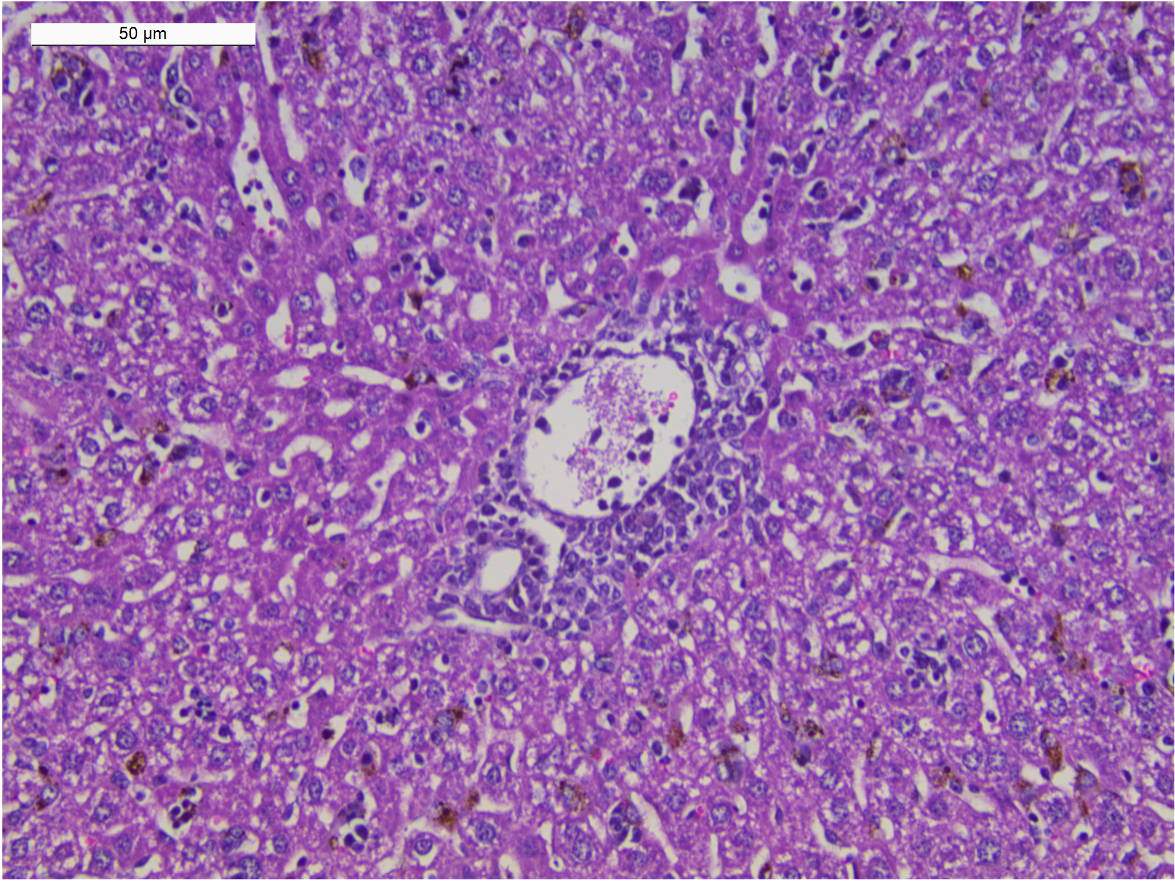

Supplement: Supplementary file 15 — EV and Appendix Figures Source Data [file 44321_2025_264_MOESM15_ESM.zip › EMM-2024-20682_SourceDataForExpandedViewAndAppendix/Sourcedata Appendix fig S3/Appendix S3_H&E_PcAS WT1.png]

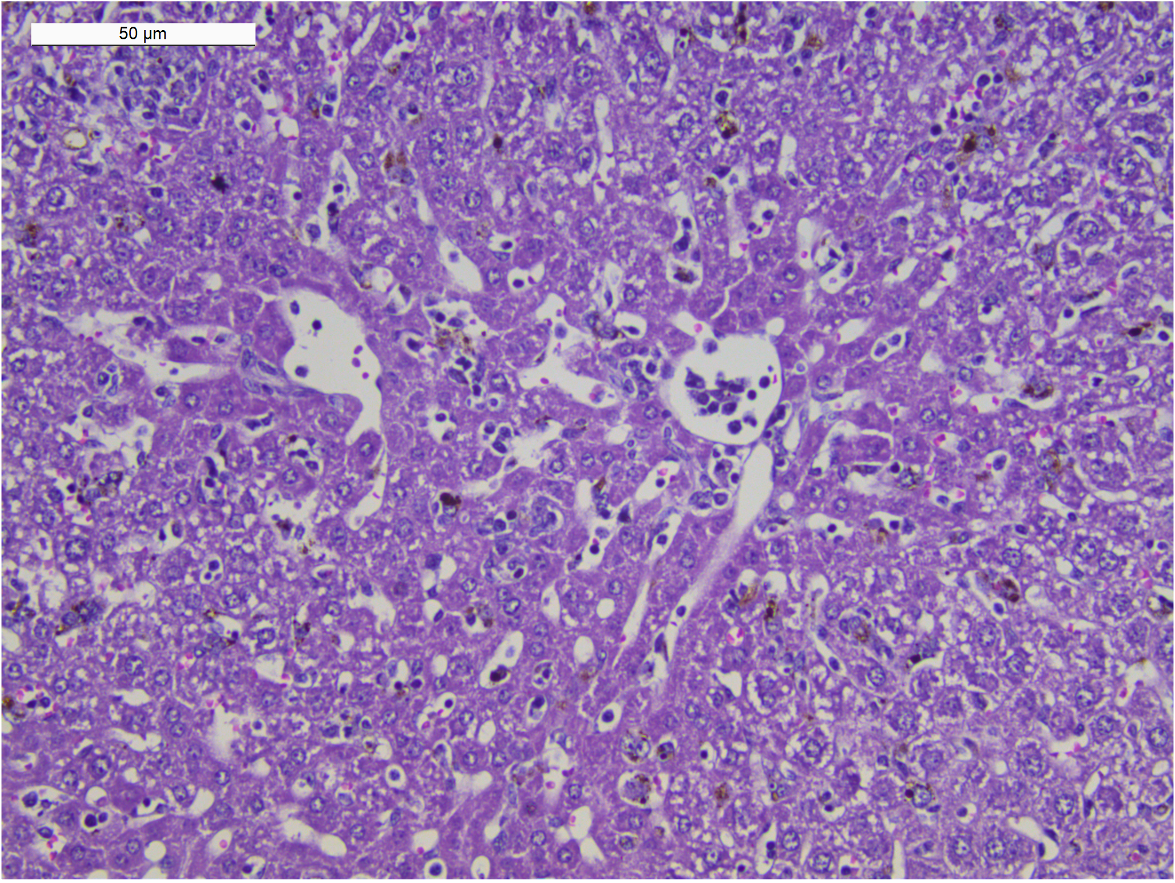

Supplement: Supplementary file 15 — EV and Appendix Figures Source Data [file 44321_2025_264_MOESM15_ESM.zip › EMM-2024-20682_SourceDataForExpandedViewAndAppendix/Sourcedata Appendix fig S3/Appendix S3_H&E_PcAS WT2.png]

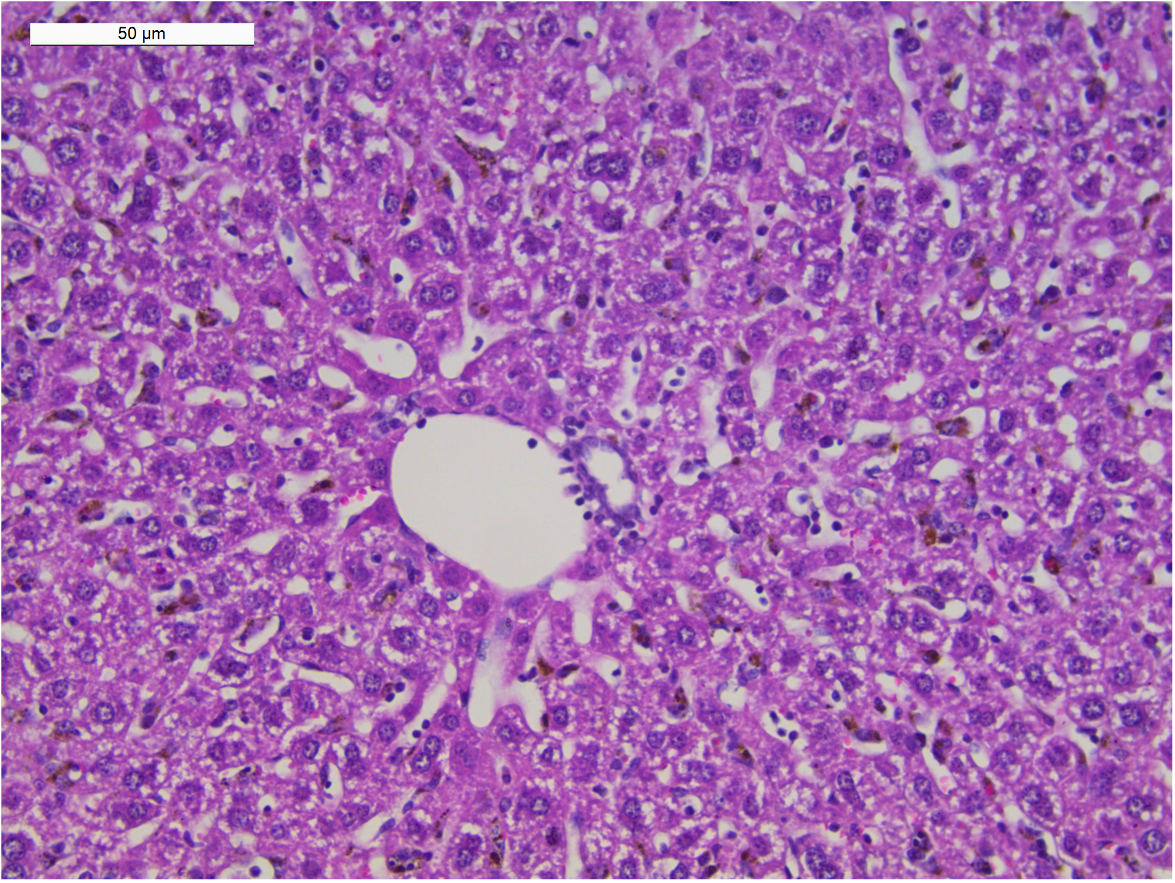

Supplement: Supplementary file 15 — EV and Appendix Figures Source Data [file 44321_2025_264_MOESM15_ESM.zip › EMM-2024-20682_SourceDataForExpandedViewAndAppendix/Sourcedata Appendix fig S3/Appendix S3_H&E_PcAS WT3.png]

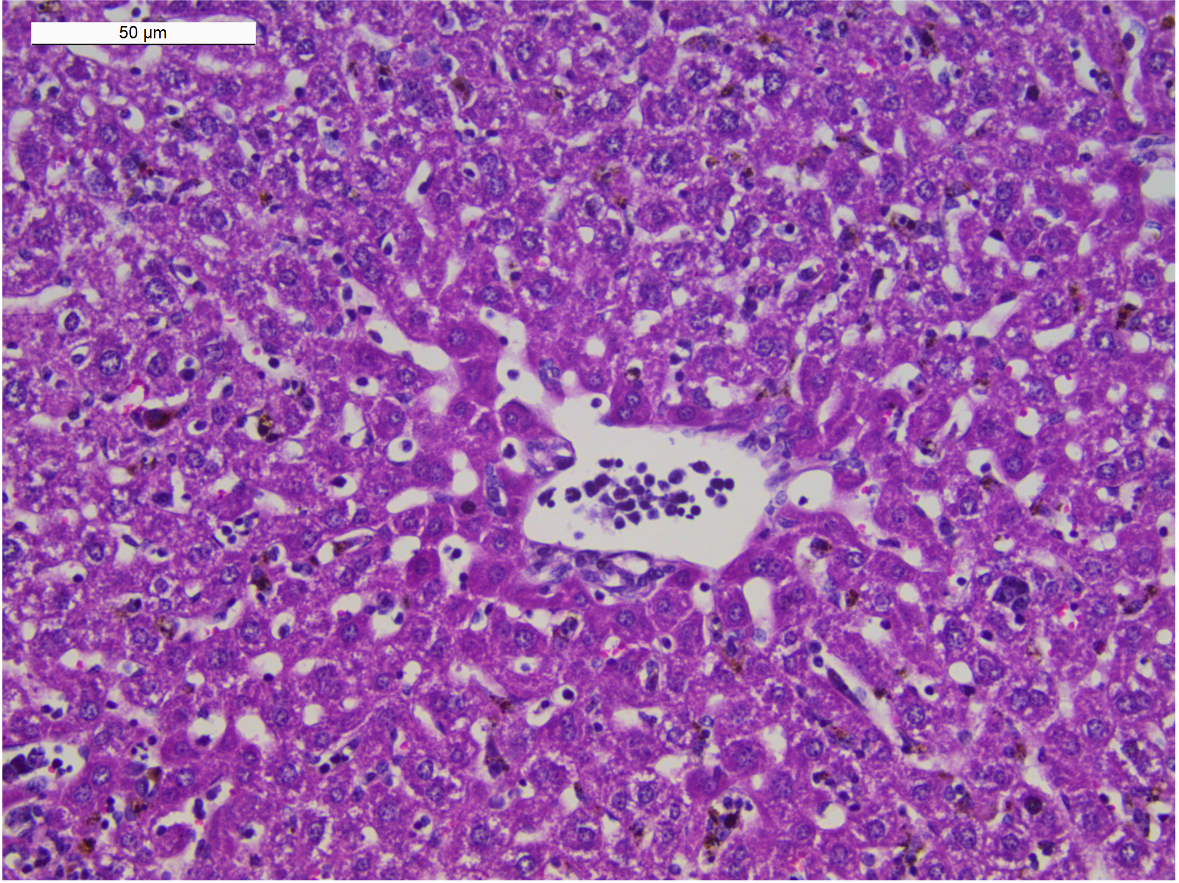

Supplement: Supplementary file 15 — EV and Appendix Figures Source Data [file 44321_2025_264_MOESM15_ESM.zip › EMM-2024-20682_SourceDataForExpandedViewAndAppendix/Sourcedata Appendix fig S3/Appendix S3_H&E_PcAS WT4.png]

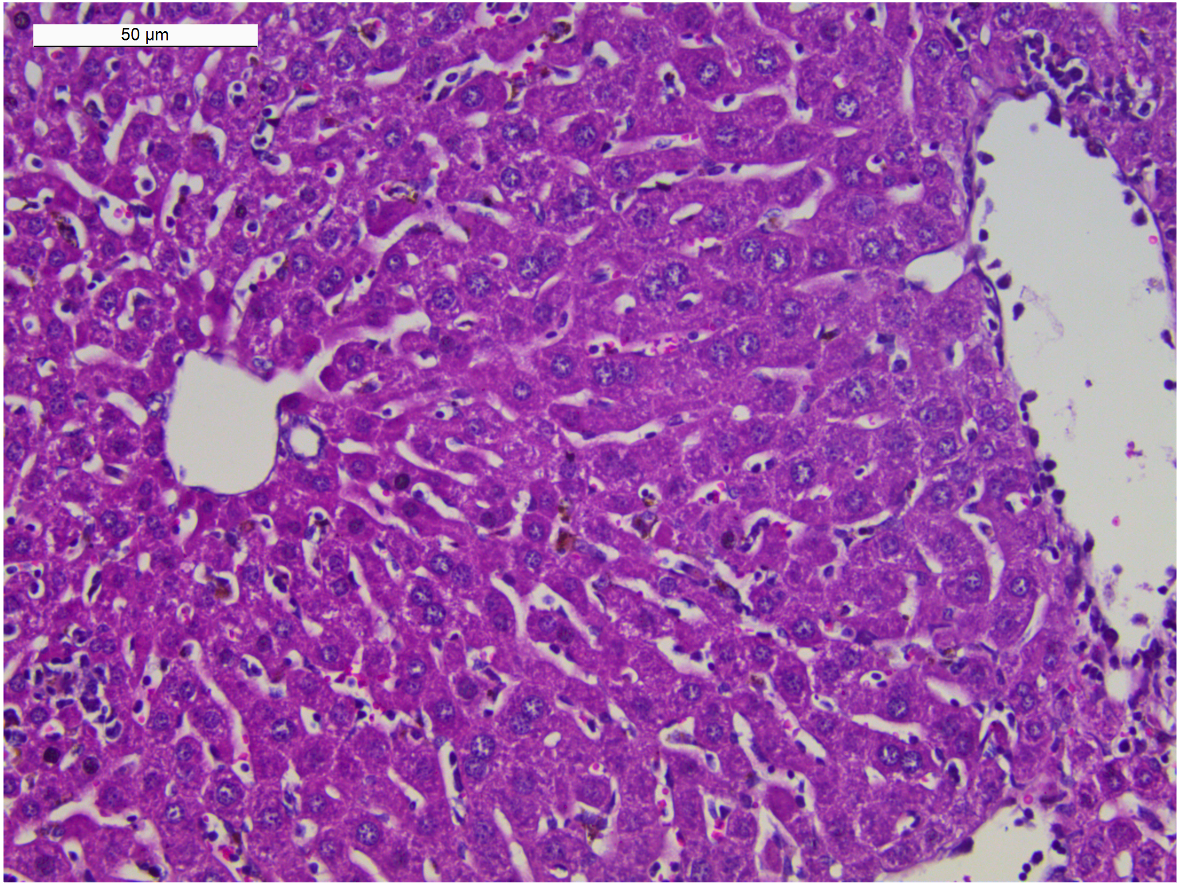

Supplement: Supplementary file 15 — EV and Appendix Figures Source Data [file 44321_2025_264_MOESM15_ESM.zip › EMM-2024-20682_SourceDataForExpandedViewAndAppendix/Sourcedata Appendix fig S3/Appendix S3_H&E_PcAS WT5.png]

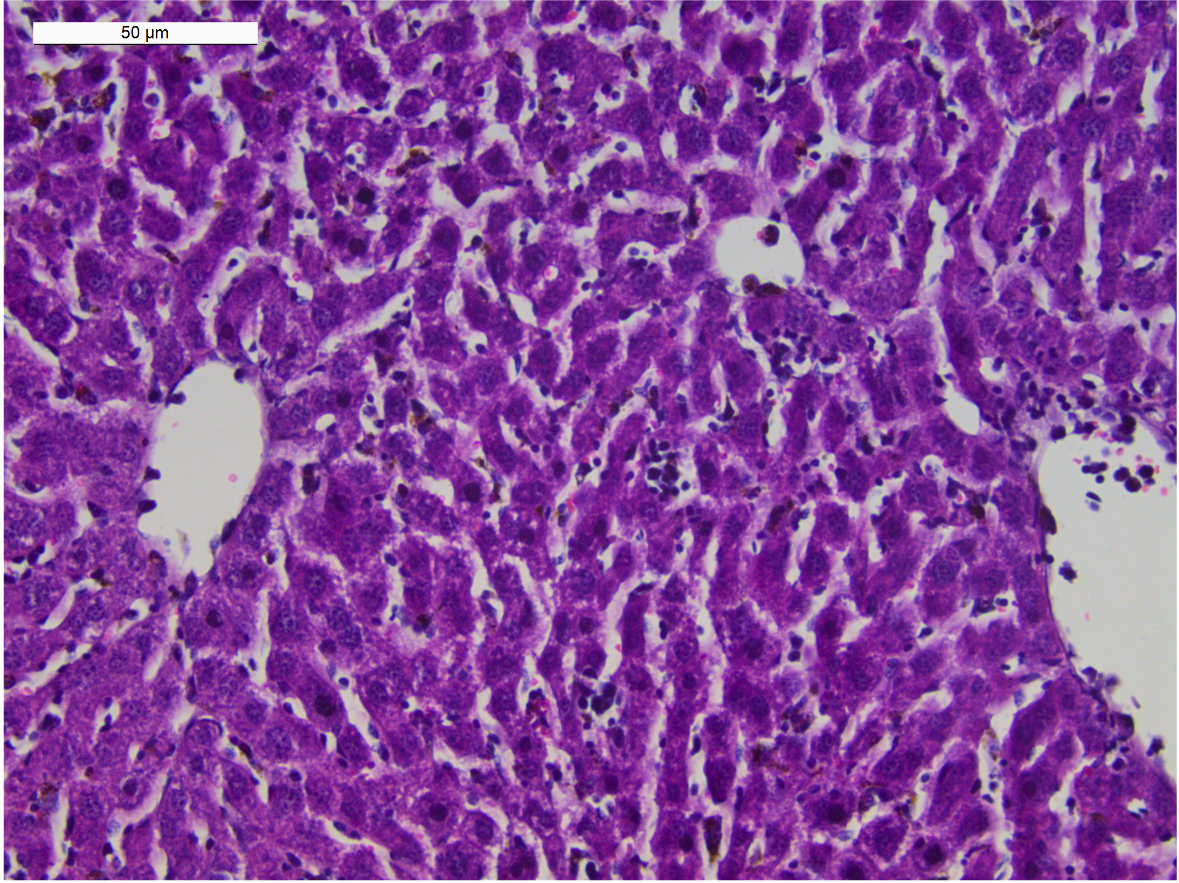

Supplement: Supplementary file 15 — EV and Appendix Figures Source Data [file 44321_2025_264_MOESM15_ESM.zip › EMM-2024-20682_SourceDataForExpandedViewAndAppendix/Sourcedata Appendix fig S3/Appendix S3_H&E_PcAS WT6.png]

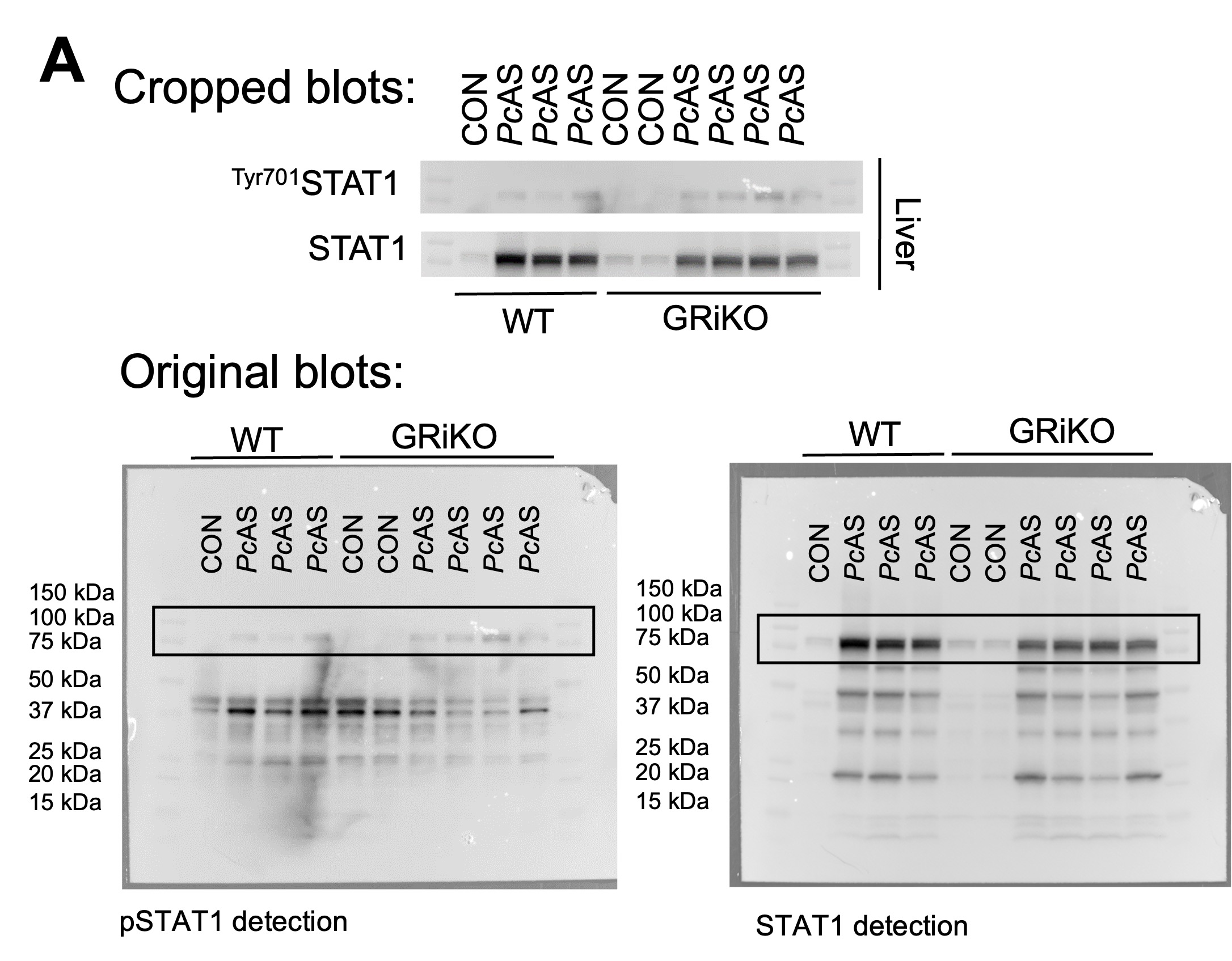

Supplement: Supplementary file 15 — EV and Appendix Figures Source Data [file 44321_2025_264_MOESM15_ESM.zip › EMM-2024-20682_SourceDataForExpandedViewAndAppendix/Sourcedata Appendix fig S9/Appendix S9A/Appendix S9A_Cropped western blot image.jpg]

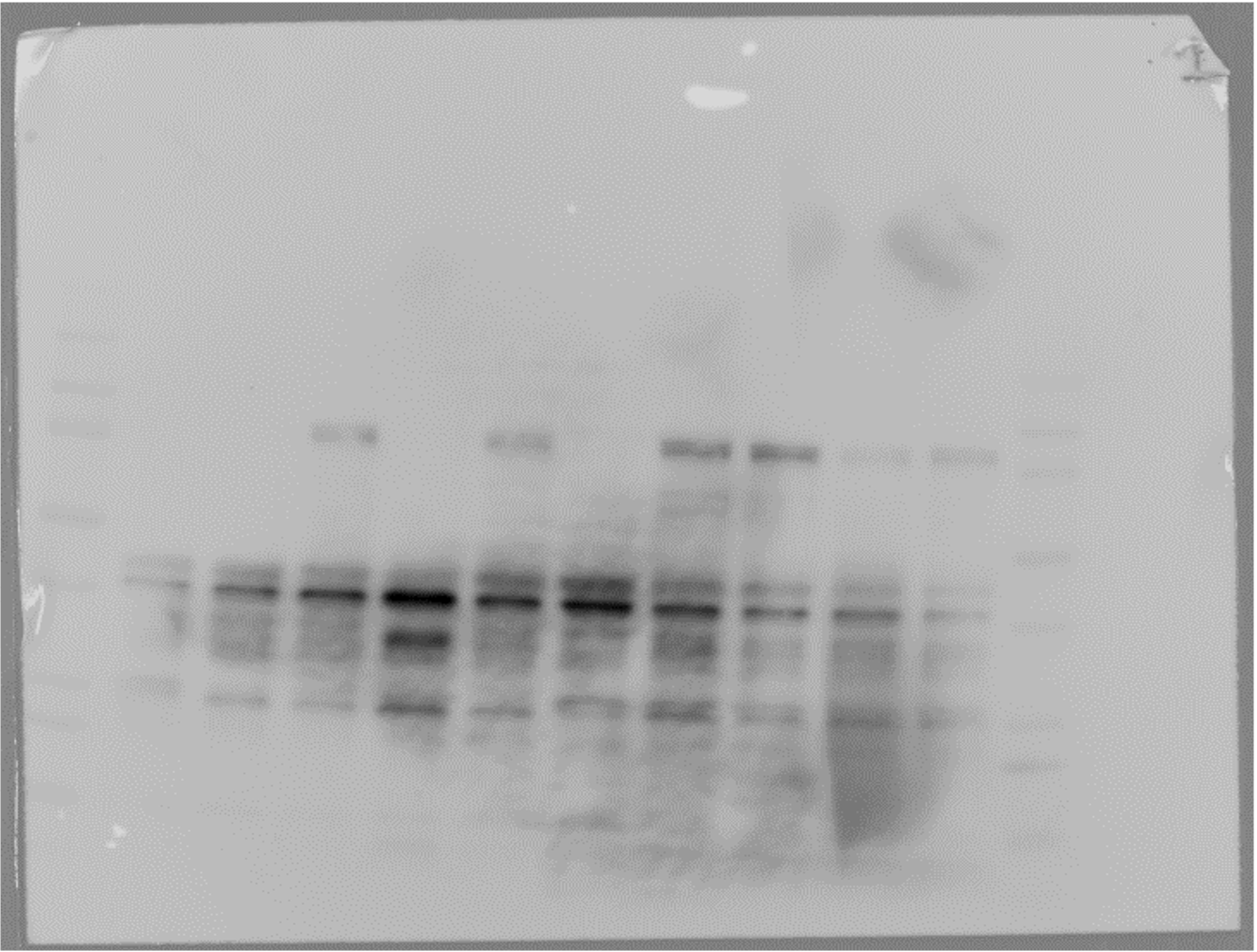

Supplement: Supplementary file 15 — EV and Appendix Figures Source Data [file 44321_2025_264_MOESM15_ESM.zip › EMM-2024-20682_SourceDataForExpandedViewAndAppendix/Sourcedata Appendix fig S9/Appendix S9A/Appendix S9A_Western blot_pSTAT1_gel1.png]

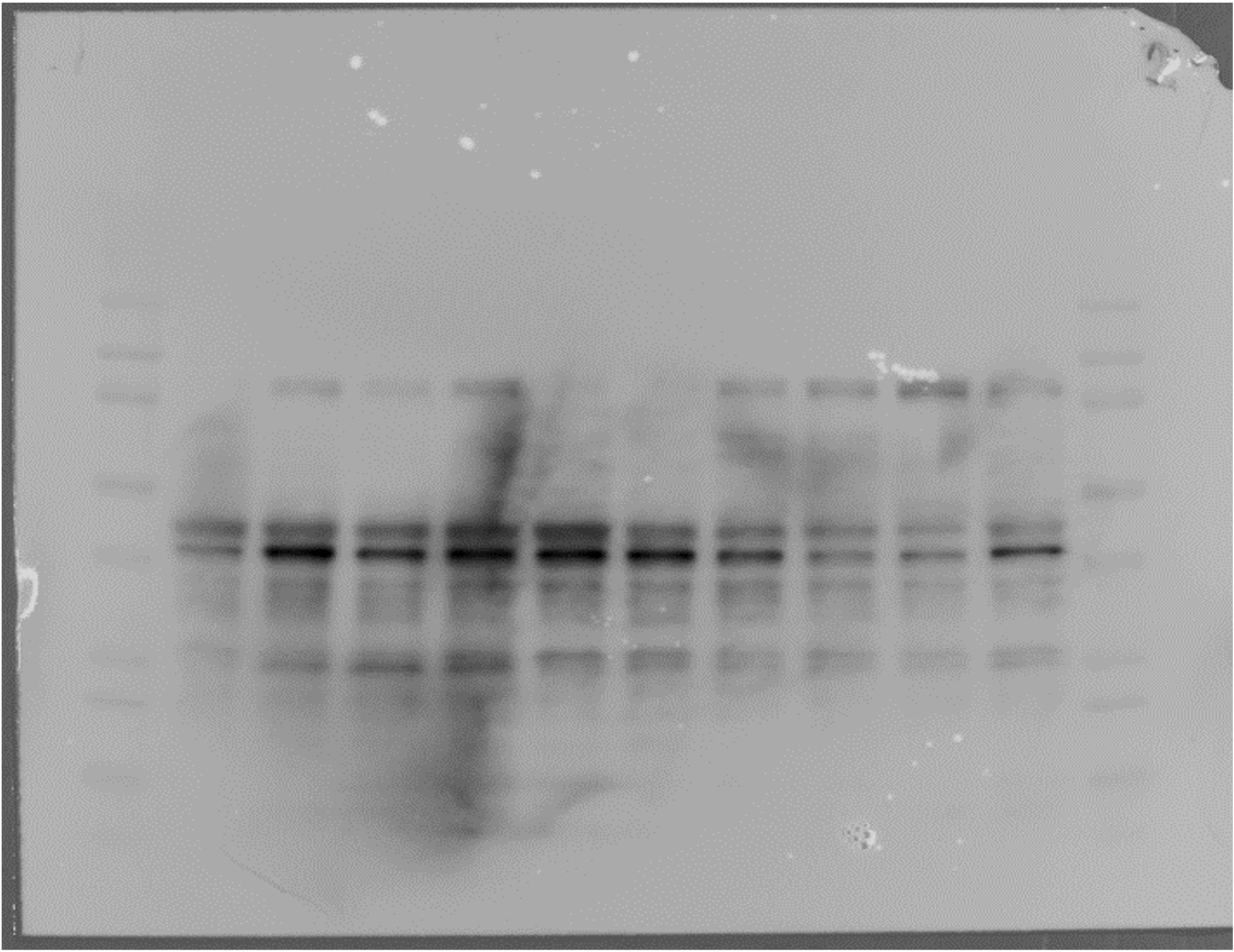

Supplement: Supplementary file 15 — EV and Appendix Figures Source Data [file 44321_2025_264_MOESM15_ESM.zip › EMM-2024-20682_SourceDataForExpandedViewAndAppendix/Sourcedata Appendix fig S9/Appendix S9A/Appendix S9A_Western blot_pSTAT1_gel2.png]

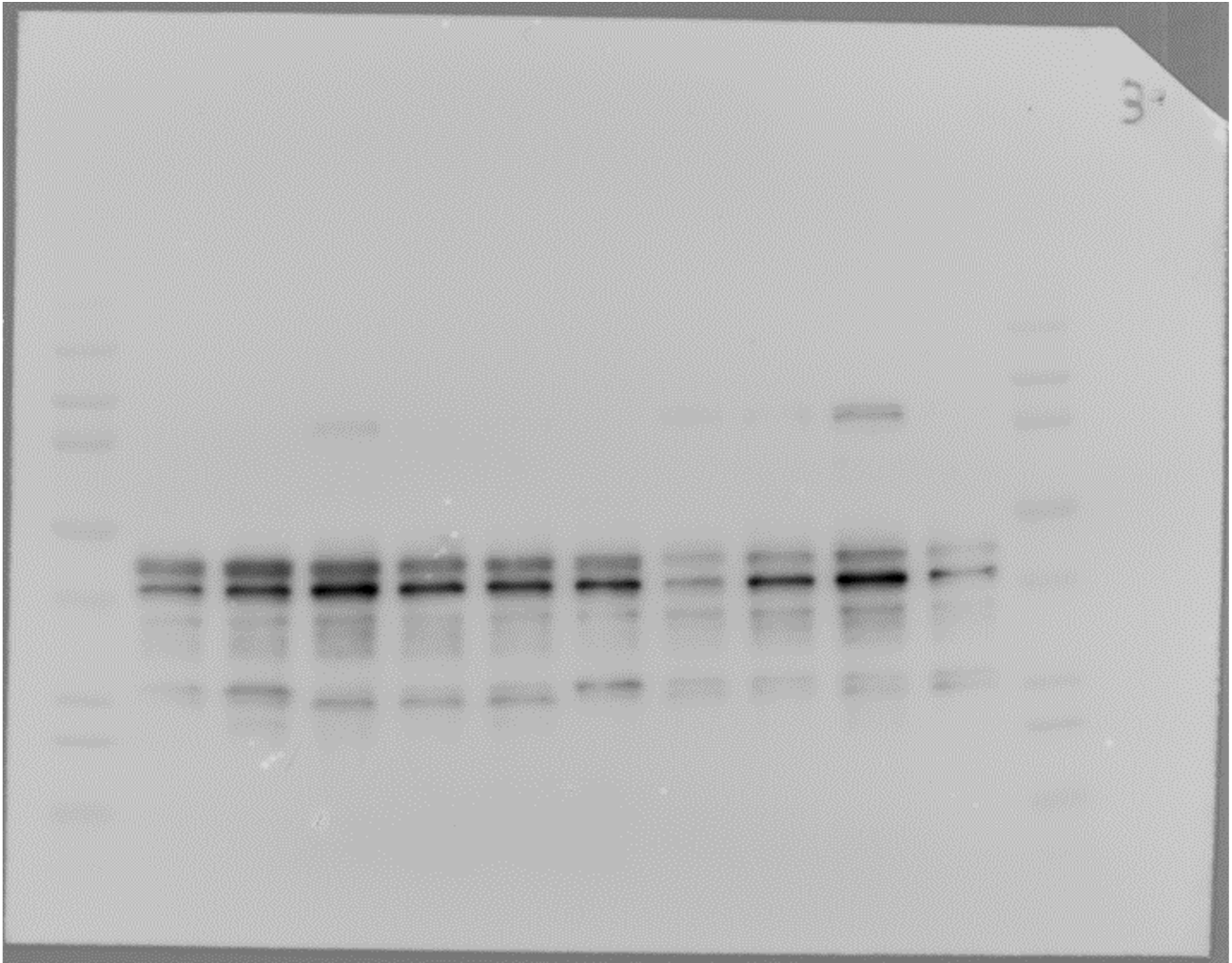

Supplement: Supplementary file 15 — EV and Appendix Figures Source Data [file 44321_2025_264_MOESM15_ESM.zip › EMM-2024-20682_SourceDataForExpandedViewAndAppendix/Sourcedata Appendix fig S9/Appendix S9A/Appendix S9A_Western blot_pSTAT1_gel3.png]

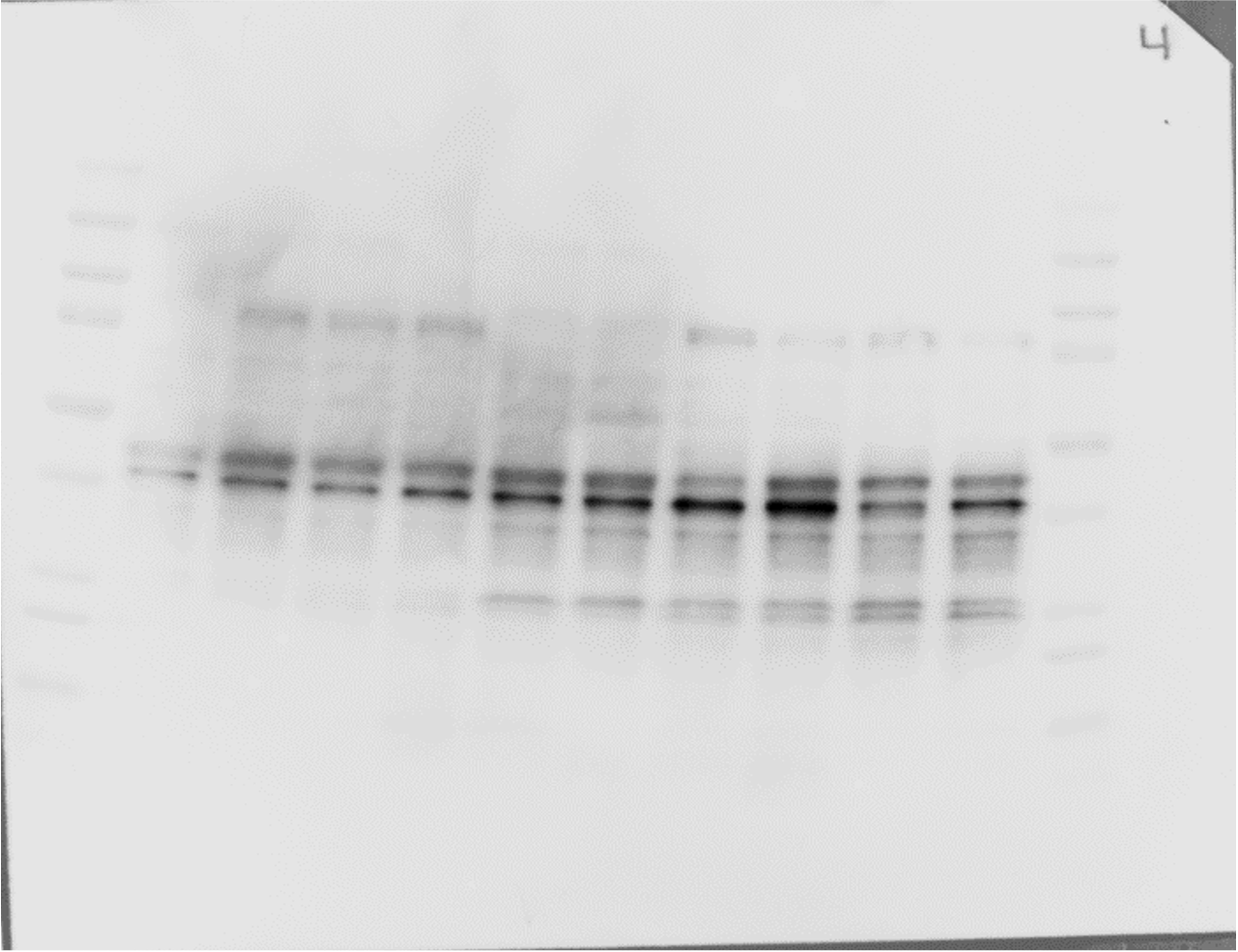

Supplement: Supplementary file 15 — EV and Appendix Figures Source Data [file 44321_2025_264_MOESM15_ESM.zip › EMM-2024-20682_SourceDataForExpandedViewAndAppendix/Sourcedata Appendix fig S9/Appendix S9A/Appendix S9A_Western blot_pSTAT1_gel4.png]

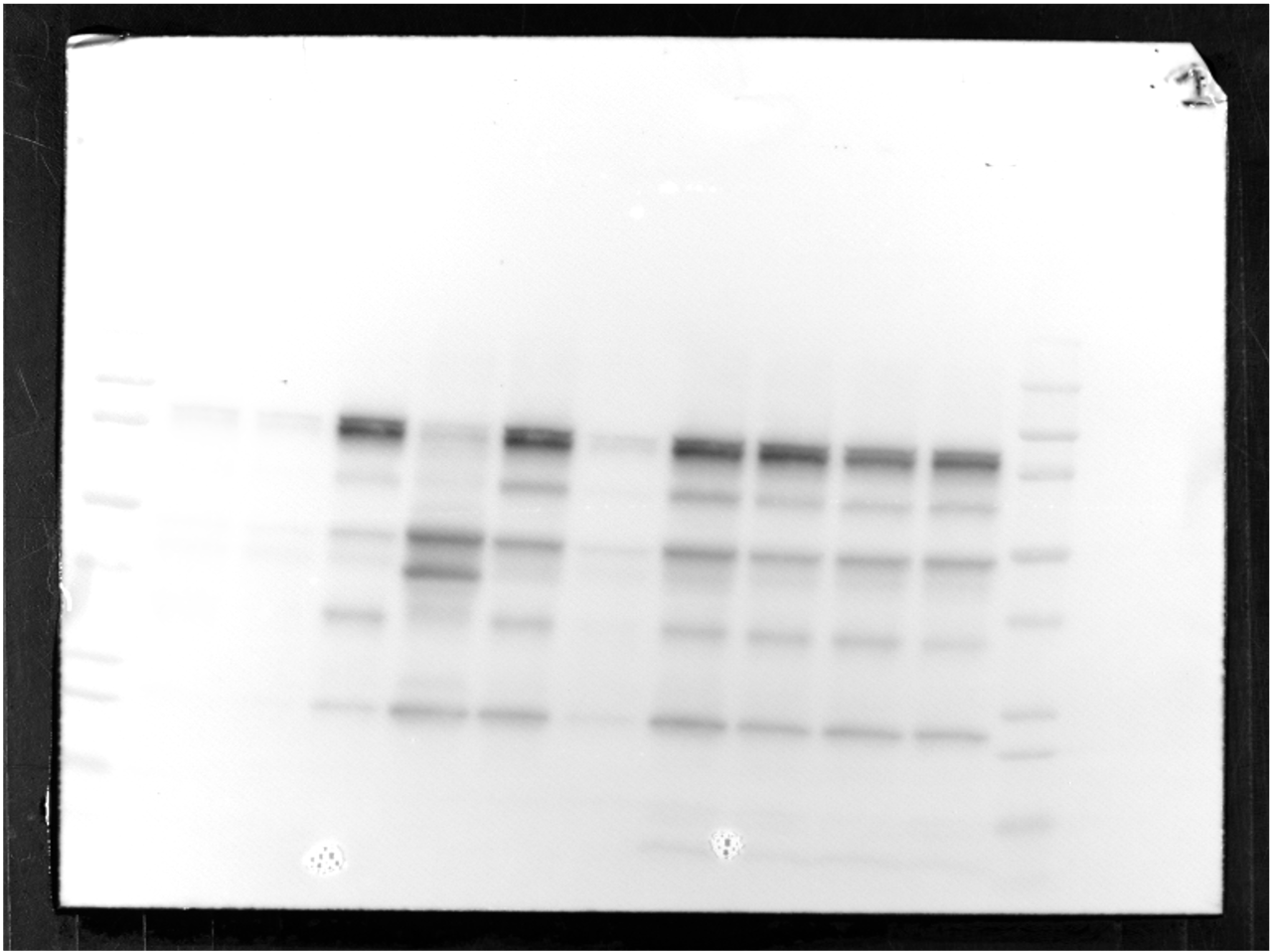

Supplement: Supplementary file 15 — EV and Appendix Figures Source Data [file 44321_2025_264_MOESM15_ESM.zip › EMM-2024-20682_SourceDataForExpandedViewAndAppendix/Sourcedata Appendix fig S9/Appendix S9A/Appendix S9A_Western blot_STAT1_gel1.png]

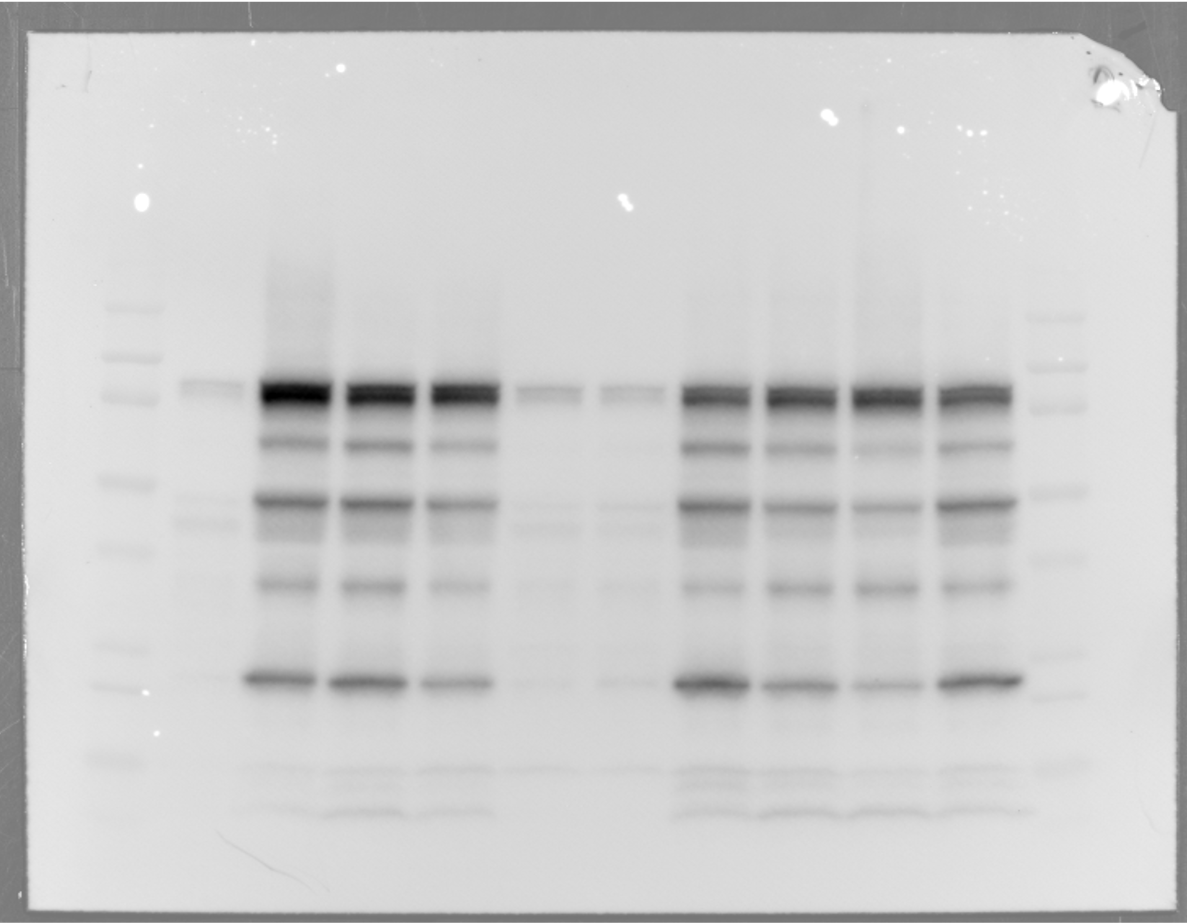

Supplement: Supplementary file 15 — EV and Appendix Figures Source Data [file 44321_2025_264_MOESM15_ESM.zip › EMM-2024-20682_SourceDataForExpandedViewAndAppendix/Sourcedata Appendix fig S9/Appendix S9A/Appendix S9A_Western blot_STAT1_gel2.png]

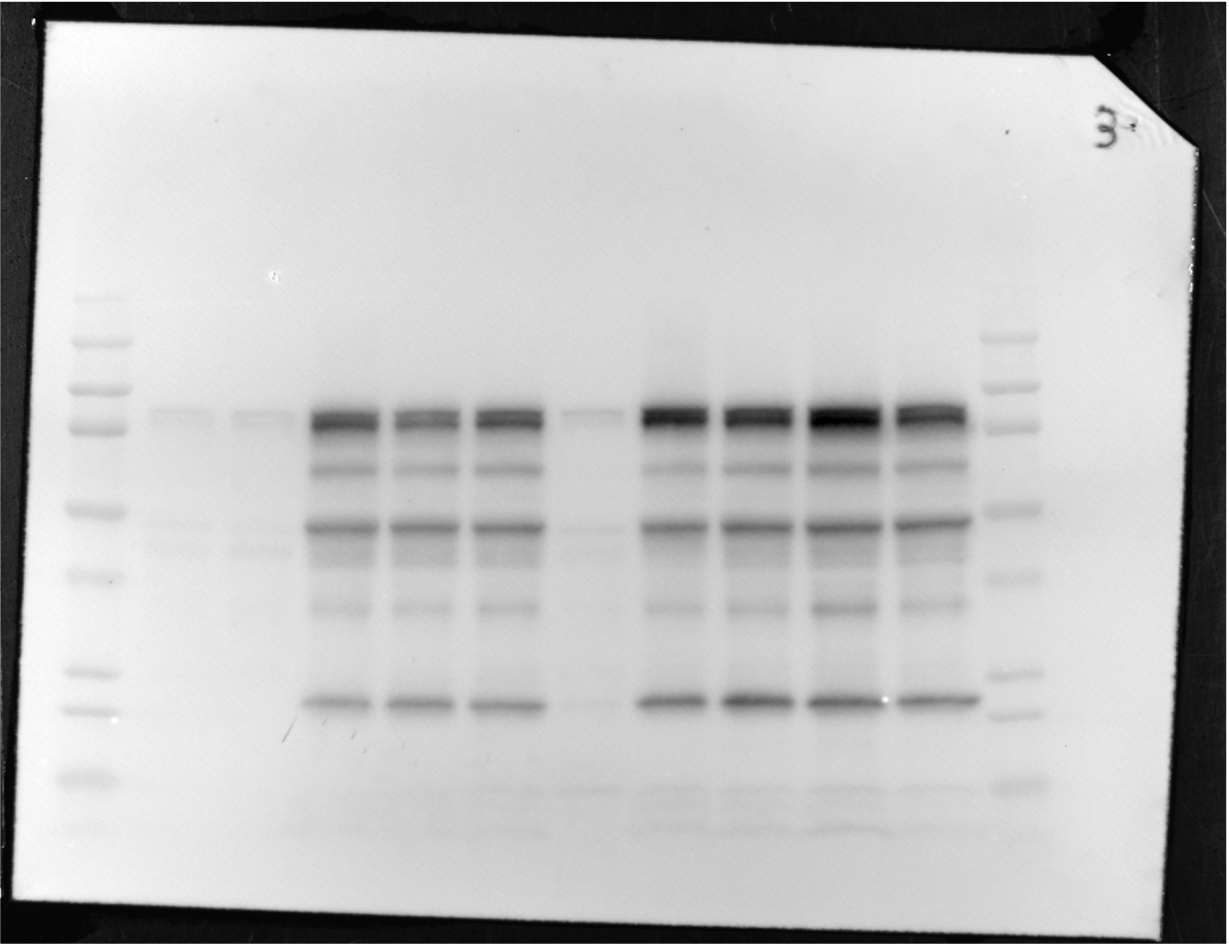

Supplement: Supplementary file 15 — EV and Appendix Figures Source Data [file 44321_2025_264_MOESM15_ESM.zip › EMM-2024-20682_SourceDataForExpandedViewAndAppendix/Sourcedata Appendix fig S9/Appendix S9A/Appendix S9A_Western blot_STAT1_gel3.png]

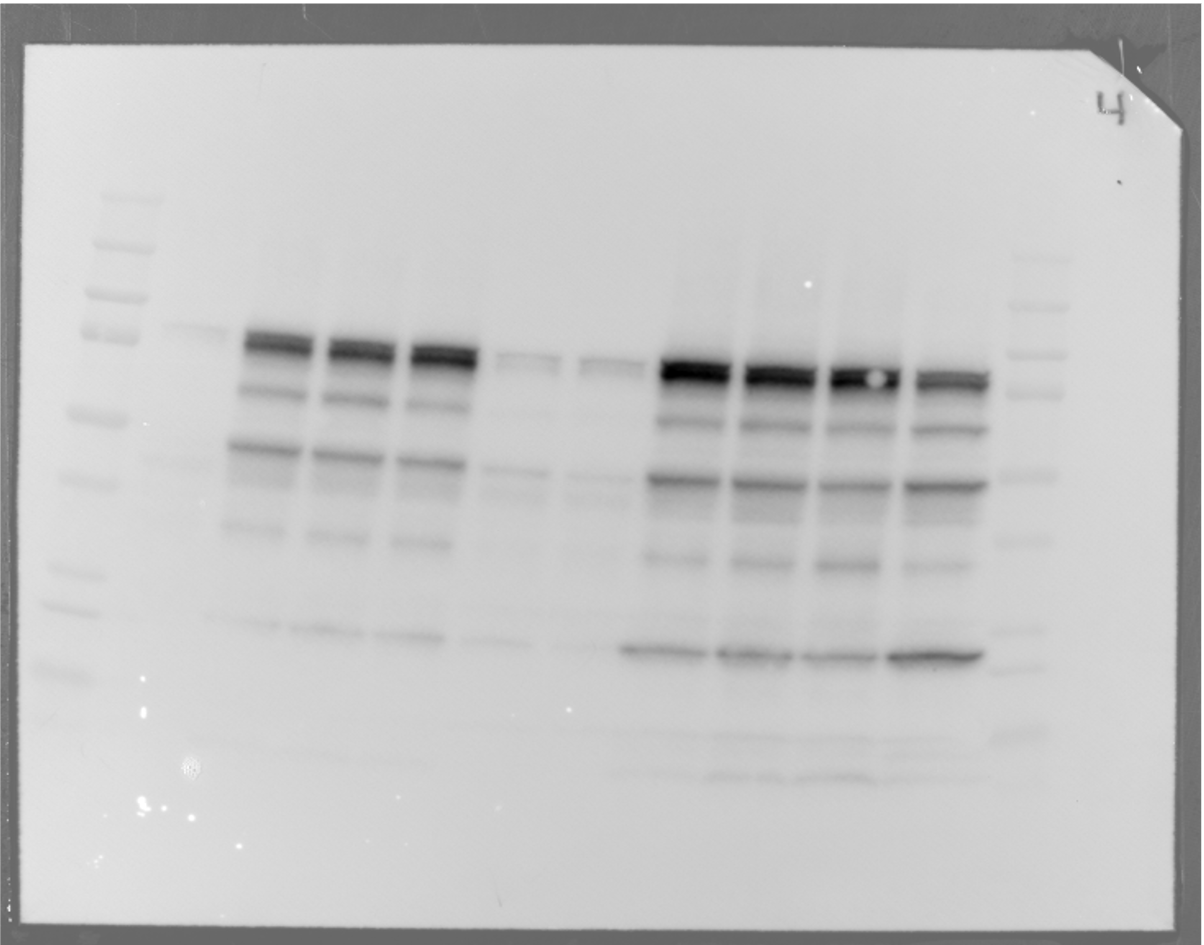

Supplement: Supplementary file 15 — EV and Appendix Figures Source Data [file 44321_2025_264_MOESM15_ESM.zip › EMM-2024-20682_SourceDataForExpandedViewAndAppendix/Sourcedata Appendix fig S9/Appendix S9A/Appendix S9A_Western blot_STAT1_gel4.png]

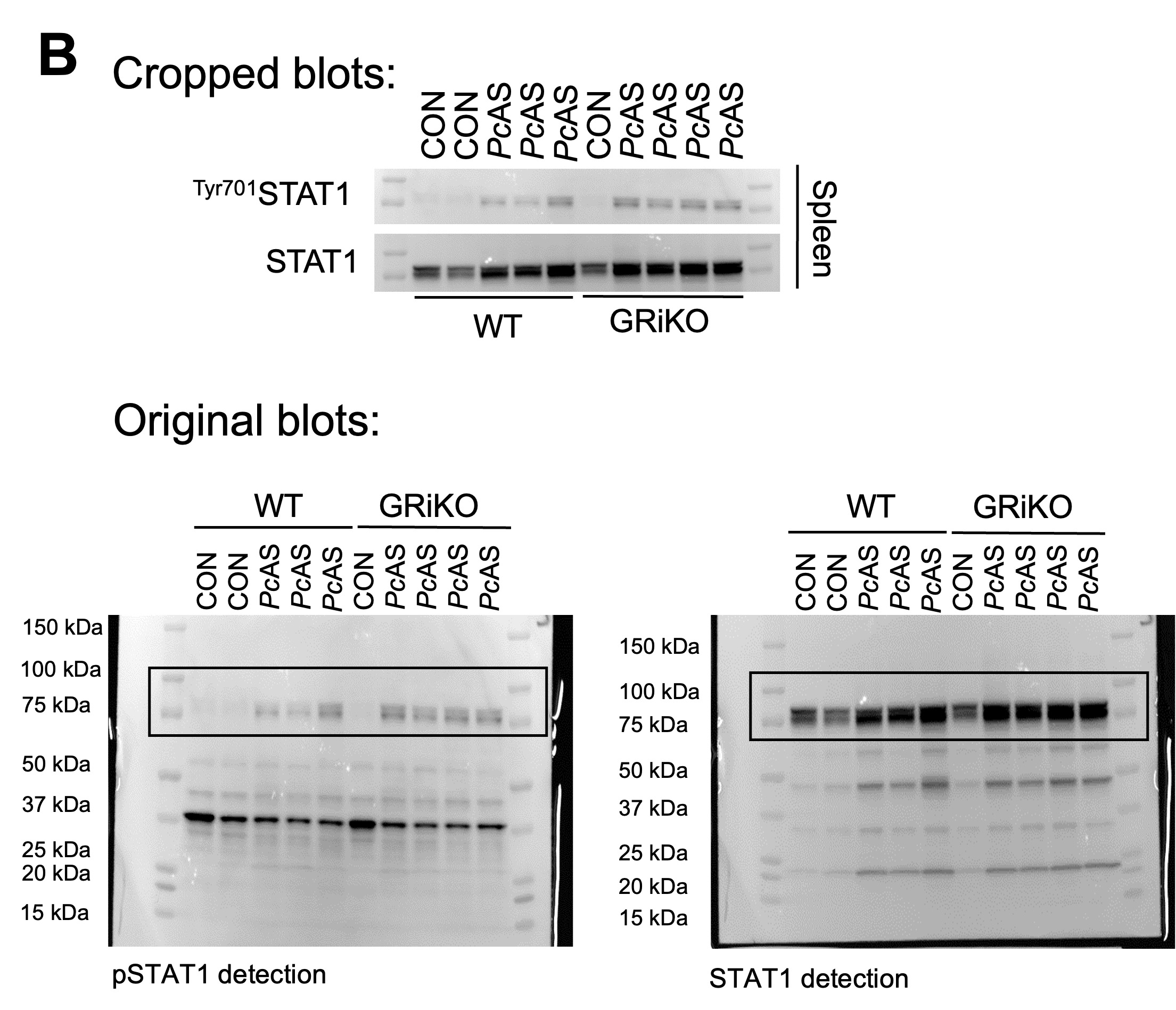

Supplement: Supplementary file 15 — EV and Appendix Figures Source Data [file 44321_2025_264_MOESM15_ESM.zip › EMM-2024-20682_SourceDataForExpandedViewAndAppendix/Sourcedata Appendix fig S9/Appendix S9B/Appendix S9B_Cropped western blot image.jpg]

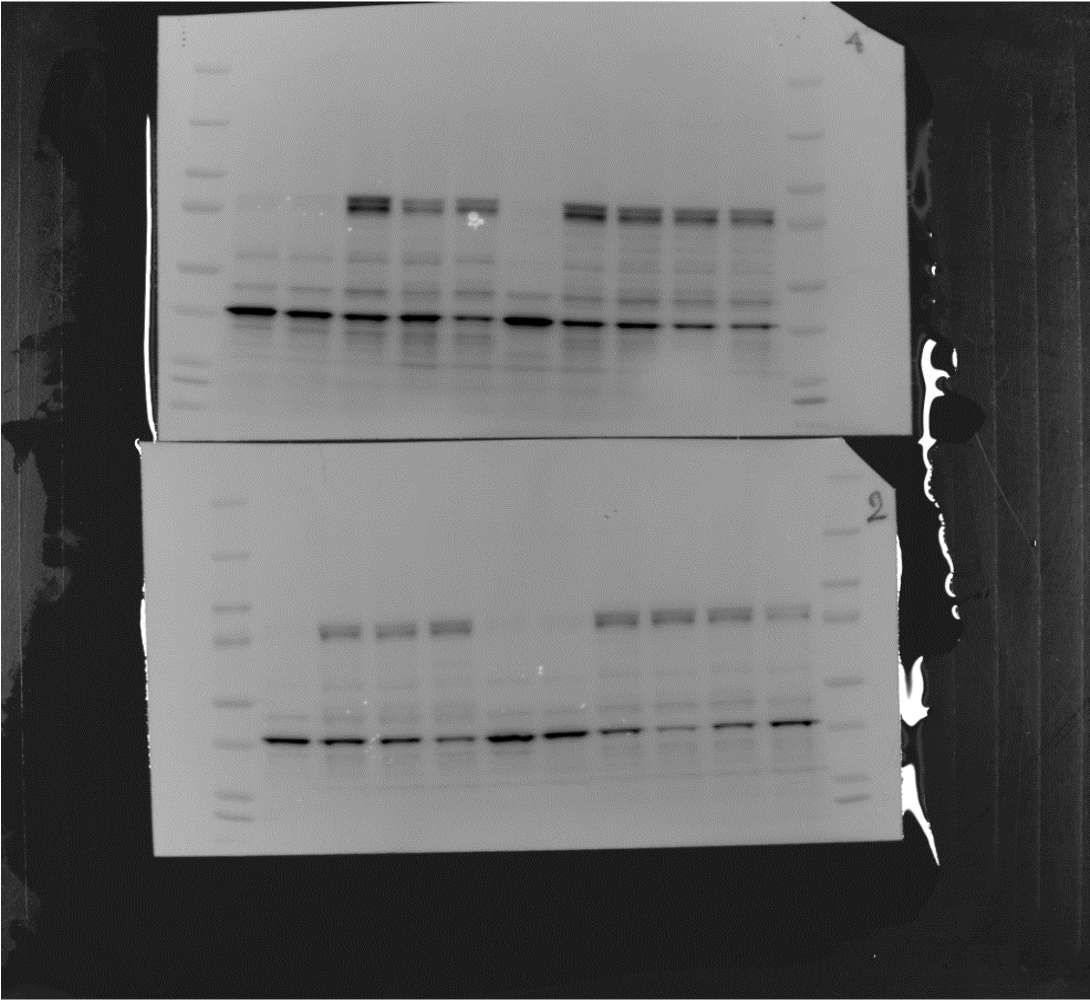

Supplement: Supplementary file 15 — EV and Appendix Figures Source Data [file 44321_2025_264_MOESM15_ESM.zip › EMM-2024-20682_SourceDataForExpandedViewAndAppendix/Sourcedata Appendix fig S9/Appendix S9B/Appendix S9B_Western blot_pSTAT1_gel1+2.png]

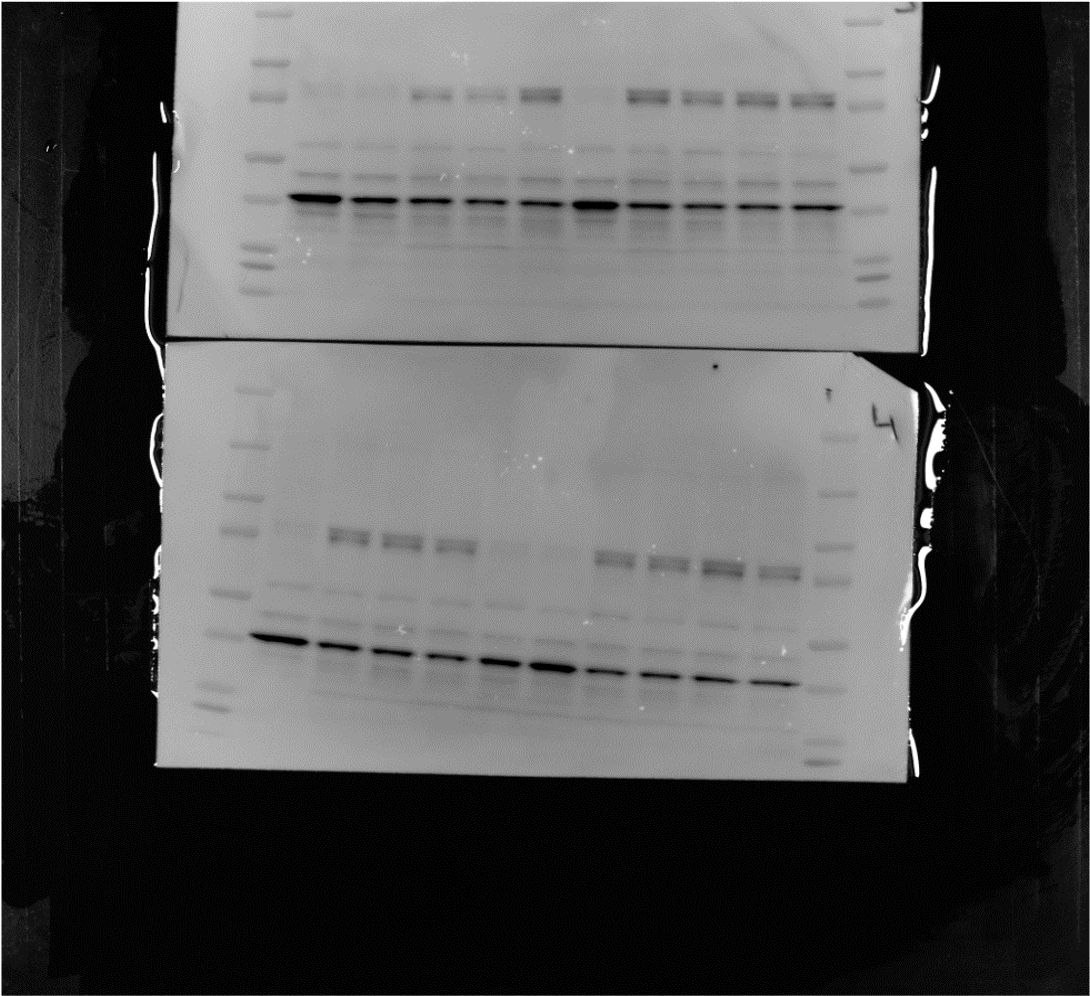

Supplement: Supplementary file 15 — EV and Appendix Figures Source Data [file 44321_2025_264_MOESM15_ESM.zip › EMM-2024-20682_SourceDataForExpandedViewAndAppendix/Sourcedata Appendix fig S9/Appendix S9B/Appendix S9B_Western blot_pSTAT1_gel3+4.png]

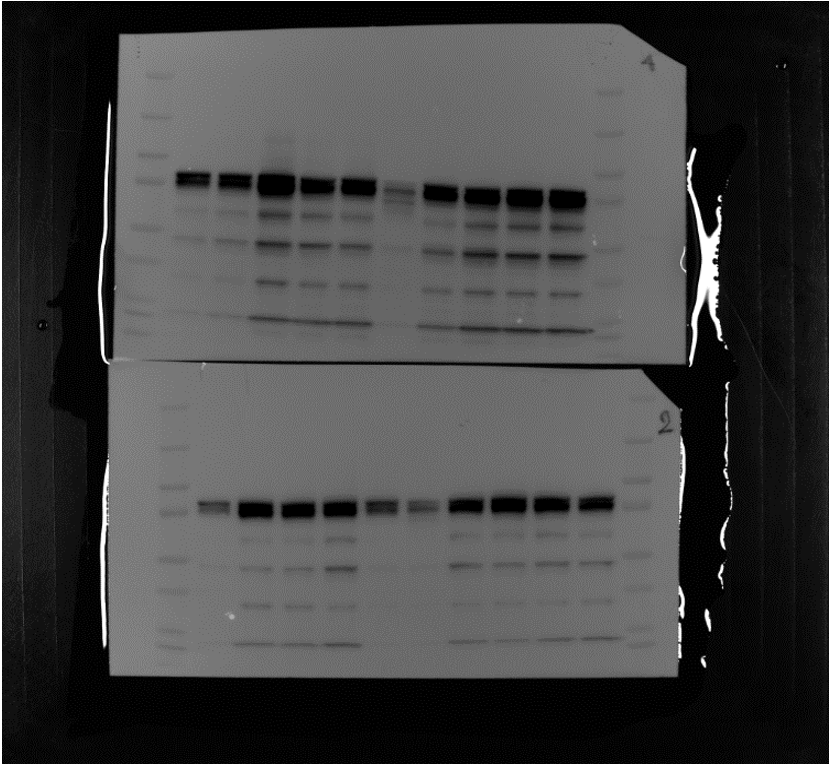

Supplement: Supplementary file 15 — EV and Appendix Figures Source Data [file 44321_2025_264_MOESM15_ESM.zip › EMM-2024-20682_SourceDataForExpandedViewAndAppendix/Sourcedata Appendix fig S9/Appendix S9B/Appendix S9B_Western blot_STAT1_gel1+2.png]

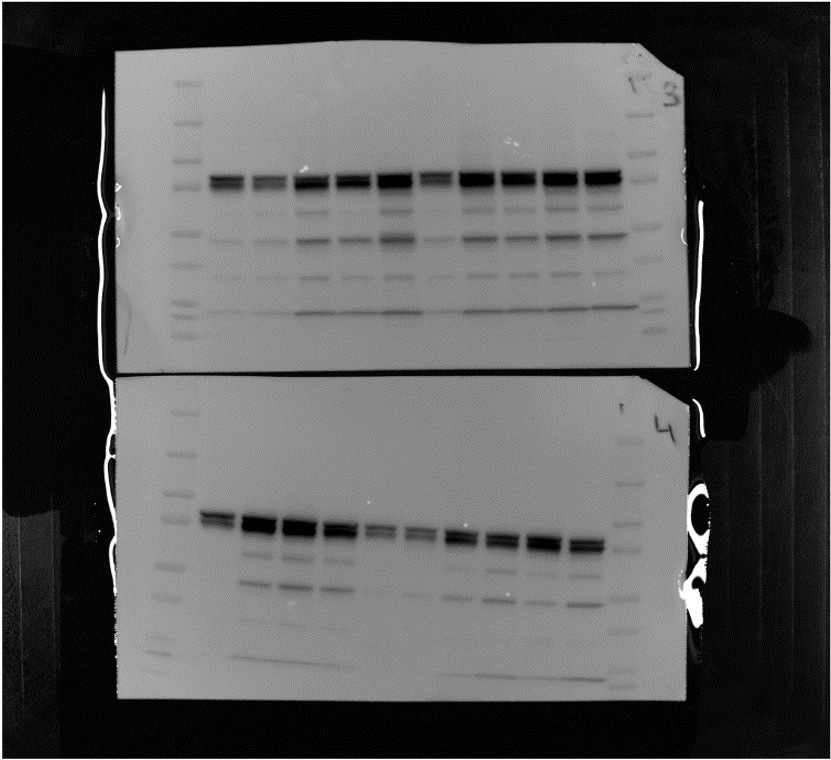

Supplement: Supplementary file 15 — EV and Appendix Figures Source Data [file 44321_2025_264_MOESM15_ESM.zip › EMM-2024-20682_SourceDataForExpandedViewAndAppendix/Sourcedata Appendix fig S9/Appendix S9B/Appendix S9B_Western blot_STAT1_gel3+4.png]

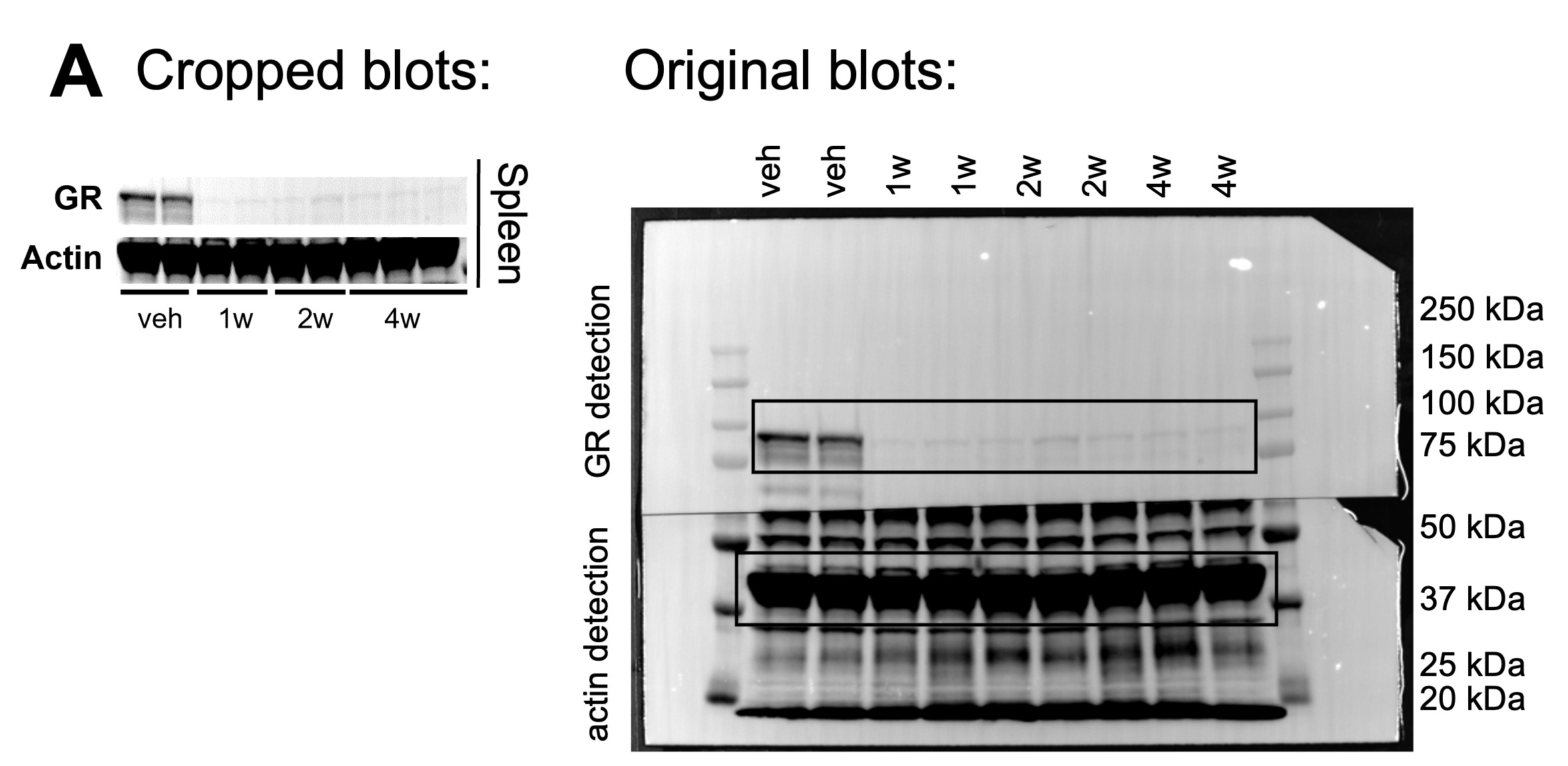

Supplement: Supplementary file 15 — EV and Appendix Figures Source Data [file 44321_2025_264_MOESM15_ESM.zip › EMM-2024-20682_SourceDataForExpandedViewAndAppendix/Sourcedata fig EV1/EV1A/EV1A_Cropped western blot image.jpg]

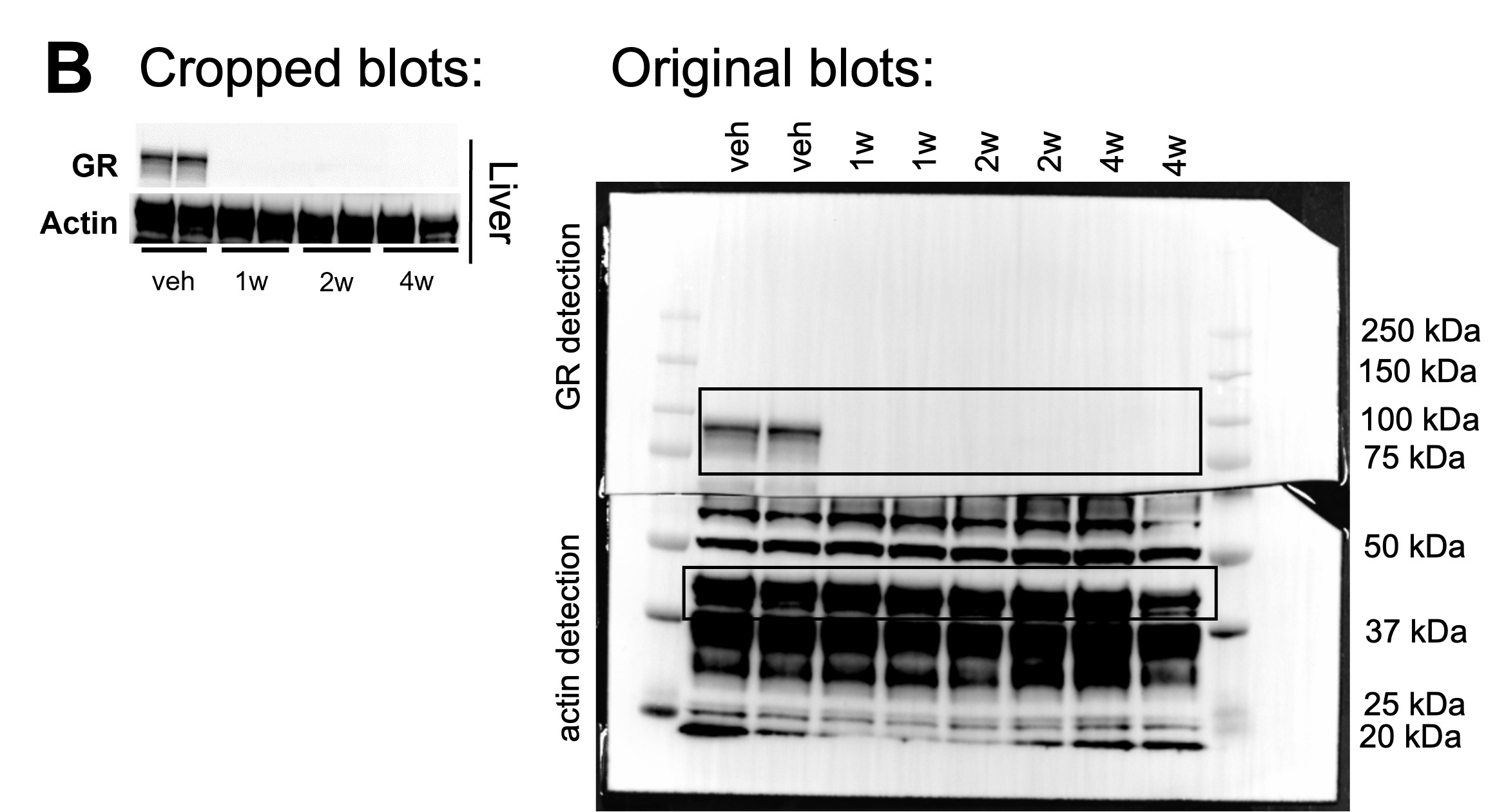

Supplement: Supplementary file 15 — EV and Appendix Figures Source Data [file 44321_2025_264_MOESM15_ESM.zip › EMM-2024-20682_SourceDataForExpandedViewAndAppendix/Sourcedata fig EV1/EV1B/EV1B_Cropped western blot image.jpg]

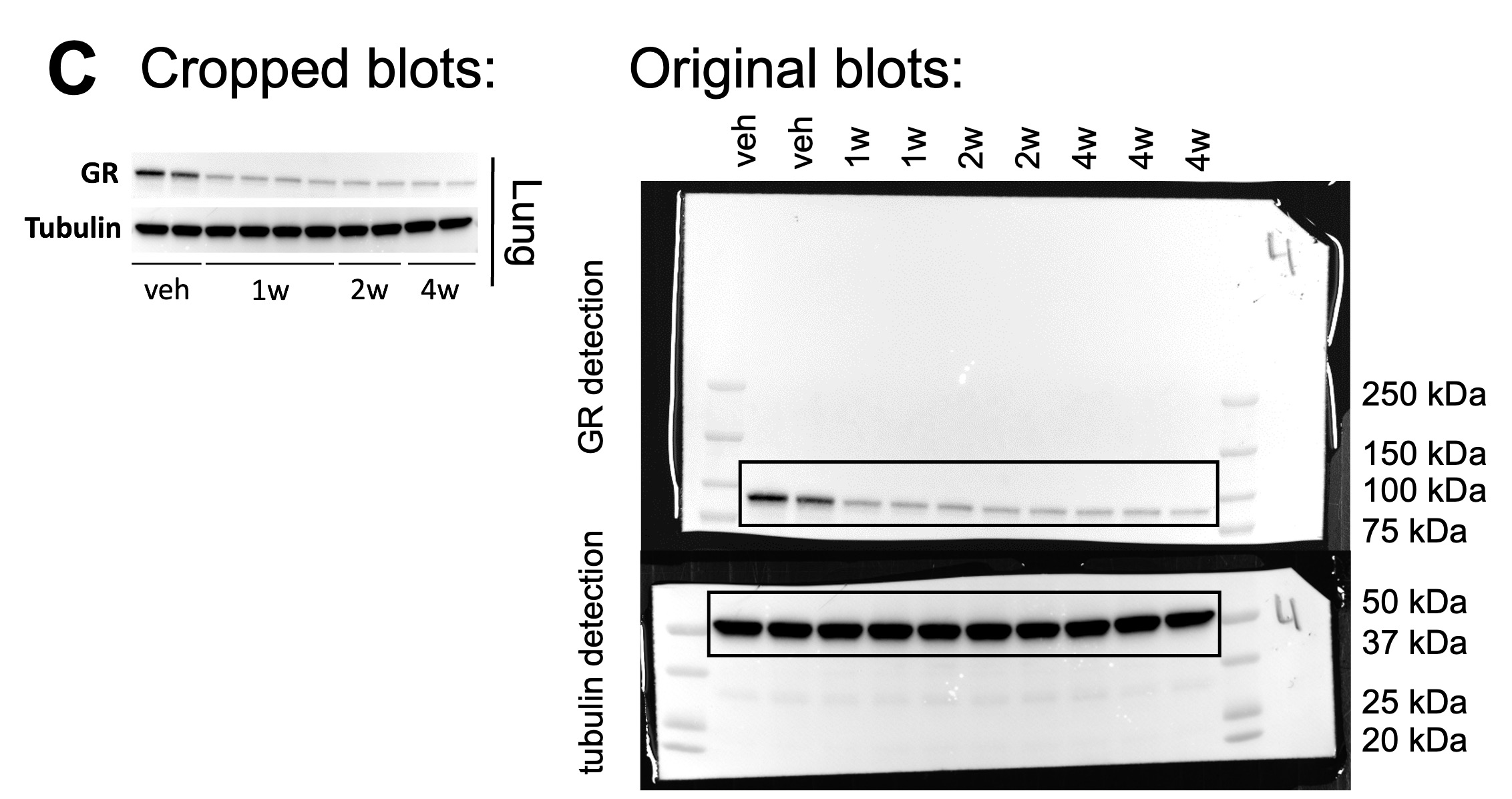

Supplement: Supplementary file 15 — EV and Appendix Figures Source Data [file 44321_2025_264_MOESM15_ESM.zip › EMM-2024-20682_SourceDataForExpandedViewAndAppendix/Sourcedata fig EV1/EV1C/EV1C_Cropped western blot image.jpg]

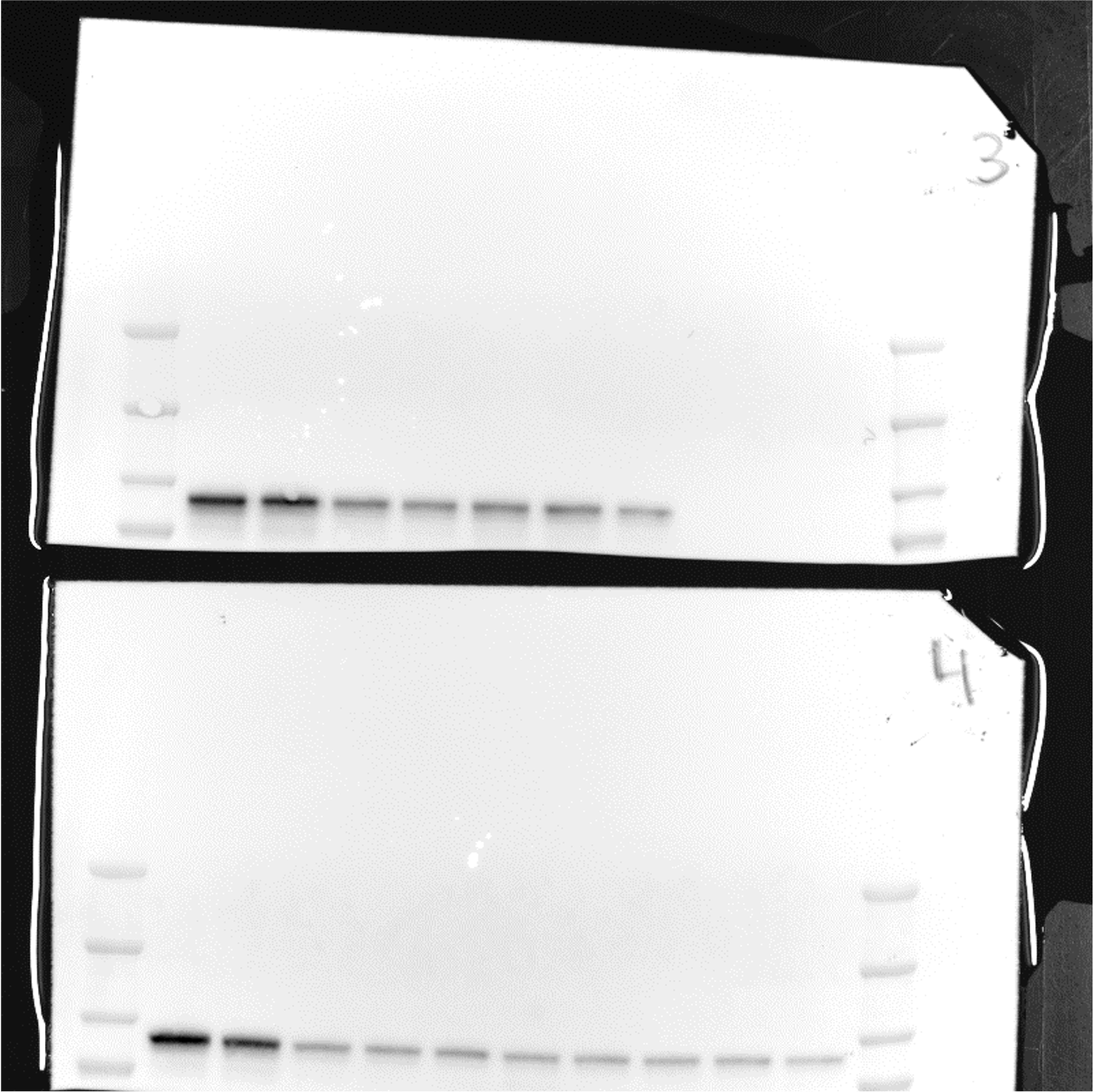

Supplement: Supplementary file 15 — EV and Appendix Figures Source Data [file 44321_2025_264_MOESM15_ESM.zip › EMM-2024-20682_SourceDataForExpandedViewAndAppendix/Sourcedata fig EV1/EV1C/EV1C_Western blot_GR_blot1+2.png]

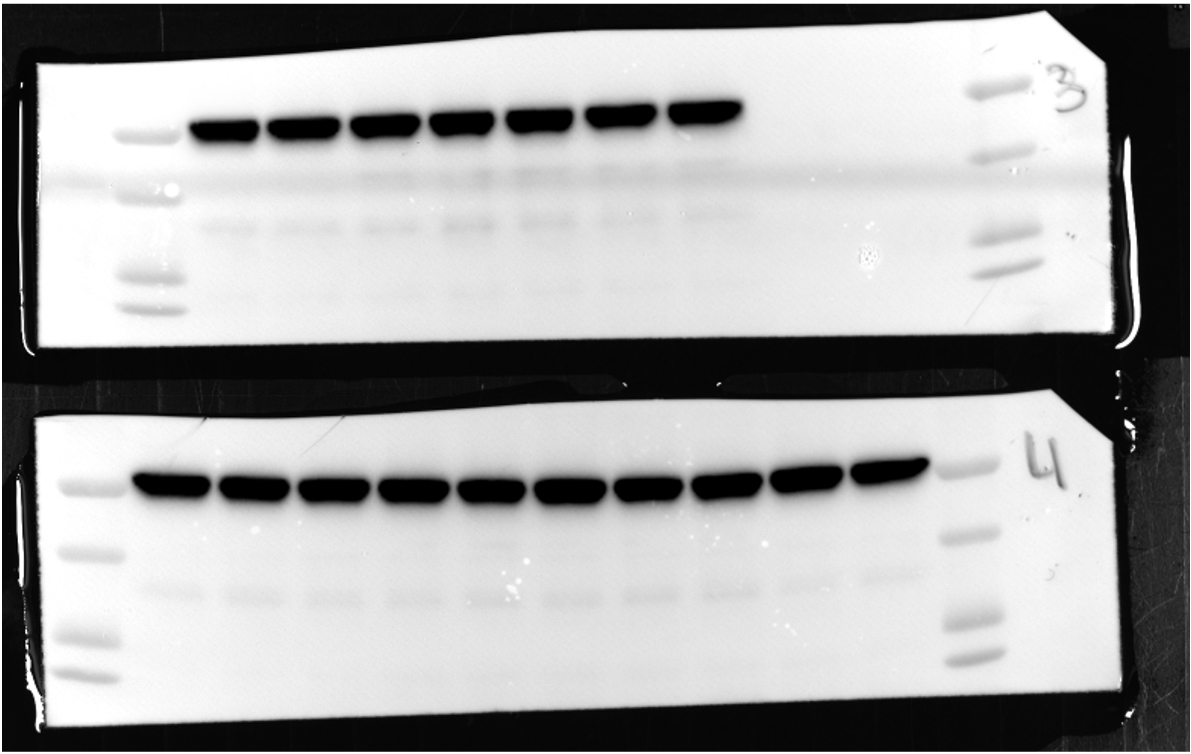

Supplement: Supplementary file 15 — EV and Appendix Figures Source Data [file 44321_2025_264_MOESM15_ESM.zip › EMM-2024-20682_SourceDataForExpandedViewAndAppendix/Sourcedata fig EV1/EV1C/EV1C_Western blot_Tubulin_blot1+2.png]

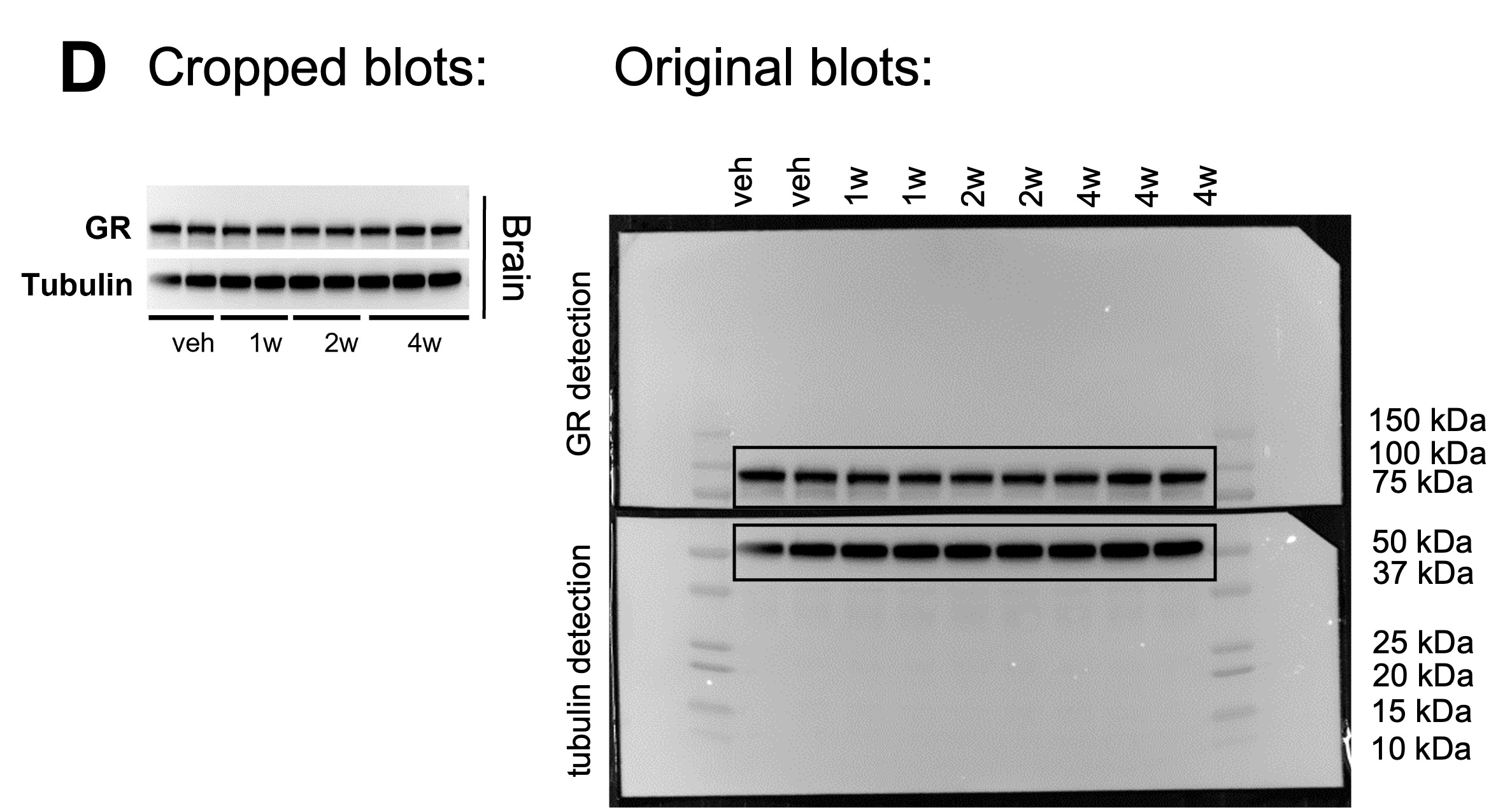

Supplement: Supplementary file 15 — EV and Appendix Figures Source Data [file 44321_2025_264_MOESM15_ESM.zip › EMM-2024-20682_SourceDataForExpandedViewAndAppendix/Sourcedata fig EV1/EV1D/EV1D_Cropped western blot image.jpg]

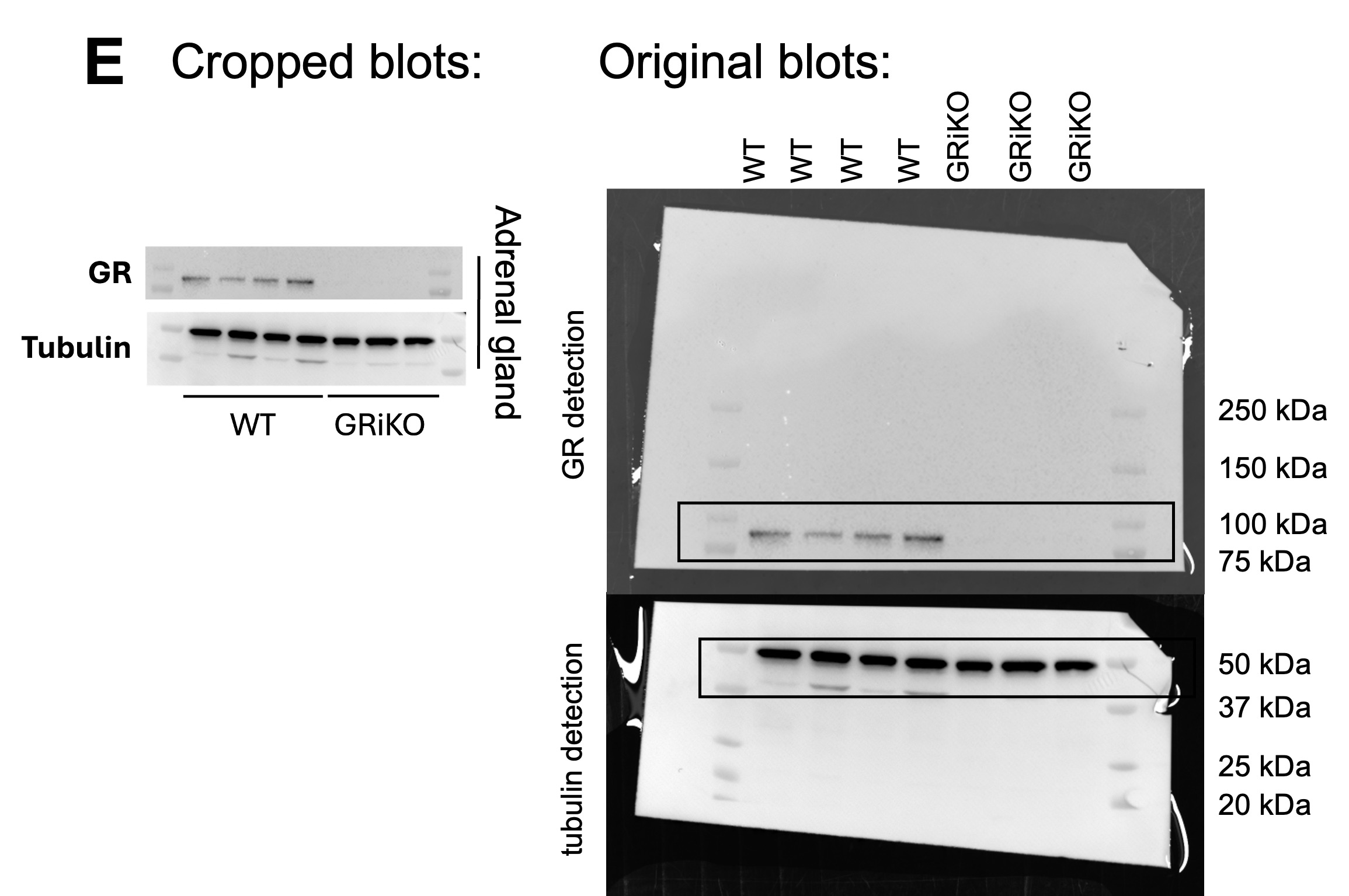

Supplement: Supplementary file 15 — EV and Appendix Figures Source Data [file 44321_2025_264_MOESM15_ESM.zip › EMM-2024-20682_SourceDataForExpandedViewAndAppendix/Sourcedata fig EV1/EV1E/EV1E_Cropped western blot image.jpg]

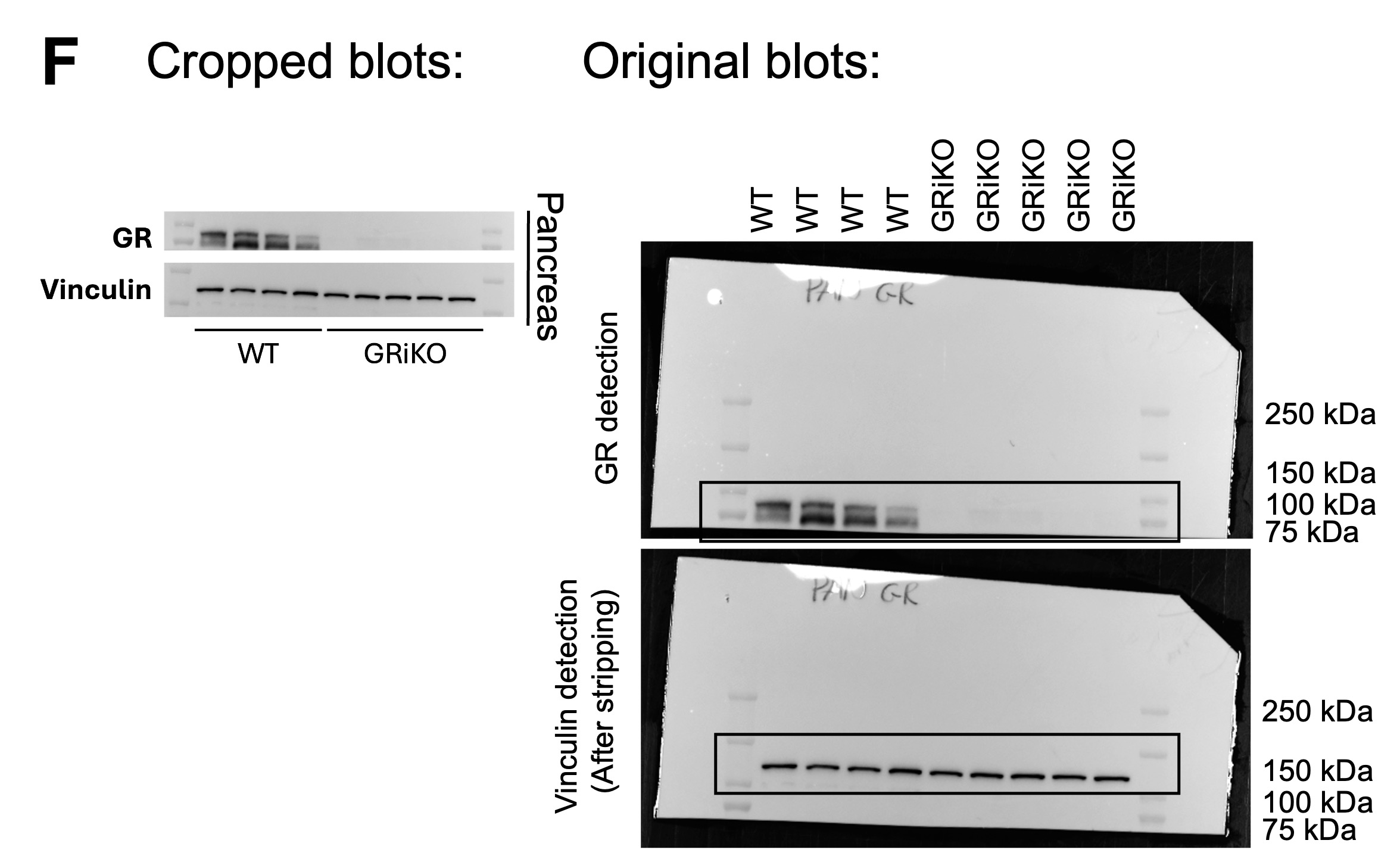

Supplement: Supplementary file 15 — EV and Appendix Figures Source Data [file 44321_2025_264_MOESM15_ESM.zip › EMM-2024-20682_SourceDataForExpandedViewAndAppendix/Sourcedata fig EV1/EV1F/EV1F_Cropped western blot image.jpg]

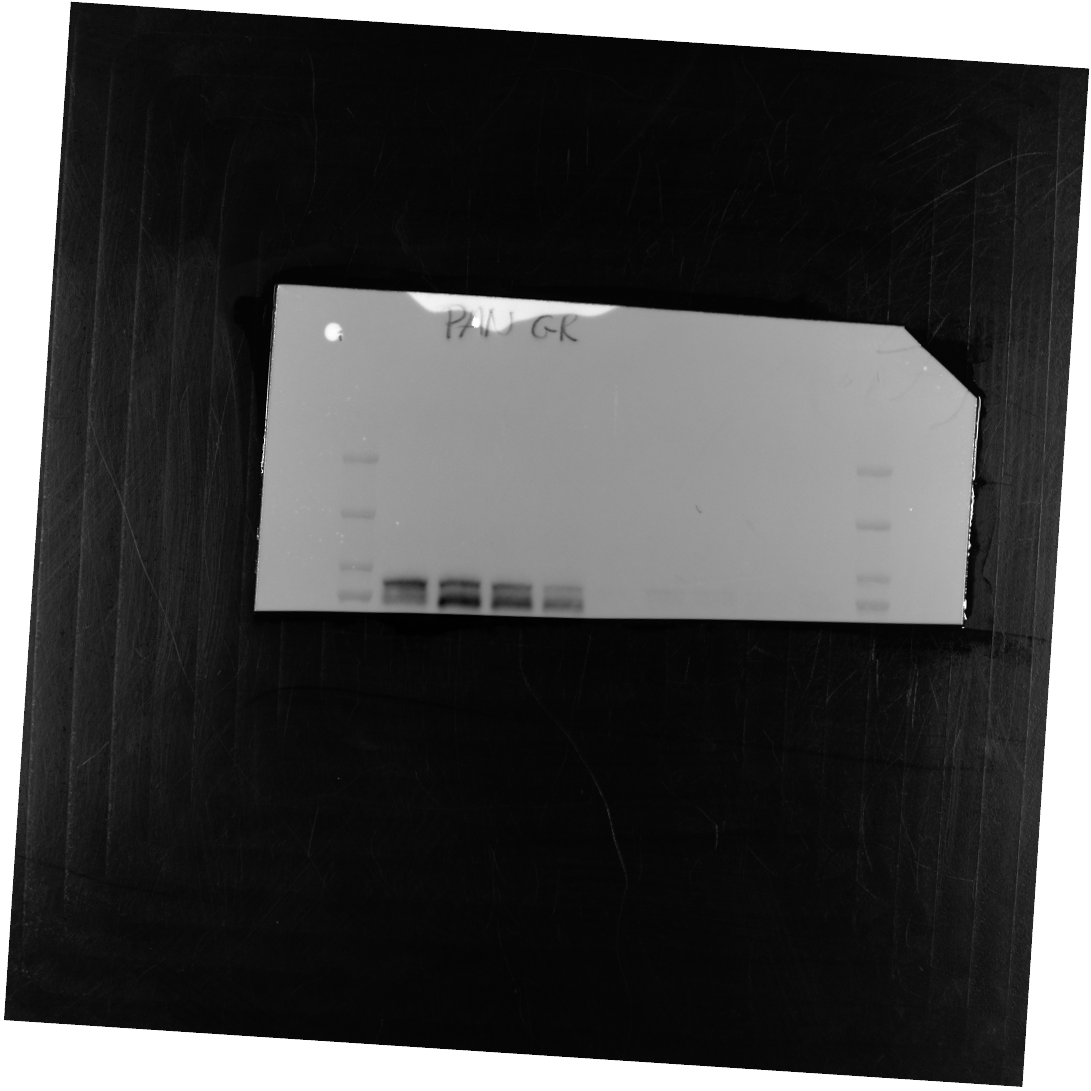

Supplement: Supplementary file 15 — EV and Appendix Figures Source Data [file 44321_2025_264_MOESM15_ESM.zip › EMM-2024-20682_SourceDataForExpandedViewAndAppendix/Sourcedata fig EV1/EV1F/EV1F_Western blot_Vinculin.png]

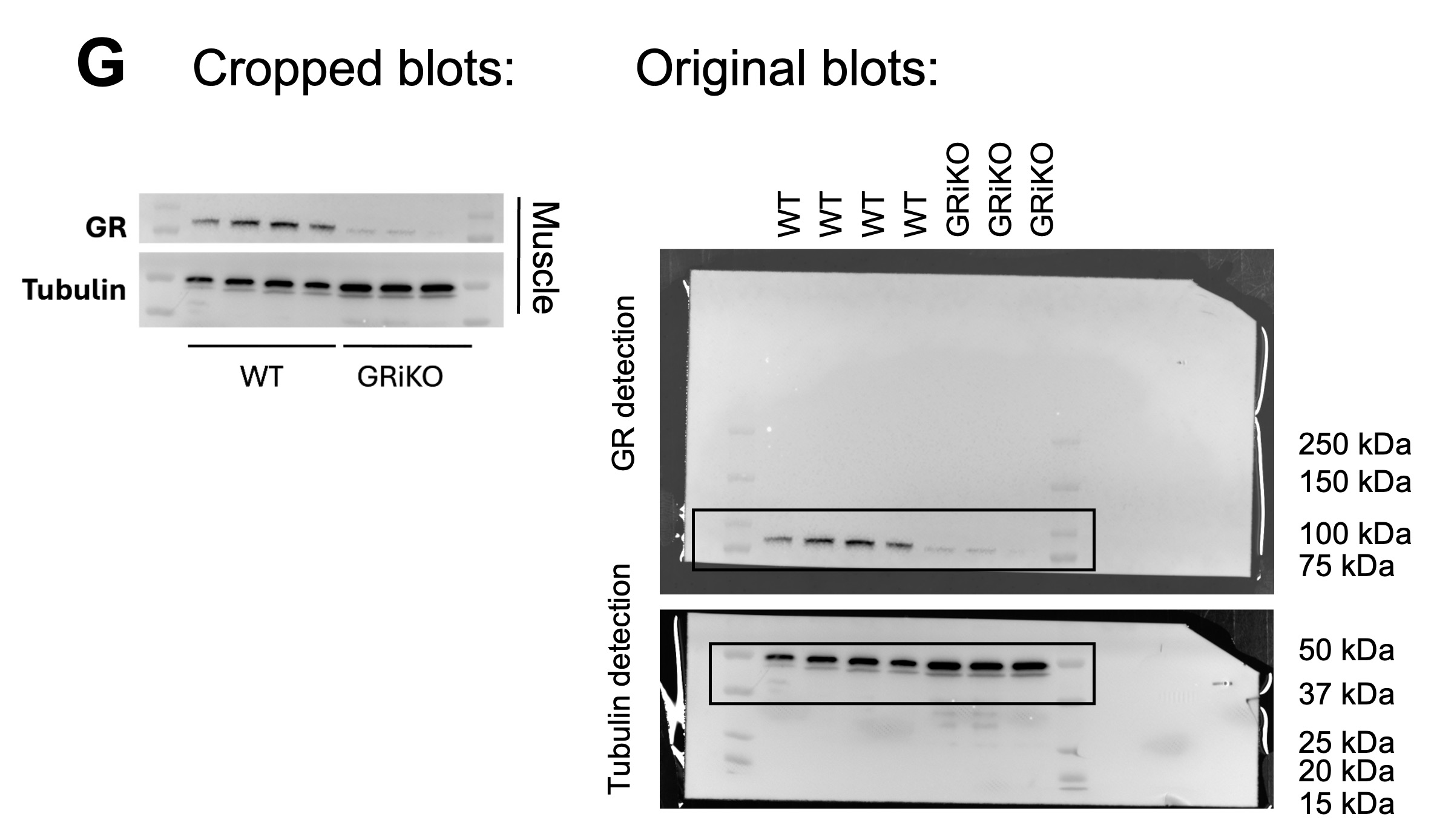

Supplement: Supplementary file 15 — EV and Appendix Figures Source Data [file 44321_2025_264_MOESM15_ESM.zip › EMM-2024-20682_SourceDataForExpandedViewAndAppendix/Sourcedata fig EV1/EV1G/EV1G_Cropped western blot image.jpg]

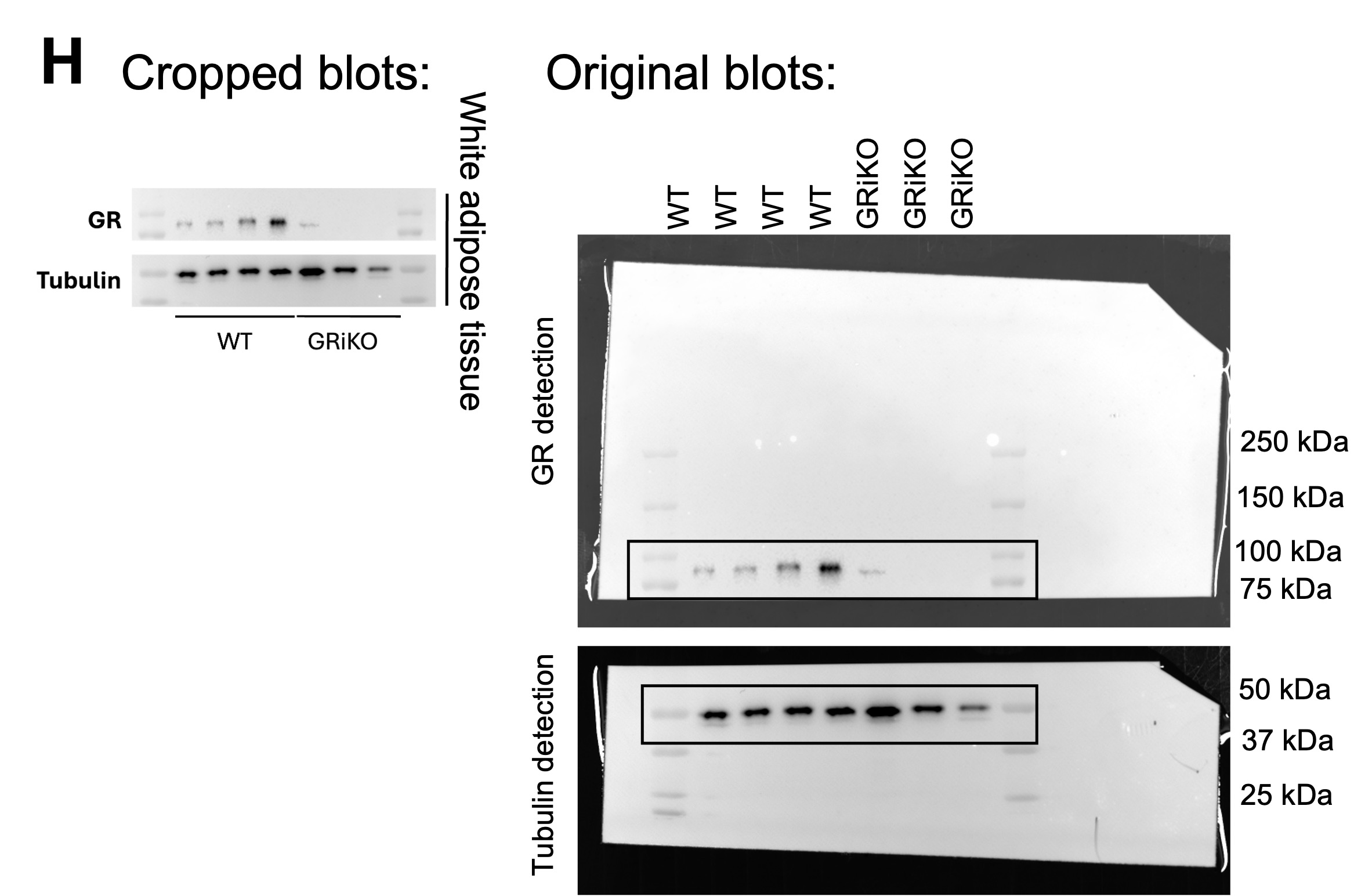

Supplement: Supplementary file 15 — EV and Appendix Figures Source Data [file 44321_2025_264_MOESM15_ESM.zip › EMM-2024-20682_SourceDataForExpandedViewAndAppendix/Sourcedata fig EV1/EV1H/EV1H_Cropped western blot image.jpg]

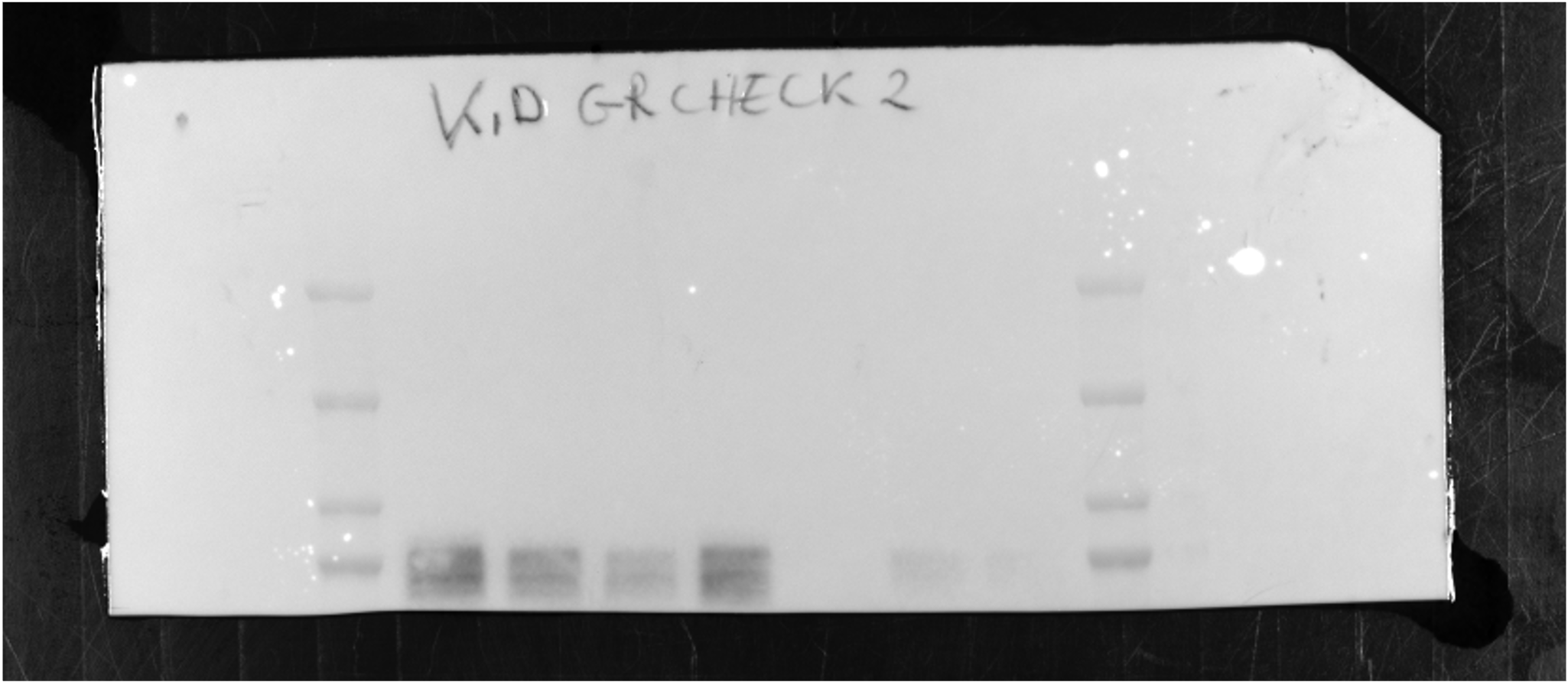

Supplement: Supplementary file 15 — EV and Appendix Figures Source Data [file 44321_2025_264_MOESM15_ESM.zip › EMM-2024-20682_SourceDataForExpandedViewAndAppendix/Sourcedata fig EV1/EV1I/EV1I_Western blot_GR.png]

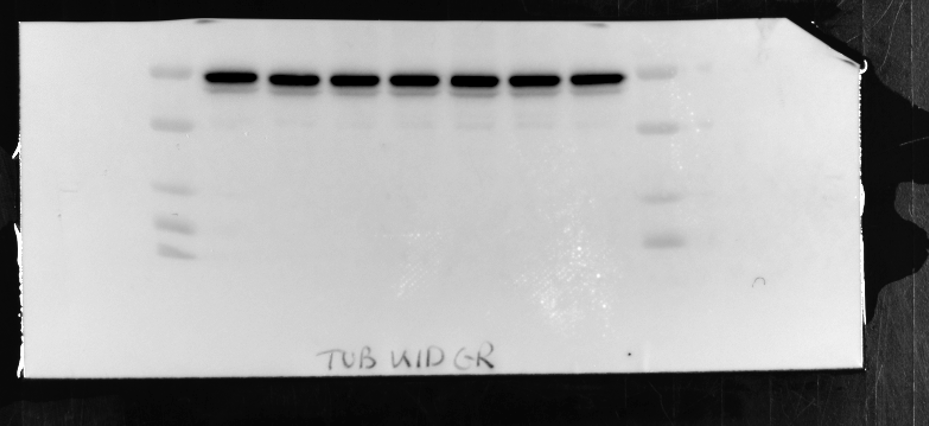

Supplement: Supplementary file 15 — EV and Appendix Figures Source Data [file 44321_2025_264_MOESM15_ESM.zip › EMM-2024-20682_SourceDataForExpandedViewAndAppendix/Sourcedata fig EV1/EV1I/EV1I_Western blot_Tubulin.png]
