# Supplementary material for: JAK/STAT inhibition protects glucocorticoid receptor knockout mice from lethal malaria-induced hypoglycemia and hyperinflammation
Source: EMBO Mol Med. 2025 Jul 23;17(8):2040–70. doi: 10.1038/s44321-025-00264-w (PMC12340059; doi:10.1038/s44321-025-00264-w)
Supplement: Supplementary file 16 — Expanded View Figures [file 44321_2025_264_MOESM16_ESM.pdf]

Expanded View Figures

Figure EV1. Validation of tamoxifen treatment for the induction of global GR deletion.

Tamoxifen-inducible WT and GRiKO mice were treated with vehicle or 10 mg/kg tamoxifen for 5 consecutive days. Organs were collected at 1 week, 2 weeks or 4 weeks after the last tamoxifen treatment was given. Protein expression of GRα were determined in (**A**) spleen, (**B**) liver, (**C**) lung, (**D**) whole brain, (**E**) adrenal gland, (**F**) pancreas, (**G**) quadriceps muscle, (**H**) white adipose tissue (WAT) and (**I**) kidney extracts. For (**A–D**): percentage of reduction in GRα protein expression in tamoxifen-treated GRiKO group relative to vehicle-treated GRiKO group. For (**E–I**): percentage of reduction in GRα protein expression in tamoxifen-treated GRiKO group relative to tamoxifen-treated WT group is shown. Representative blots are shown together with the semiquantitative determination of band intensities. Each datapoint represents an individual mouse. Data from one or two independent experiments. Bar graph represent median per group. Statistical differences were calculated with the Mann–Whitney *U* test. ns: $P > 0.05$. $*P < 0.05$. Source data are available online for this figure.

 © The Author(s)

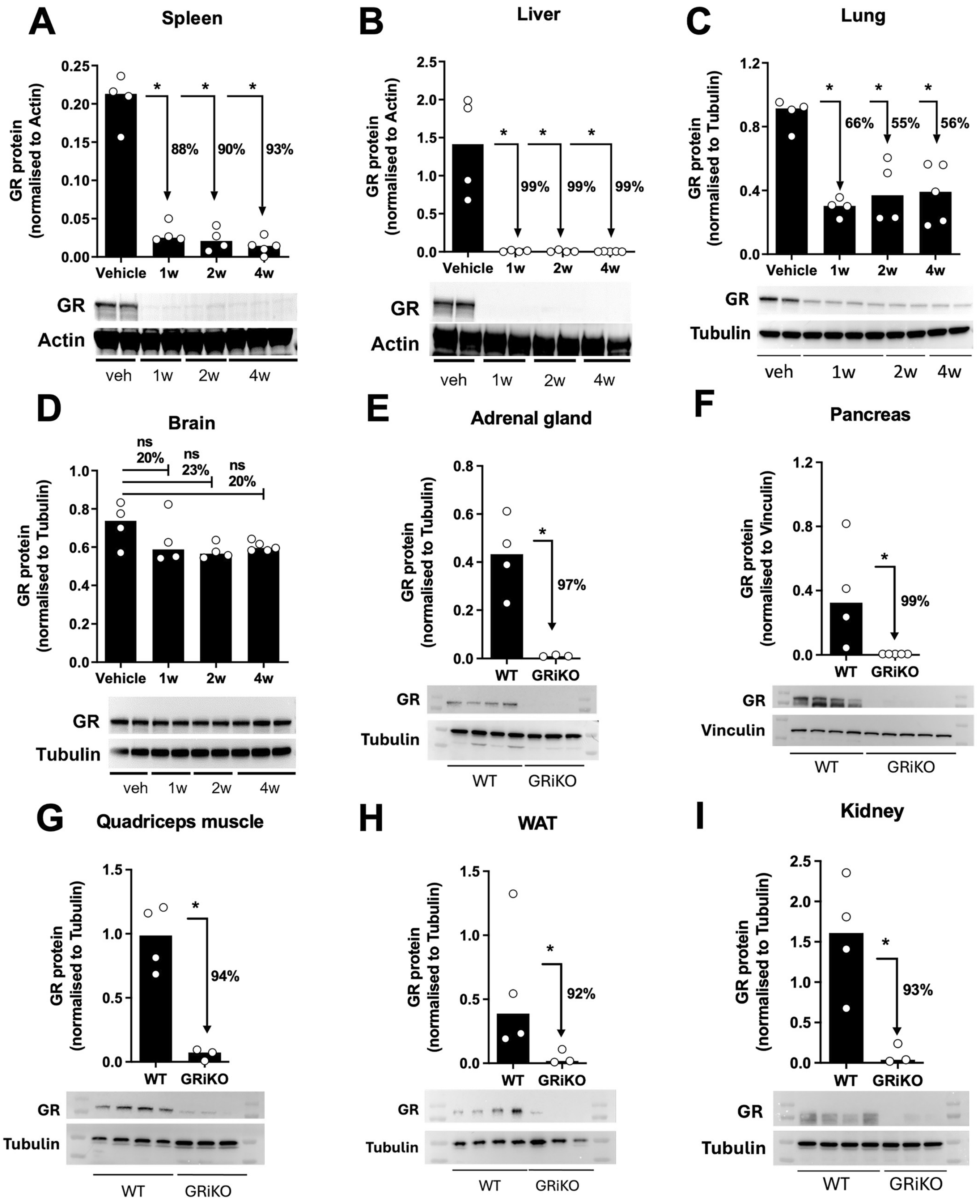
A
Spleen
GR protein (normalised to Actin)
0.25
0.20
0.15
0.10
0.05
0.00
*
88%
90%
93%
Vehicle 1w 2w 4w
GR
Actin
veh 1w 2w 4w
B
Liver
GR protein (normalised to Actin)
2.5
2.0
1.5
1.0
0.5
0.0
*
99%
99%
99%
Vehicle 1w 2w 4w
GR
Actin
veh 1w 2w 4w
C
Lung
GR protein (normalised to Tubulin)
1.2
0.8
0.4
0.0
*
66%
55%
56%
Vehicle 1w 2w 4w
GR
Tubulin
veh 1w 2w 4w
D
Brain
ns 20%
ns 23%
ns 20%
GR protein (normalised to Tubulin)
1.0
0.8
0.6
0.4
0.2
0.0
Vehicle 1w 2w 4w
GR
Tubulin
veh 1w 2w 4w
E
Adrenal gland
GR protein (normalised to Tubulin)
0.8
0.6
0.4
0.2
0.0
*
97%
WT GRiKO
GR
Tubulin
WT GRiKO
F
Pancreas
GR protein (normalised to Vinculin)
1.2
0.8
0.4
0.0
*
99%
WT GRiKO
GR
Vinculin
WT GRiKO
G
Quadriceps muscle
GR protein (normalised to Tubulin)
1.5
1.0
0.5
0.0
*
94%
WT GRiKO
GR
Tubulin
WT GRiKO
H
WAT
GR protein (normalised to Tubulin)
1.5
1.0
0.5
0.0
*
92%
WT GRiKO
GR
Tubulin
WT GRiKO
I
Kidney
GR protein (normalised to Tubulin)
2.5
2.0
1.5
1.0
0.5
0.0
*
93%
WT GRiKO
GR
Tubulin
WT GRiKO

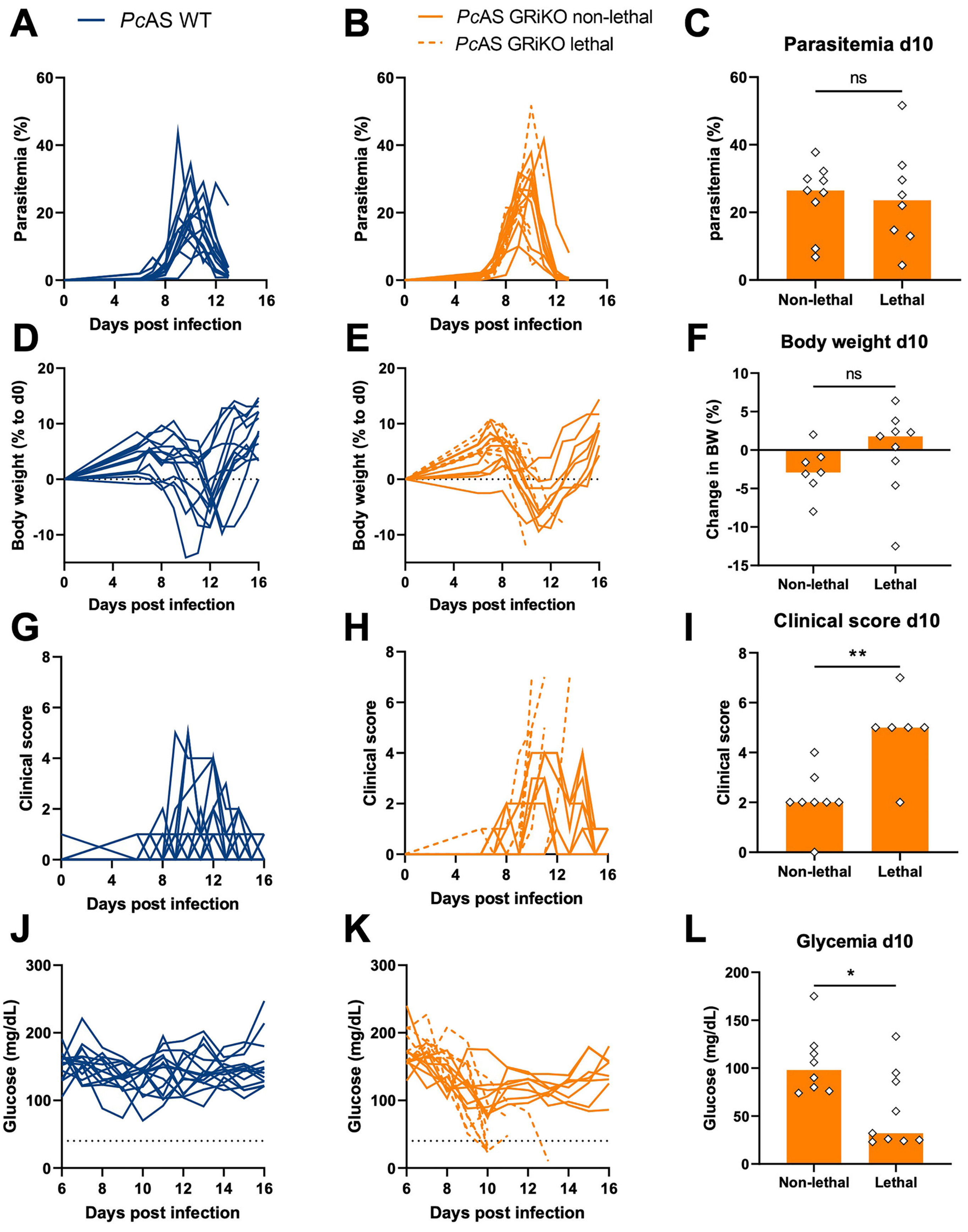
A
PcAS WT
B
PcAS GRiKO non-lethal
PcAS GRiKO lethal
C
Parasitemia d10
ns
Parasitemia (%)
parasitemia (%)
Days post infection
Non-lethal
Lethal
D
E
F
Body weight d10
Body weight (% to d0)
Change in BW (%)
G
H
I
Clinical score d10
**
Clinical score
J
K
L
Glycemia d10
*
Glucose (mg/dL)

 © The Author(s)

Figure EV2. The observed lethality in infected GRiKO mice is related to clinical score and glycemia, and not to parasitemia or body weight.

WT and GRiKO mice were infected with *Pc*AS parasites. (**A–C**) Parasitemia levels, (**D–F**) body weight, (**G–I**) clinical score and (**J–L**) glycemia were determined each day, from 6 dpi onwards. For each parameter, longitudinal data is represented. A statistical comparison was made for all four parameters (measured at 10 dpi) between lethal and non-lethal infected GRiKO mice (data from the same experiments as in Fig. 1C-E, I-J and Appendix Fig. S1C-E). Statistical differences were calculated with the Mann–Whitney *U* test. Each datapoint or line represents an individual mouse. Solid lines: non-lethal mice, and striped lines: lethal mice. Data from three independent experiments. Bar graph represent median per group. ns: $P > 0.05$, $*P < 0.05$, $**P < 0.01$.

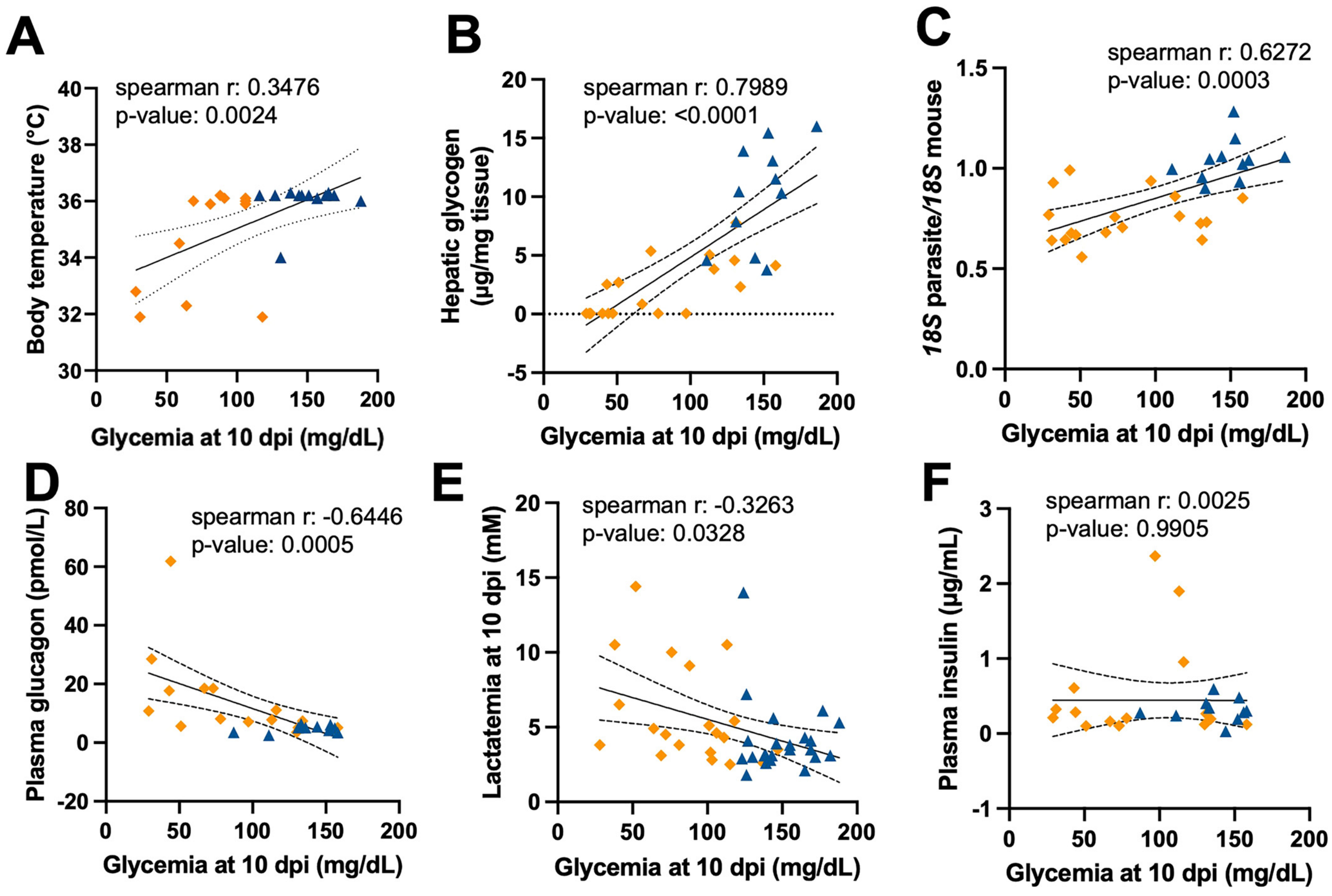

Figure EV3. Body temperature, hepatic glycogen, plasma glucagon, lactatemia and hepatic parasite load correlate with glycemia levels.

WT and GRiKO mice were infected with *Pc*AS parasites. At 10 dpi, glycemia, body temperature, hepatic glycogen, plasma glucagon levels and lactatemia were determined. Correlations were assessed between glycemia and (**A**) body temperature, (**B**) hepatic glycogen, (**C**) hepatic parasite load, (**D**) plasma glucagon, (**E**) lactatemia and (**F**) plasma insulin with a nonparametric Spearman test. ▲: infected WT mice and ◆: infected GRiKO mice. Each symbol represents an individual mouse. Data from two individual experiments.

 © The Author(s)

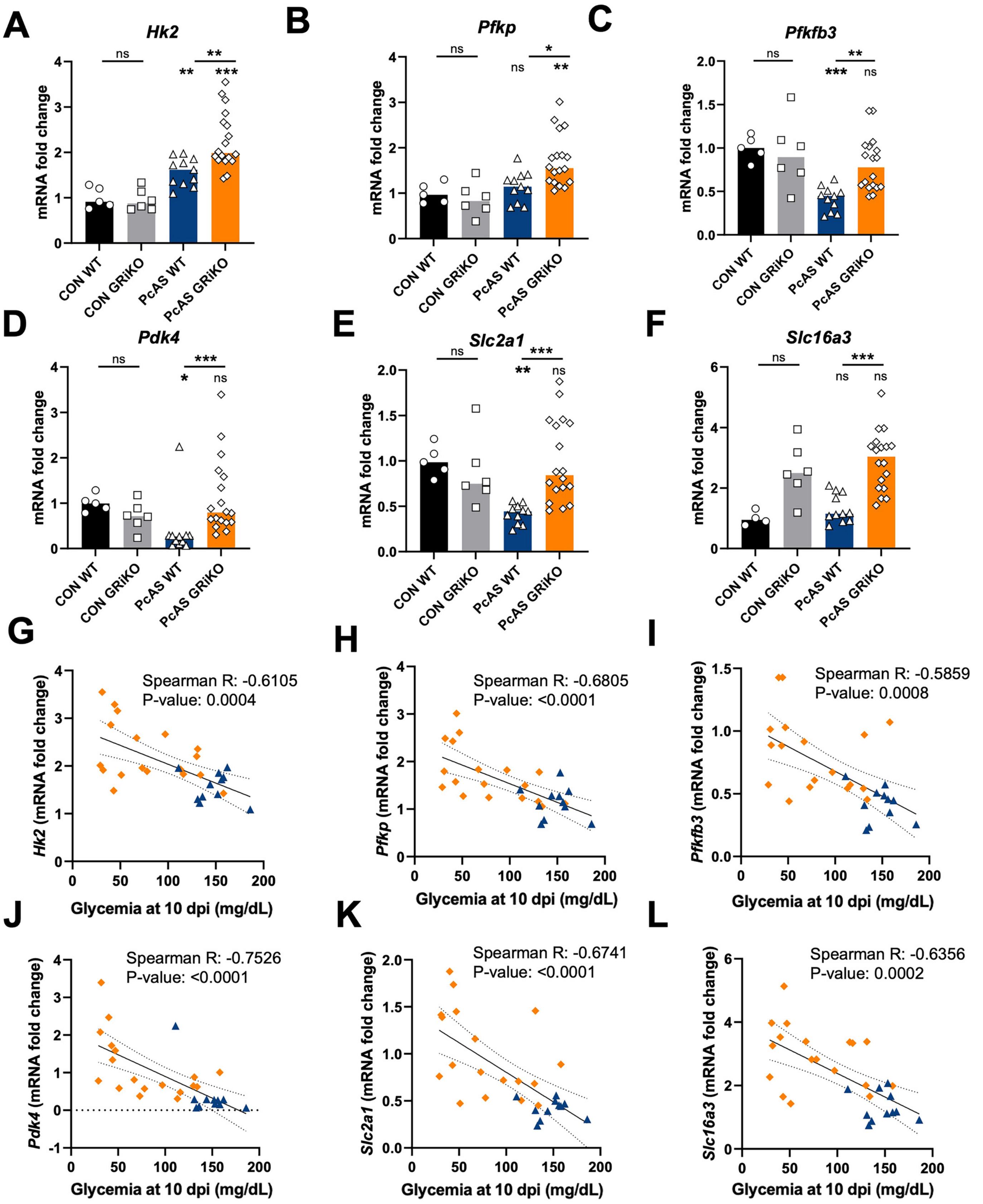
A
Hk2
B
Pfkp
C
Pfkfb3
D
Pdk4
E
Slc2a1
F
Slc16a3
mRNA fold change
CON WT
CON GRiKO
PcAS WT
PcAS GRiKO
G
Spearman R: -0.6105
P-value: 0.0004
Hk2 (mRNA fold change)
H
Spearman R: -0.6805
P-value: <0.0001
Pfkp (mRNA fold change)
I
Spearman R: -0.5859
P-value: 0.0008
Pfkfb3 (mRNA fold change)
J
Spearman R: -0.7526
P-value: <0.0001
Pdk4 (mRNA fold change)
K
Spearman R: -0.6741
P-value: <0.0001
Slc2a1 (mRNA fold change)
L
Spearman R: -0.6356
P-value: 0.0002
Slc16a3 (mRNA fold change)
Glycemia at 10 dpi (mg/dL)

Figure EV4. GR signaling inhibits glycolytic gene expression in spleen during infection.

WT and GRiKO mice were infected with *Pc*AS parasites and spleens were collected at 10 dpi. qPCR was performed to determine mRNA expression of (**A**) *Hk2*, (**B**) *Pfkp*, (**C**) *Pfkfb3*, (**D**) *Pdk4*, (**E**) *Slc2a1* and (**F**) *Slc16a3*. Data were normalized towards mouse-specific *18S* and the average expression of CON WT mice. Correlation analyses were performed between glycemia and (**G**) *Hk2*, (**H**) *Pfkp*, (**I**) *Pfkfb3*, (**J**) *Pdk4*, (**K**) *Slc2a1* and (**L**) *Slc16a3* with the nonparametric Spearman test. Statistical differences were calculated with the Mann–Whitney *U* test with Holm–Bonferroni correction. ns: $P > 0.05$, *$P < 0.05$, **$P < 0.01$, ***$P < 0.001$. Asterisks above data points indicate significant differences between infected and non-infected mice, asterisks above a horizontal line show significant differences between genotype. Each symbol represents an individual mouse and bar graph represent median per group. ▲: infected WT mice and ♦: infected GRiKO mice. Data from two independent experiments.

© The Author(s)

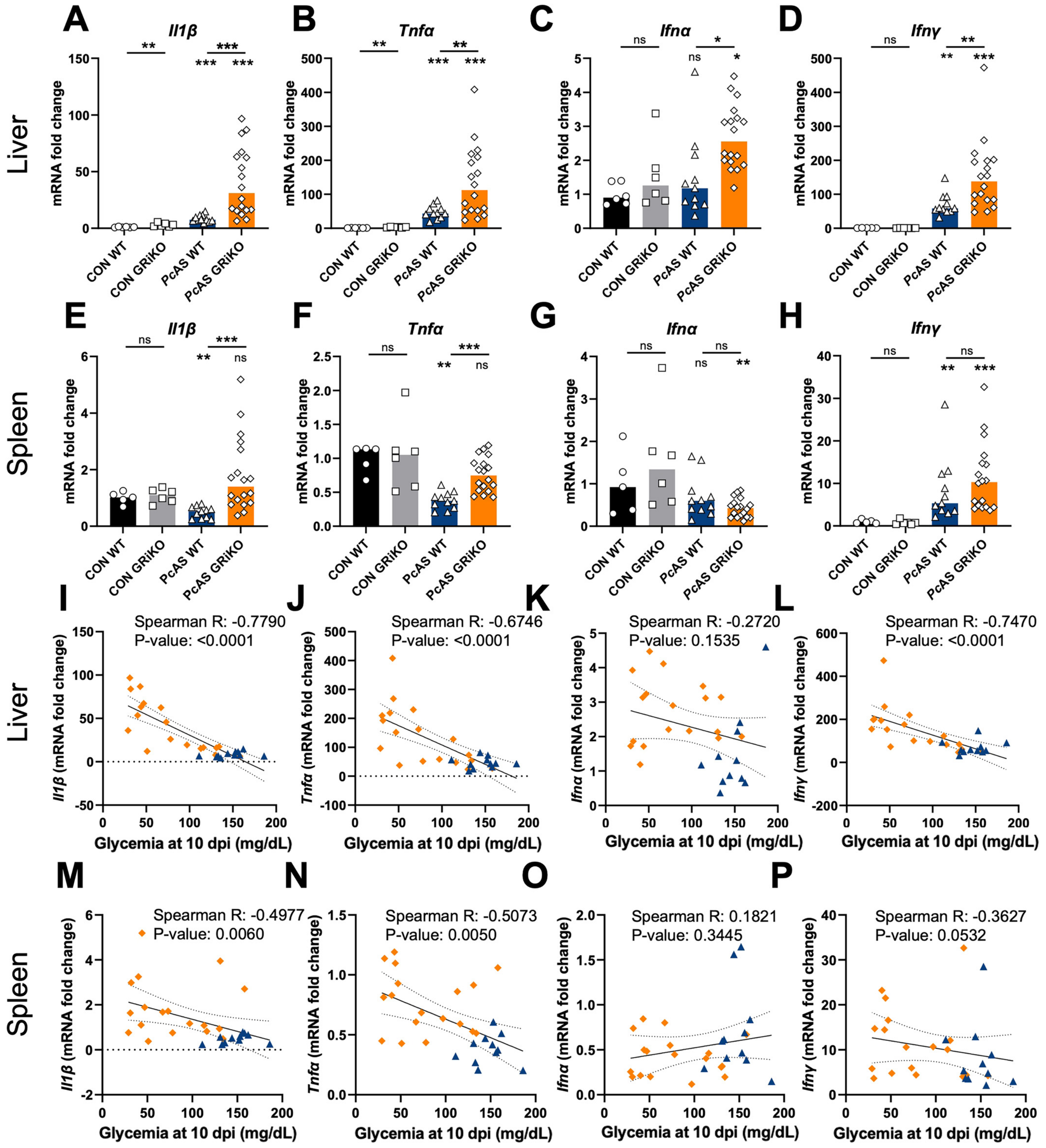

Figure EV5. GR signaling prevents cytokine storm in liver and spleen of *Pc*AS-infected mice.

WT and GRiKO mice were infected with *Pc*AS parasites. At 10 dpi, livers and spleens were collected and expression levels of (**A**, **E**) *Il1β* (**B**, **F**) *Tnfα*, (**C**, **G**) *Ifnα and* (**D**, **H**) *Ifnγ* were measured with qPCR. Data were normalized towards mouse-specific *18S* and the average expression of CON WT mice. (**I–P**) Correlations between glycemia levels and the cytokine expressions were assessed with the nonparametric Spearman test. Each symbol represents an individual mouse and bar graph represent median per group. ▲: infected WT mice and ◆: infected GRiKO mice. Statistical differences were calculated with the Mann–Whitney *U* test with Holm–Bonferroni correction. ns: $P > 0.05$, *$P < 0.05$, **$P < 0.01$, ***$P < 0.001$. Asterisks above data points indicate significant differences between infected and non-infected mice, asterisks above a horizontal line show significant differences between genotype. Data from two independent experiments.

© The Author(s)
